# A Cluster Randomized Study of The Safety of Integrated Treatment of Trachoma and Lymphatic Filariasis in Children and Adults in Sikasso, Mali

**DOI:** 10.1371/journal.pntd.0002221

**Published:** 2013-05-09

**Authors:** Yaya Ibrahim Coulibaly, Ilo Dicko, Modibo Keita, Mahamadou Minamba Keita, Moussa Doumbia, Adama Daou, Fadima Cheick Haidara, Moussa Hama Sankare, John Horton, Caroline Whately-Smith, Samba Ousmane Sow

**Affiliations:** 1 Centre National d'Appui à la Lutte contre la Maladie, Bamako, Mali; 2 Filariasis Unit at MRTC, Faculty of Medicine of Bamako, Mali; 3 Direction Nationale de la Sante, Bamako, Mali; 4 Tropical Projects, Hitchin, United Kingdom; 5 Whately-Smith Ltd, Kings Langley, United Kingdom; Centers for Disease Control and Prevention, United States of America

## Abstract

**Background:**

Neglected tropical diseases are co-endemic in many areas of the world, including sub Saharan Africa. Currently lymphatic filariasis (albendazole/ivermectin) and trachoma (azithromycin) are treated separately. Consequently, financial and logistical benefit can be gained from integration of preventive chemotherapy programs in such areas.

**Methodology/Findings:**

4 villages in two co-endemic districts (Kolondièba and Bougouni) of Sikasso, Mali, were randomly assigned to coadministered treatment (ivermectin/albendazole/azithromycin) or standard therapy (ivermectin/albendazole with azithromycin 1 week later). These villages had previously undergone 4 annual MDA campaigns with ivermectin/albendazole and 2 with azithromycin. One village was randomly assigned to each treatment arm in each district. There were 7515 eligible individuals in the 4 villages, 3011(40.1%) of whom participated in the study. No serious adverse events occurred, and the majority of adverse events were mild in intensity (mainly headache, abdominal pain, diarrhoea and “other signs/symptoms”). The median time to the onset of the first event, of any type, was later (8 days) in the two standard treatment villages than in the co-administration villages. Overall the number of subjects reporting any event was similar in the co-administration group compared to the standard treatment group [18.7% (281/1501) vs. 15.8% (239/1510)]. However, the event frequency was higher in the coadministration group (30.4%) than in the standard treatment group (11.0%) in Kolondièba, while the opposite was observed in Bougouni (7.1% and 20.9% respectively). Additionally, the overall frequency of adverse events in the co-administration group (18.7%) was comparable to or lower than published frequencies for ivermectin+albendazole alone.

**Conclusions:**

These data suggest that co-administration of ivermectin+albendazole and azithromycin is safe; however the small number of villages studied and the large differences between them resulted in an inability to calculate a meaningful overall estimate of the difference in adverse event rates between the regimens. Further work is therefore needed before co-administration can be definitively recommended.

**Trial Registration:**

ClinicalTrials.gov; NCT01586169

## Author Summary

Neglected tropical diseases are co-endemic in many areas of the world. Currently lymphatic filariasis (albendazole+ivermectin) and trachoma (azithromycin) are treated separately. Benefits can be gained from integration of preventive chemotherapy programs in such areas. To assess the safety of this approach, 4 villages in two co-endemic districts in Mali were randomly assigned to coadministered treatment (ivermectin/albendazole/azithromycin) or standard therapy (ivermectin/albendazole with azithromycin 1 week later). One village was randomly assigned to each treatment in each district. 3011(40.1%) of 7515 eligible individuals in the 4 villages participated in the study. No serious adverse events occurred, and most events were mild in intensity (mainly headache, abdominal pain, and diarrhoea). Overall the number of subjects reporting any event was similar with co-administration compared to standard treatment [18.7% (281/1501) vs. 15.8% (239/1510)]. The overall frequency of adverse events with co-administration was comparable to or lower than published frequencies for ivermectin/albendazole alone. These data suggest that co-administration is safe; however the small number of villages studied and the large differences between them meant that a meaningful estimate of the differences could not be calculated, and further work will be needed before a recommendation can be made.

## Introduction

Lymphatic filariasis (LF), a leading cause of permanent and long-term disability globally, affects over 120 million people in more than 80 countries in tropical and subtropical areas [1]. Trachoma is the main cause of infectious blindness, responsible for around 5% of the world's blind people [2]. These two infections represent important public health problems in West Africa. Mali alone, with a population of 14.5 million (2009), has approximately 300,000 people who are at risk of disability from these two diseases [3].

While the safety and efficacy of separate treatments for trachoma and LF are well documented, their integrated treatment still represents a global challenge. Azithromycin, an antibiotic, has been used safely for over 10 years in trachoma treatment programs [4], [5]. The coadministration of single doses of two anti-parasitic drugs, ivermectin and albendazole, is a standard and safe treatment of LF in African countries where onchocerciasis is co-endemic and has been shown to reduce transmission following several rounds of MDA [6]. Ivermectin is indicated on its own for the treatment of onchocerciasis and strongyloidiasis, and for LF in combination with albendazole [7]. Albendazole is indicated for soil transmitted nematode infections and for systemic helminth infections such as neurocysticercosis and echinococcosis. It is used to treat LF in combination with ivermectin in areas co-endemic for onchocerciasis and in combination with diethylcarbamazine in all other LF-endemic areas. Both ivermectin and albendazole have an excellent safety record when used alone and also, for the last 10 years, in combination [8], [9].

Currently it is recommended that the administration of azithromycin and ivermectin/albendazole be separated by 7 to 14 days in co-endemic areas [10], [11], thus increasing the number of subject contacts and therefore overall cost of the interventions. It is therefore desirable to consider coadministration of drugs for the two diseases if this can be shown to be safe. Integrating the treatment of LF and trachoma would present logistical, health (greater compliance through integration) and economic advantages, helping reduce the burden on an already strained healthcare system. Given the disabling consequences of untreated LF and blinding trachoma, the large populations at risk for both diseases and the resource constraints of the endemic countries, the potential to improve the lives of the patients, optimize available healthcare resources and maximize the number of people reached make the minimal risks of initiating this new three-drug treatment administration acceptable [12]. It is recognized that the current approach is effective, safe, and free from SAE [8], [9], [13], [14], [15]. However, before integration can occur, the safety of coadministration of the three drugs (azithromycin, ivermectin and albendazole) has to be examined.

The data to support such an approach is limited to a pharmacokinetic study [16] on the interaction of the three drugs (azithromycin, ivermectin and albendazole) conducted in the United States in 18 healthy volunteers in 2004. Coadministration resulted in an increase in systemic exposure (Cmax and AUC _0-t_) of azithromycin by 13% and 20% and in ivermectin exposure by 31% and 27% respectively. At the same time, albendazole sulfoxide exposure was decreased by 16% and 14% respectively. The changes in exposure to azithromycin and albendazole were considered not to be of clinical importance, while the increase in ivermectin exposure is of potential concern. Increased exposure to ivermectin could potentially lead to high levels in the brain, although a study in normal volunteers receiving 10 times the current dose did not show significant CNS toxicity [17], [18]. In this small population of normal volunteers, increased ivermectin exposure was not associated with an increased incidence of adverse events, such as dizziness, typically associated with ivermectin. One subject reported mild indigestion following administration of the ivermectin/albendazole combination. The other subject reported mild disequilibrium eight days after taking the three drugs together. Neither of these events required treatment. While these results are encouraging, they do not eliminate the risk of pharmacodynamic interactions or effects in infected patients.

Therefore given the outcome of the pharmacokinetic study in healthy volunteers and the expected increased cost-effectiveness of co-administration [12], the current pharmacovigilance study was undertaken under the auspices of the International Trachoma Initiative (ITI) with support from the Bill & Melinda Gates Foundation and conducted by the Center for Disease Control of Mali, to study the safety of coadministration of azithromycin, ivermectin and albendazole for the mass treatment of trachoma and LF in adults and children aged 5 and over in the Sikasso region of Mali.

## Materials and Methods

The study was an open label, community-based study comparing the standard treatments for trachoma (single dose azithromycin) and LF (ivermectin and albendazole) given 1 week apart with the coadministration of the three drugs. The study was registered with ClinicalTrials.gov with the reference NCT01586169.

### Ethics Statement

The protocol was approved by the Ethics Committee of the Faculty of Medicine, Pharmacy and Odontostomatology (FMPOS) of the University of Bamako, and Malian Ministry of Health through the National Directorate of Health and the Directorate of Pharmacy and Drugs. Once the study sites had been identified, the aim of the study and details of the procedures to be undertaken were presented through the different levels of administration to the level of the individual villages where meetings were held to provide community understanding of the protocol. Once community agreement had been obtained, a census of each village was undertaken to identify households, families and individuals, each of whom received a unique number. Subsequently, potential subjects in the study provided their own written consent to information provided on audiotape in Bambara (the local language). Parents or guardians provided written consent for all children participating in the study.

### Study Site

Mali was chosen because trachoma and LF were co-endemic and the baseline prevalence for both diseases from prior surveys was relatively high. In addition, the infrastructure existed to allow a study of this nature to be conducted.

In this study, the treatments were allocated to entire villages (clusters). The decision to use a cluster design was taken for logistical reasons. With the number of subjects involved, it would be very difficult to implement individual randomisation within a village. Furthermore, the study needed to assess the effects of mass treatment in the community as that is the target setting for the treatments under study. The villages chosen for the study needed to be accessible, for logistical reasons, with access to a health facility, but sufficiently far apart to avoid “contamination” between them. They had to be within 5 km of a serviceable road and within 15 km of a Community Health Centre (CSCOM) at the very peripheral level of the health system organization in Mali or a district Reference Health Center (CSREF) that is responsible for providing care to complicated cases referred from the different CSCOMs of the district. Villages also needed to be separated by at least 15 km and be on an East-West axis since the endemicity/epidemiology of LF is more homogeneous than on a North-South axis. They also needed to have populations of about 1200 people of local ethnic groups with similar socio-cultural habits. The anticipated exclusion rate was 25% so village populations needed to be large enough to allow for 750 subjects to be recruited to the study. Two districts, Bougouni and Kolondièba, in the Sikasso region of southern Mali (Figure S1), were selected for the study. Prior to study initiation, these districts had both received four annual standard treatments with albendazole and ivermectin for LF and two mass treatments with azithromycin for trachoma. It is likely that these interventions had considerably reduced the trachoma infection rate and filarial parasite load compared to historical data for the area. For logistical reasons, the infection rates were not re-evaluated immediately prior to the study

Trachoma is highly endemic in the Sikasso region and preliminary results of surveys conducted by ITI in June 2008 (ITI unpublished data, 2008), overall prevalence of trachoma was 14.65% in Bougouni and 24.4% in Kolondièba, while the prevalence in 1 to 9 year old children of the main stages of trachoma, Trachoma Inflammation-Follicular (TF) and 

Trachomatous Inflammation-Intense (TI) in Bougouni and Kolondièba were 5.8% (84/1456) and 7.5% (109/1453) respectively, a substantial reduction from earlier surveys [19]. The prevalence of LF in 2004 at before MDA was started was 19.8% in Bougouni and 22.4% in Kolondièba (National LF Elimination Programme, unpublished report). LF prevalence was not assessed in the study villages prior to the study initiation but data from neighbouring villages reported 14% and 24.4% respectively in the districts of Bougouni (Mena, Tienkoungoba) and Kolondièba (Bougoula, Kebila) (unpublished data, National LF Elimination Program, Mali).

Based on the selection criteria above, two villages in each district were chosen, Tienkoungoba (longitude −6.581690, latitude 11.456120) [standard treatment] and Ména (longitude −6.812560, latitude 11.527540) [co-administered treatment] in the District of Bougouni and the villages of Bougoula (longitude −7.094090, latitude 11.253810) [standard treatment] and Kebila (longitude −7.041090, latitude 11.276370) [co-administered treatment] in the District of Kolondièba. Allocation of treatment in each district was decided by the toss of a coin by the investigators following the investigators' training session in Bamako before the treatment phase field trip.

### Study Personnel and Training

Prior to initiation of the field based phases of the study (census and treatment/follow-up), the whole team was recruited and underwent intensive training to ensure that every member knew and understood their role. Since the treatment phase of the study was to be conducted simultaneously in the 4 villages, the data collection teams were all trained together on study methodology and especially the use of the predetermined adverse event questionaires. The details of the processes and the materials used may be found in the Supplimentary file ‘Study Protocol’ Section 9 pages 32–35, and Appendices D through K.

### Subjects

Following a complete census of the participating villages, all residents were invited to attend for screening. Male and female consenting/assenting subjects resident in the village for at least the previous 3 months and aged between 5 and 65 years were screened against inclusion criteria with a history and medical examination, which included documentation of signs/symptoms of trachoma (according to the WHO classification, and identified by health technicians specialised in ophthalmology) and LF (elephantiasis, lymphoedema, hydrocoele), prior to formal enrolment. Enrolment continued until the target number of subjects, approximately 40% of the total popualtion in each village, was reached. Blood tests for microfilaraemia or antigenaemia were not taken. All children over 5 years of age but under 90 cm in height and all pregnant and lactating women were excluded. In addition, all those who had significant acute or chronic illnesses or had a history of allergy to the drugs to be given were excluded. Subjects excluded after consent and non-enroled village residents were offered treatment after the study using the standard regimens for trachoma and LF according to national guidelines.

### Treatment and Follow-up

Following consent and screening, all enrolled subjects within a village received the drugs appropriate to the randomization. Patients were identified after randomization since the whole village was alocated to the same treatment. Subjects were aware of the treatment they would receive during the consent process. Appropriate doses for azithromycin and ivermectin were determined using a height pole marked with the doses on either side of the pole (Table 1). In the standard treatment villages, all subjects were treated on day 0, receiving ivermectin based on height (up to 4×3 mg tablets) and albendazole irrespective of height (1×400 mg tablet). They then received azithromycin based on height (up to 4×250 mg tablets) 7 days later. In the coadministration villages, all subjects received ivermectin based on height (up to 4×3 mg tablets), albendazole irrespective of height (1×400 mg tablet) and azithromycin based on height (up to 4×250 mg tablets) on day 1. No treatment was given 7 days later. All treatment administration was witnessed by study staff to ensure the medications were taken. Study staff were not blinded to the treatment administration.

**Table 1:** Dosing regimens for azithromycin, ivermectin and albendazole, according to height.

Participant height (cm)	Azithromycin (250 mg tablets)	Ivermectin (3 mg tablet)	Albendazole (400 mg tablet)
Less than 90	None	None	None
90–94	**1 tablet**	**1 tablet**	**1 tablet**
95–119	**2 tablets**	1 tablet	1 tablet
120–123	2 tablets	**2 tablets**	1 tablet
124–140	**3 tablets**	2 tablets	1 tablet
141–143	3 tablets	**3 tablets**	1 tablet
144–158	**4 tablets**	3 tablets	1 tablet
More than 159	4 tablets	**4 tablets**	1 tablet

Note that at each height increment >90 cm the dosage of only 1 of the 3 medications changes (indicated in bold). Height was determined using a marked pole, with separate graduations (one side for azithromycin and one for ivermectin). The sides were easily differentiated using words and symbols.

Subjects were seen and assessed on Days 1, 8 and 15. On Day 0, in addition to a general examination, they were asked about any complaints from a pre-specified list (e.g. abdominal pain, headaches) or others (recorded verbatim) that existed prior to treatment. On Days 8 and 15, the subjects underwent a further clinical examination and were asked whether they had experienced any AE since the administration of the drug, or whether any complaints present prior to treatment had changed in any way. Subjects were also able to present themselves at the health centres, which were manned throughout the study, if they were concerned about any events between the planned assessment visits. All AEs were classified either as ‘minor’ (not interfering with daily function) or ‘major’ (interfering with daily function). If AEs required treatment, this was based on the presenting signs and symptoms.

Study investigators were located in village health centers or at other appropriate sites recommended by villagers. Subjects went to the agreed site to be assessed for eligibility for the study. Households were grouped by sector and assigned investigators with the aid of volunteers chosen by the villagers. These volunteers also helped check that all households had been surveyed and assisted in finding those who did not return for study assessments on Days 8 and 15. Any subjects who had not returned for the Day 8 assessment by Day 14 were excluded from the study. Similarly, any subjects not returning for the Day 15 assessment by Day 17 were excluded.

### Statistical Methods

The study was designed to show that there was no difference in the overall incidence of AE between the standard and coadministered treatment. Previous data on azithromycin suggested an AE rate of 5% [20], [21]. The incidence of AE with coadministered treatment was estimated as 8%. With power of 80% and a two-sided 95% confidence interval for the difference between rates, the sample size chosen was 1125 subjects per treatment. The sample size per group was increased to 1328 to allow for an 18% refusal rate. This was in turn rounded up to 1500 per group to increase the power. This required approximately 750 subjects per village in order to produce clusters of equal size. The original sample size calculations did not take into account that the study was based on clusters (i.e., villages), where all subjects within the same cluster received the same treatment and was thus grossly underpowered. Analysis was therefore conducted at an individual, rather than a cluster level.

The objective of the study was to assess safety of the three drugs when coadministered. The primary outcome was therefore the overall incidence of any AE. Any complaint, either one itemized in a pre-prepared list in the case record form (CRF), or any other untoward event occurring after treatment administration, or an existing complaint which worsened after drug administration, was considered as an AE. Each event was classified as minor (not interfering with daily function), moderate (interfering to some extent with daily function) or major (significant effect on daily function). The timing of each event was derived as the first day on which the event was recorded, calculated from the day of first drug administration. The duration was also calculated as the number of days from first appearance to last appearance (inclusive).

The incidence of each event itemized on the list in the CRF was calculated by counting the number of subjects who reported it at any time during the study. The incidence of any AE was derived in the same way, by counting the number of subjects reporting at least one event, whether on the list or not, at some stage during the study. Subjects reporting several events were only counted once. The time to first onset for each subject was calculated as the day on which the first AE of any kind occurred. The maximal severity for each subject was calculated as the maximal severity of any event reported during the study.

Since the study design did not fully account for the cluster design, and since there were only two clusters for each treatment, the scope for statistical analysis was limited. The overall incidence of AE in each village was calculated as a simple percentage of the total number of subjects who received treatment. A 95% confidence interval was calculated using exact methods if necessary (Wilson method, [22]). Note that even if there are no occurrences of a specific event in the study, the prevalence can still be estimated with its 95% confidence interval. The time to first onset of events in each village was investigated using survival analysis methods. Data from any subject reporting no AE by the end of the surveillance period was considered to be censored at the time of last assessment. The analysis was repeated for those subjects who experienced any AE and the median time to first onset was estimated with its 95% confidence interval. The severities of events were summarized in a table.

The frequency of occurrence of each event itemized in the CRF was summarized for each treatment within each district in tables and bar charts showing the percentage of the study population within the village who reported the event. The incidence of each type of event for each village was also shown with associated 95% confidence intervals graphically, with a separate plot for each type of event. The time to onset of the most frequently occurring events was also investigated using survival analysis methods.

The age-sex distribution of the study population within each village was compared to the overall village population using graphs.

The intra-class correlation coefficient and associated 95% confidence interval was calculated using the ANOVA estimator [23]. Due to the small number of clusters, the resulting estimate should be treated with caution.

## Results

### Study Population

**Figure 1:**
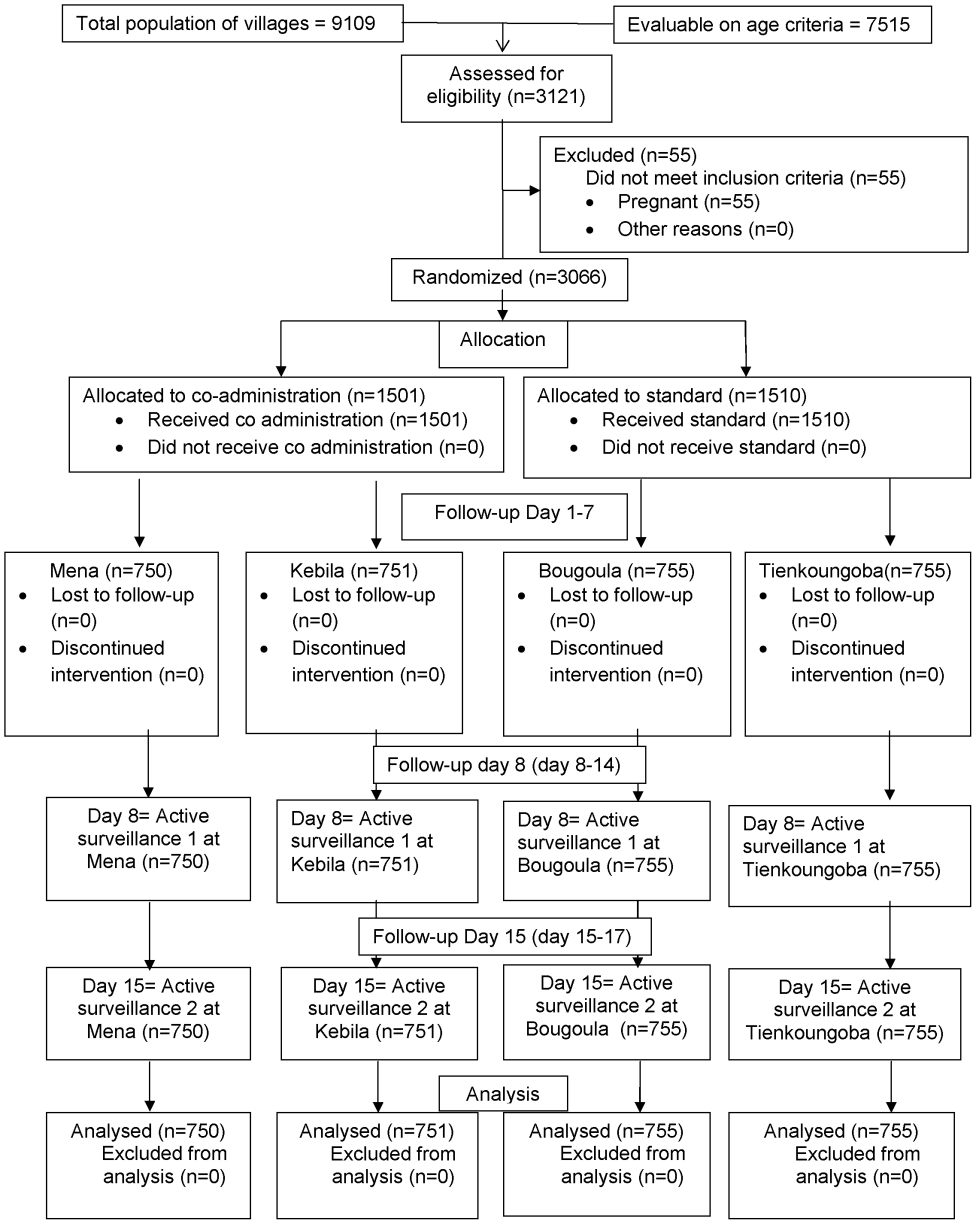
Disposition of subjects.

There were 9109 persons in the villages in the two districts of whom 7515 were eligible for inclusion (aged between 5 and 65 years, inclusive). 3011 subjects were chosen at random from this pool to be included in the study population (Table 2), selection continuing until the target number in a village was reached, with 1510 receiving the standard treatment (755 in each district) and 1501 receiving the coadministered treatment (750 in Bougouni, 751 in Kolondièba) (Figure 1). Recruitment of subjects started on February 7, 2010 with the first treatment being administered the same day while the final subject assessment was done on February 26, 2010. The recruitment took 3 to 4 days per village and all the villages began the recruitment on the same day

**Table 2:** Demographic summary for the study population in the 4 villages according to treatment.

	BOUGOUNI				KOLONDIÈBA			
	Standard		Co-administered		Standard		Co-administered	
	n	(%)	n	(%)	n	(%)	n	(%)
**Sex**								
**Female**	373	(49.4%)	355	(47.3%)	360	(47.7%)	382	(50.9%)
**Male**	382	(50.6%)	395	(52.7%)	395	(52.3%)	369	(49.1%)
**Age group**								
**Under 10**	254	(33.6%)	252	(33.6%)	236	(31.3%)	222	(29.6%)
**10–19**	281	(37.2%)	259	(34.5%)	291	(38.5%)	301	(40.1%)
**20–29**	33	(4.4%)	39	(5.2%)	53	(7.0%)	39	(5.2%)
**30–39**	36	(4.8%)	49	(6.5%)	33	(4.4%)	51	(6.8%)
**40–49**	53	(7.0%)	67	(8.9%)	69	(9.1%)	67	(8.9%)
**50 and over**	98	(13.0%)	84	(11.2%)	73	(9.7%)	71	(9.5%)
**Not native to village**	3	(0.4%)	2	(0.3%)	25	(3.3%)	33	(4.4%)
**Local**	752	(99.6%)	748	(99.7%)	730	(96.7%)	718	(95.6%)
**Total**	755	(100.0%)	750	(100.0%)	755	(100.0%)	751	(100.0%)

The male to female ratio was approximately equal in all villages (Table 2). In all villages, the proportion of males included in the study was slightly higher than the overall proportion of males in the village, with the proportion of females being correspondingly lower. The village allocated the coadministered treatment in the Kolondièba district (Kebila) was larger than the rest, hence the study population formed a smaller proportion of the total village population.

The age distribution of the study population in each village was similar (Table 2), with approximately 70% aged under 19 years and 10.8% aged over 50 years which represents the age structure within the villages as a whole. The age ranged from 5 to 65 years in all villages, with a mean of 20 years and a median of 12 years (Bougouni) or 13 years (Kolondièba).

The majority of study subjects were from the treatment villages (Table 2). The proportion of outsiders recruited to the study in Kolondièba was slightly higher (3.9%) than in Bougouni (0.3%), although the majority came from neighbouring villages and had been domiciled in the treatment villages for at least 3 months. None came from non-endemic areas. The height and weight distributions were similar for the four villages.

Slightly less than 5% of the total study population were found to have signs of trachoma. Two of the villages (Bougouni standard and Kolondièba coadministered treatment) had low rates of trachoma (0.9% and 1.9%) while the other two (Bougouni coadministered and Kolondièba standard treatment) had higher rates (6.0% and 8.7%). For these latter two villages the most frequent types of trachoma were follicular and scarring trachoma (eyelid scarring). 12 of the 14 subjects in Kebila (Kolondièba, coadministered treatment) had follicular trachoma. No subject in any of the villages had corneal opacity. Other findings from the eye examination were seen for between 0.8% (Kolondièba, coadministered treatment) and 5.9% (Bougouni, coadministered treatment). The pattern was similar as seen for the overall incidence of trachoma, with a lower level of other findings for Bougouni standard and Kolondièba coadministered treatment (less than 3% of subjects) and higher levels for Bougouni coadministered and Kolondièba standard treatment (above 4%). The most frequently occurring findings were chronic tropical endemic limboconjunctivitis (TELC), cataract, pterygium and conjunctivitis.

The number of abnormal findings from the LF examination was very low: 12 subjects with hydrocoele (6 on each treatment). One subject (Kolondièba, coadministered treatment) had both hydrocoele and lymphoedema: this was the only subject to have lymphoedema.

### Treatment Dosing and Compliance

The number of tablets administered was almost always correct according to height. There were more dosing errors for azithromycin than for ivermectin. There was a slightly higher error rate for the administration of the coadministered drugs than for the standard treatment: 13 subjects (0.9%) and 27 subjects (1.8%) had incorrect numbers of ivermectin and azithromycin tablets respectively in the coadministered groups compared to 3 (0.2%) and 12 (0.8%) for the standard treatments.

There were two instances of regurgitation of drugs on Day 0, both with the coadministered treatment. There were also two cases on Day 8 (azithromycin only). The drugs were not taken again in only one case. The majority, at least 95%, of subjects in all villages except Kebila (Kolondièba, coadministered treatment) took their drugs after a meal: in Kebila, only 577 subjects (76.8%) took their drugs after a meal. On Day 8, all the subjects with data recorded took their medication (azithromycin only) after a meal; 2 subjects had no data on time of drug intake recorded.

There were no losses to follow-up, all subjects allocated to treatment being evaluated within the timelines established for the protocol.

### Complaints Prior to Treatment

**Figure 2:**
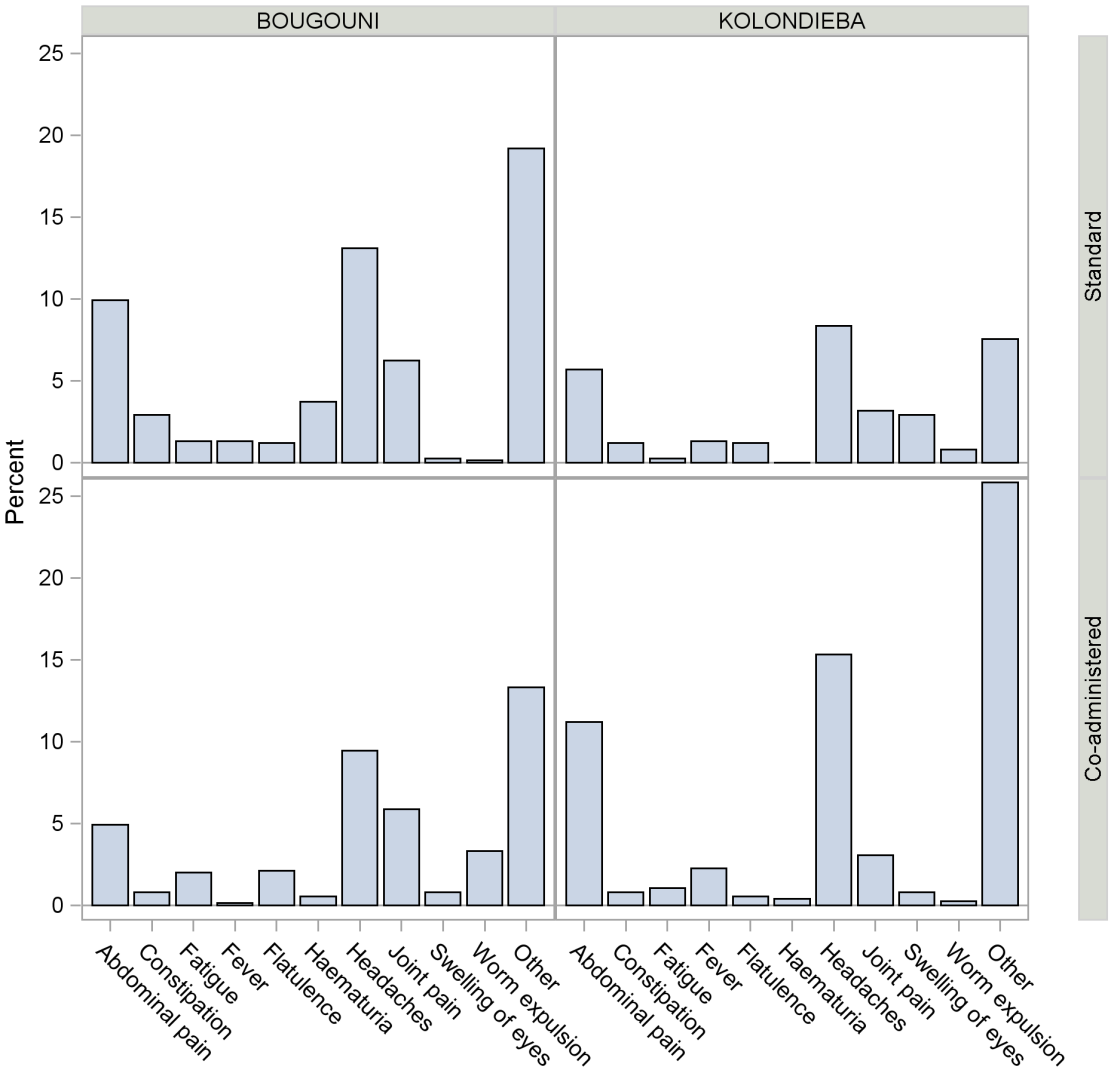
Complaints at baseline. Graph shows the incidence of specific complaints reported by 1% or more of subjects at baseline. Percentages are calculated using the total number of study subjects in each village. “Other” comprises any complaints not listed in the Case Report Form.

Table 3 summarizes the prevalence of complaints reported prior to treatment administration, with the most frequently occurring shown in Figure 2. The prevalence ranged from 26.2% (Kolondièba, standard treatment) to 46.9% (Bougouni, standard treatment). The most frequently reported of the complaints named in the CRF were headaches (348 subjects, 162 on standard treatment, 186 on coadministered treatment) with prevalence ranging from 8.3% (Kolondièba, standard treatment) to 15.3% (Kolondièba, coadministered treatment), and abdominal pain (239 subjects, 118 on standard treatment, 121 on coadministered treatment), with prevalences ranging from 4.9% (Bougouni, coadministered treatment) to 11.2% (Kolondièba, coadministered treatment).

**Table 3:** Complaints recorded prior to treatment (Day 0).

	BOUGOUNI				KOLONDIÈBA			
	Standard		Co-administered		Standard		Co-administered	
	n	(%)	n	(%)	n	(%)	n	(%)
**Total number of subjects**	755	(100.0%)	750	(100.0%)	755	(100.0%)	751	(100.0%)
**Any complaint**	354	(46.9%)	263	(35.1%)	198	(26.2%)	331	(44.1%)
**Headaches**	99	(13.1%)	71	(9.5%)	63	(8.3%)	115	(15.3%)
**Fever**	10	(1.3%)	1	(0.1%)	10	(1.3%)	17	(2.3%)
**Tinnitus**	4	(0.5%)	2	(0.3%)	8	(1.1%)	3	(0.4%)
**Deafness**	6	(0.8%)	5	(0.7%)	5	(0.7%)	0	(0.0%)
**Jaundice**	0	(0.0%)	2	(0.3%)	0	(0.0%)	0	(0.0%)
**Weakness**	1	(0.1%)	0	(0.0%)	0	(0.0%)	0	(0.0%)
**Fatigue**	10	(1.3%)	15	(2.0%)	2	(0.3%)	8	(1.1%)
**Nausea**	6	(0.8%)	8	(1.1%)	10	(1.3%)	4	(0.5%)
**Vomiting**	3	(0.4%)	1	(0.1%)	0	(0.0%)	3	(0.4%)
**Diarrhea**	4	(0.5%)	1	(0.1%)	1	(0.1%)	2	(0.3%)
**Abdominal pain**	75	(9.9%)	37	(4.9%)	43	(5.7%)	84	(11.2%)
**Flatulence/dyspepsia**	9	(1.2%)	16	(2.1%)	9	(1.2%)	4	(0.5%)
**Constipation**	22	(2.9%)	6	(0.8%)	9	(1.2%)	6	(0.8%)
**Joint/muscular pain**	47	(6.2%)	44	(5.9%)	24	(3.2%)	23	(3.1%)
**Swelling of (upper/lower) limbs**	0	(0.0%)	0	(0.0%)	1	(0.1%)	0	(0.0%)
**Swelling of eyelids/abnormal feeling in eyes**	2	(0.3%)	6	(0.8%)	22	(2.9%)	6	(0.8%)
**Rash/plaque**	0	(0.0%)	1	(0.1%)	0	(0.0%)	0	(0.0%)
**Scrotal reaction**	1	(0.1%)	0	(0.0%)	1	(0.1%)	0	(0.0%)
**Skin nodules**	0	(0.0%)	3	(0.4%)	0	(0.0%)	1	(0.1%)
**Worm expulsion**	1	(0.1%)	25	(3.3%)	6	(0.8%)	2	(0.3%)
**Haematuria**	28	(3.7%)	4	(0.5%)	0	(0.0%)	3	(0.4%)
**Adenopathy**	1	(0.1%)	1	(0.1%)	0	(0.0%)	0	(0.0%)
**Palpitation/tachycardia**	5	(0.7%)	4	(0.5%)	3	(0.4%)	4	(0.5%)
**Orthostatic hypotension**	0	(0.0%)	3	(0.4%)	0	(0.0%)	1	(0.1%)
**Other**	145	(19.2%)	100	(13.3%)	57	(7.5%)	194	(25.8%)

However, there were more subjects with pre-existing complaints classified as “Other”: 496 subjects (202 on standard treatment, 294 on coadministered treatment). The frequency of these varied widely between the villages: 57 subjects (7.5%) in Bougoula (Kolondièba, standard treatment), 100 subjects (13.3%) in Ména (Bougouni, coadministered treatment), 145 subjects (19.2%) in Tienkoungoba (Bougouni, standard treatment) and 194 subjects (25.8%) in Kebila (Kolondièba, coadministered treatment). The most commonly reported were cough (all villages except for Bougoula), lower back pain (Kolondièba, both villages), epigastralgia (Bougouni, both villages) and various other types of pain.

### Adverse Events

**Figure 3:**
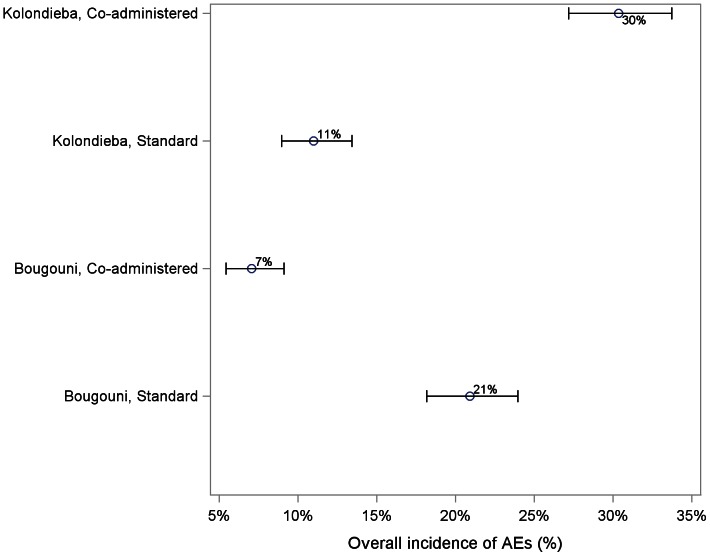
Overall incidences of adverse events. Graph shows the overall incidence of adverse events in each village, calculated as the percentage of subjects in each village who reported at least one adverse event at any time after treatment, with associated 95% confidence intervals.

Table 4 summarizes the overall incidence of AE. The incidence of events in each village with its 95% confidence interval is also summarized in Figure 3.

**Table 4:** Adverse event summary for the population according to district and d treatment.

	BOUGOUNI				KOLONDIÈBA			
	Standard		Co-administered		Standard		Co-administered	
	n	(%)	n	(%)	n	(%)	n	(%)
**Any AEs**								
**No**	597	(79.1%)	697	(92.9%)	672	(89.0%)	523	(69.6%)
**Yes**	158	(20.9%)	53	(7.1%)	83	(11.0%)	228	(30.4%)
**Any AEs (excluding exacerbated complaints)**								
**None**	598	(79.2%)	697	(92.9%)	673	(89.1%)	523	(69.6%)
**1 or more**	157	(20.8%)	53	(7.1%)	82	(10.9%)	228	(30.4%)
**Any exacerbated complaints**								
**None**	746	(98.8%)	748	(99.7%)	749	(99.2%)	746	(99.3%)
**1 or more**	9	(1.2%)	2	(0.3%)	6	(0.8%)	5	(0.7%)
**Maximal severity** 1								
**None**	598	(79.2%)	697	(92.9%)	673	(89.1%)	523	(69.6%)
**Minor**	90	(11.9%)	20	(2.7%)	47	(6.2%)	197	(26.2%)
**Moderate**	61	(8.1%)	32	(4.3%)	33	(4.4%)	28	(3.7%)
**Major**	6	(0.8%)	1	(0.1%)	2	(0.3%)	3	(0.4%)
**No. of AEs recorded**								
**None**	597	(79.1%)	697	(92.9%)	672	(89.0%)	523	(69.6%)
**1**	105	(13.9%)	29	(3.9%)	55	(7.3%)	168	(22.4%)
**2**	41	(5.4%)	19	(2.5%)	18	(2.4%)	47	(6.3%)
**3 or more**	12	(1.6%)	5	(0.7%)	10	(1.3%)	13	(1.7%)

1 Calculated for each subject as maximal severity of all events reported; thus data is not available where exacerbated complaints were not recorded as AEs.

The confidence intervals are all narrow due to the large sample size in each village, and there is no overlap between the four. The difference between treatment regimens differs between the districts, with a higher incidence for the coadministered treatment than the standard treatment in Kolondièba (30.4% vs. 11.0%), but a lower incidence in Bougouni (7.1% vs. 20.9%). Due to the clear differences between the incidences of AE in each village, formal statistical comparison between the two treatments is not appropriate. The intra-class correlation coefficient (ICC) was estimated to be 0.069 (95% CI: 0 to 0.174).

No serious adverse events were reported following treatment. Twelve subjects (8 out of 241 with any AE on standard treatment and 4 out of 281 on coadministered treatment) reported events of major severity. Where necessary, subjects were treated symptomatically, most requiring paracetamol for pain or headache.

Most subjects only experienced one type of AE: 66.4% (160/241) of subjects reported an AE with the standard treatment and 70.1% (197/281) with the coadministered treatment. Fewer than 2% of all subjects in each village reported 3 or more events. Of those subjects who did report an AE, 7.6% and 12.0% in the control villages and 9.4% and 5.7% in the coadministration villages reported 3 or more. One subject (Bougouni, standard treatment) had seven types of AE recorded.

Overall, there was no difference in AE rates according to whether there were pre-existing complaints reported.

In most cases, pre-existing complaints had either disappeared or improved by Day 8. Some were ongoing, and a small number still continued up to Day 15. At the Day 8 assessment, a small number of complaints were reported as worse after treatment: these were mainly headaches (7 subjects) and abdominal pain (6 subjects). There was also one case each of fever, joint pain and deafness, which were exacerbated by treatment. At the final (Day 15) assessment, only one subject reported an exacerbation of a pre-existing complaint. This was a case of abdominal pain in the standard treatment (Kolondièba).

All but one of the headaches exacerbated by treatment were reported as AEs. Of the six subjects with abdominal pain, four were reported as an AE, two were not. The case of worsened fever was not reported as an AE, nor was the exacerbated deafness. There was one case of exacerbated joint pain that was reported as an AE.

**Figure 4:**
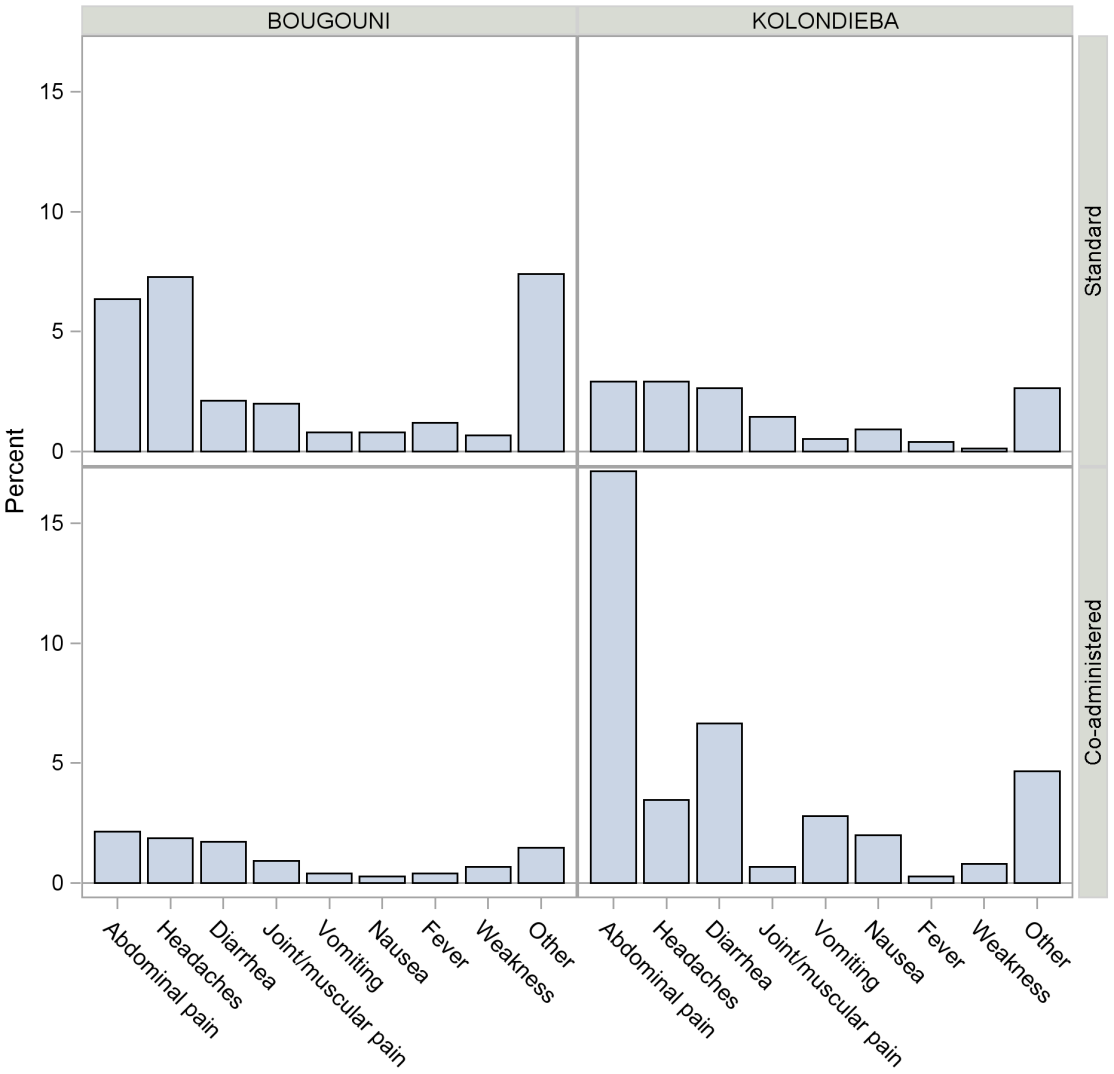
Incidence of most common adverse events. Graph shows the incidence of the most commonly reported types of event overall. Percentages are calculated using the total number of treated subjects in each village. “Other” comprises any events not listed in the Case Report Form.

**Figure 5:**
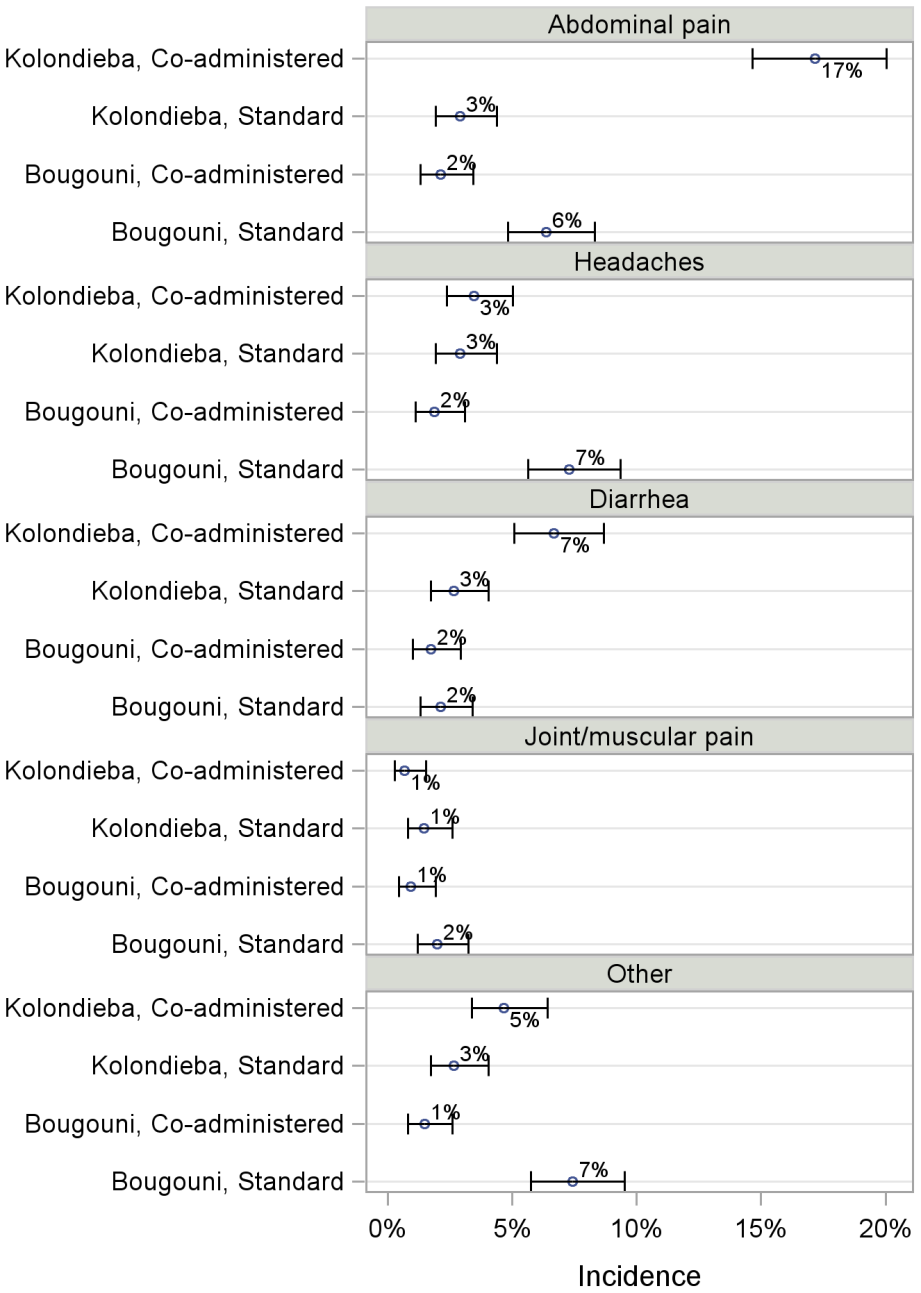
Most common adverse events. Graph shows the incidence of the most commonly reported types of event overall, with 95% confidence intervals. Percentages are calculated using the total number of treated subjects in each village. “Other” comprises any events not listed in the Case Report Form.

Table 5 shows the frequency of each individual type of event as listed in the CRF. The data is summarized graphically for the most frequent events in Figure 4. Abdominal pain, headaches, diarrhea and events other than those itemized in the CRF were the most common types of event in all villages. The incidence of each of the main events with 95% confidence intervals is summarized for each village in Figure 5.

**Table 5:** Type of adverse event encountered according to district and treatment received.

	BOUGOUNI				KOLONDIÈBA			
	Standard		Co-administered		Standard		Co-administered	
	n	(%)	n	(%)	n	(%)	n	(%)
**Abdominal pain**	48	(6.4%)	16	(2.1%)	22	(2.9%)	129	(17.2%)
**Headaches**	55	(7.3%)	14	(1.9%)	22	(2.9%)	26	(3.5%)
**Diarrhea**	16	(2.1%)	13	(1.7%)	20	(2.6%)	50	(6.7%)
**Joint/muscular pain**	15	(2.0%)	7	(0.9%)	11	(1.5%)	5	(0.7%)
**Vomiting**	6	(0.8%)	3	(0.4%)	4	(0.5%)	21	(2.8%)
**Nausea**	6	(0.8%)	2	(0.3%)	7	(0.9%)	15	(2.0%)
**Fever**	9	(1.2%)	3	(0.4%)	3	(0.4%)	2	(0.3%)
**Weakness**	5	(0.7%)	5	(0.7%)	1	(0.1%)	6	(0.8%)
**Constipation**	4	(0.5%)	3	(0.4%)	2	(0.3%)	2	(0.3%)
**Flatulence/dyspepsia**	2	(0.3%)	4	(0.5%)	2	(0.3%)	2	(0.3%)
**Swelling of eyelids/abnormal feeling in eyes**	0		1	(0.1%)	4	(0.5%)	2	(0.3%)
**Fatigue**	1	(0.1%)	1	(0.1%)	1	(0.1%)	3	(0.4%)
**Tinnitus**	3	(0.4%)	0		1	(0.1%)	0	
**Palpitation/tachycardia**	1	(0.1%)	1	(0.1%)	0		1	(0.1%)
**Haematuria**	0		2	(0.3%)	0		0	
**Rash/plaque**	0		0		0		2	(0.3%)
**Swelling of (upper/lower) limbs**	0		0		1	(0.1%)	1	(0.1%)
**Adenopathy**	0		0		1	(0.1%)	0	
**Deafness**	0		0		1	(0.1%)	0	
**Orthostatic hypotension**	0		0		1	(0.1%)	0	
**Scrotal reaction**	0		0		0		1	(0.1%)
**Worm expulsion**	1	(0.1%)	0		0		0	
**Other**	56	(7.4%)	11	(1.5%)	20	(2.6%)	35	(4.7%)

Percentages for incidence of each event are calculated using the numberof treated subjects in each village.

Overall, and in three of the four villages, the most frequently reported event was abdominal pain, with rates ranging from 2.1% (Bougouni, coadministered treatment) to 17.2% (Kolondièba, coadministered treatment) which was considerably higher than in any of the other three villages (Figures 4 and 5). The exception was Tienkoungoba (Bougouni, standard treatment), where more people experienced headaches (55 subjects, 7.3%) or other types of event (56 subjects, 7.4%). The incidence of diarrhea was slightly higher in the coadministered treatment village in Kolondièba than in the other three villages (6.7% compared to rates below 3%). The incidence of joint or muscular pain was similar in all four villages.

The only other events reported by more than 1% of the study population in any of the villages were vomiting (2.8% Kolondièba, coadministered treatment), nausea (2.0% Kolondièba, coadministered treatment) and fever (1.2%, Bougouni, standard treatment). Of these, the only event for which the upper limit of the confidence interval exceeded 4% was vomiting (Kolondièba, coadministered treatment, upper 95% confidence limit = 4.2%).

The upper 95% confidence limit for the prevalence of events specified in the CRF but which were not experienced by any subjects in the study is 0.5%. With the exception of nausea and vomiting, the incidences of all other AE types specified in the CRF are comparable between the villages, with overlapping confidence intervals.

Of the five subjects who had pre-existing complaints exacerbated which were not recorded as AE, two reported worsened abdominal pain, and one each reported heightened fever, headache and exacerbated deafness.

Of the 12 subjects with AE classified as major, 8 received standard treatment (6 in Bougouni district, 2 in Kolondièba) and 4 received coadministered treatment (1 in Bougouni district, 3 in Kolondièba). The distribution of events and their nature is shown in Table 6.

**Table 6:** Major adverse events reported post treatment.

	BOUGOUNI		KOLONDIÈBA	
	Standard	Co-administration	Standard	Co-administration
	n	n	n	n
**NUMBER**	6	1	2	3
**Otalgia**	1^1^			
**Otorrhea**	1			
**Thoracis pain/vertigo**			1	
**Swollen eyelids**				2^2^
**Weakness**		1		
**Abdominal Pain**	1			
**Headaches**	1			
**Diarrhea**	1			1
**Worm Expulsion**	1			
**Joint/muscle pain**			1	

1 Exacerbation of pre-existing complaint.

2 Onset occurred on Day 7 for one subject and Day1 for the other. Neither subject had any abnormal findings from the eye examination, although one subject did report “Watering (larmoiement)” before treatment.

There was no clear pattern to the types of event reported as “Other”. In Tienkoungoba (standard treatment, Bougouni), the most common events were respiratory in nature (18 cases, comprising coughs and rhinitis), and vertigo (15 cases). In Bougoula (standard treatment, Kolondièba) where the event rate was lower, vertigo was the most commonly reported “Other” event (6 cases). In Kebila (coadministered treatment, Kolondièba), the most common events were somnolence (26 cases) and vertigo (9 cases). The most frequent type of “Other” event in Mena (coadministered treatment, Bougouni) was epigastralgia, reported by 3 subjects.

**Figure 6:**
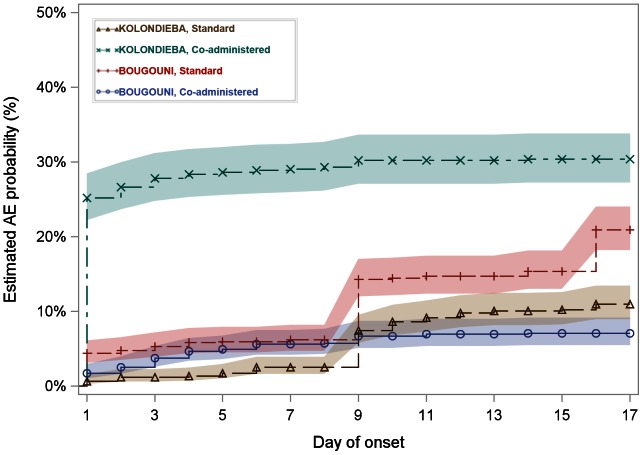
Time to onset of adverse events. The graph shows the estimated proportion of treated subjects in each village on each study day who had reported at least one adverse event from the time of receiving the study medication. The band represents the 95% confidence region for the estimates. Estimates were derived using survival analysis methods.

There is a clear difference between the villages in time to onset of events, with most of the events occurring immediately after treatment in Kebila (Kolondièba, coadministered treatment, Figure 6). There is also evidence that the event rate increased on day 8 with the administration of azithromycin in the standard treatment villages.

The median time to the onset of the first event, of any type, calculated from all those with any event, is 8 days for the two standard treatment villages compared to 2 days and 0 days for the coadministered treatment villages in Bougouni and Kolondièba respectively.

For the five most common types of event, namely, headaches, abdominal pain, diarrhea, joint/muscular pain and other non-specified events, a similar pattern in terms of time to onset was seen. This was most marked for abdominal pain: in Kebila (Kolondièba, coadministered treatment), almost all cases of abdominal pain first occurred on the day of drug administration. For diarrhea and events not specified in CRF, however, the time to onset was most rapid in the Kolondièba coadministered treatment village. The median time to onset of events with the standard treatment was always Day 8, coinciding with the administration of azithromycin. It is clear from the graphs that onset was quickest with the coadministered treatment in Kolondièba, with most events being reported as starting on the day of treatment administration. Duration of adverse events (number of days from first to last reporting of an event) is summarized in Figure S2

## Discussion

The aim of the study was to demonstrate that the prevalence of AE after administering ivermectin, albendazole and azithromycin simultanously was similar to the standard regimen where azithromycin is given one week after the coadminstration of ivermectin and albendazole. A similar approach to the establishment of safety of three drug combinations was taken for albendazole/ivermectin/praziquantel with a pharmacokinetic study [24] followed by clinical safety evaluation [25]. The study was designed as a cluster randomized study, with two villages in each of two districts in Mali being randomized to one of the two regimens; all enrolled subjects in each village received the treatment as determined for that village. Since only four clusters (villages) were studied, it is difficult to determine whether the clear differences in AE between clusters (villages) were due to treatment or to other factors. This is a major limitation of the study that the villages as the clusters consitute the main units of analysis, a fact that was not appreciated when the study was designed. The high ICC (0.069) suggests that the study was under-powered and that ideally many more smaller clusters should have been studied; however, this would have imposed considerable logistical problems and may not have been feasible in the current setting. In retrospect, the study design chosen was inappropriate, and found to be underpowered, which emphasises the need for statistical input when considering alternative approaches.

No serious adverse events were reported, and adverse events that were reported were generally mild or moderate.

The uptake of treatment was good in all four villages and all subjects were followed-up for the requisite time. Administration of drugs was almost always after a meal, except for the coadministered treatment in Kebila (Kolondièba), where a quarter of subjects took their medication on an empty stomach. Incorrect dose by height was rare in all villages with errors made in less than 2% of subjects

There was a marked difference in AE rates seen between the villages. The event rate was 20% or above in the village allocated standard treatment in Bougouni and the village allocated coadministered treatment group in Kolondièba. Conversely, the rates in the other two villages were 11% or below and were close to the projected rates of 5% and 8% for standard treatment and coadministered treatment respectively. The difference could be partly attributable to the two villages with the higher AE rates being more developed with a better educated population who were more likely to report AE. Both of these villages were situated on a main road with better access to services and other infrastructure. The other two villages were less developed and in more rural locations. There did not appear to be any relationship between the incidence of AE and the presence of pre-existing complaints, since within each village, the prevalence of experiencing any AE was similar for those with and without complaints at baseline. Nevertheless, the two villages with the highest AE rates also had the highest prevalence of pre-existing complaints (44.1% and 46.9%). This provides further support to the underlying difference between the villages in terms of their attitude to health. Although a potential source of bias, there is no evidence that the differing frequencies of AEs was the result of treatment seeking behaviour within the communities.

The onset of events with coadministered treatment in Kolondièba was extremely rapid with nearly all events, the most frequent being abdominal pain (16.1%), occurring on the day of administration. The incidence of diarrhea was also higher than in other villages. All other events were reported by fewer than 5% of the study population in that village. Adverse events in the standard treatment villages generally occurred when azithromycin was given on Day 8. This was especially noticeable for gastrointestinal events such as abdominal pain and diarrhea.

All events other than the most common (abdominal pain, headaches, diarrhea, joint or muscular pain, and ‘other’ non-specified events) were seen in less than 1% of the study population in all villages. The exceptions were vomiting (2.8%) and nausea (2%) in the coadministered treatment village in Kolondièba and fever (1.2%) in the standard treatment village in Bougouni. Events described as “Other” were most commonly respiratory symptoms, vertigo, or somnolence. There was no clear pattern associated with treatment.

Although examinations were conducted to record the presence of symptoms and signs of trachoma and LF, no tests (antigen test or night blood for microfilaria) were conducted to demonstrate asymptomatic infection with lymphatic filariasis. Clearance of microfilaraemia following treatment with ivermectin and albendazole may lead to symptoms such as myalgia and joint pains, but the incidence of such symptoms in the treated population as a whole was low [8]. This, coupled with the relative rarity of clinical evidence (hydrocele or lymphoedema), suggests that the National Lymphatic Filariasis Elimination Programme has made excellent progress towards controlling the disease in the study areas as a result of 5 rounds of annual MDA prior to the study. In the case of trachoma, elimination of *Chlamydia trachomatis* in an individual would not be expected to cause AE. The lack of significant levels of clinical disease may, however, have led to a lower than expected AE rate, especially with respect to those events typically associated with treatment of microfilaraemia.

Another limitation of the study is that treatment allocation was not blinded to the investigators, potentially resulting in observer bias in the assessment of AE. Furthermore, subjects were aware of their treatment regimen. As part of the study protocol, the cost of treating AE was met by the sponsor, which may also have influenced subjects to over-report events in order to access medical services. The analysis took no account of family or household groupings, which may also have a bearing on AE reporting. However, given the limitations imposed by the design, and the large discrepancy between AE rates in the four villages, this was not felt to be a major omission.

While the study design does not permit a statistical conclusion to be made, the pattern and timing of events does suggest that most AE are associated with administration of azithromycin, and that coadministration of all three drugs does not increase the frequency or severity of AE in this setting.

### Conclusions

The design of the study with a small number of large clusters means that comparisons of the overall AE rates for the two treatment regimens are difficult to make since there were only two villages receiving each treatment, and the AE rates differed markedly between villages. Nevertheless, there is no clear evidence to suggest that the coadministered treatment is associated with a higher rate of AE than the standard treatment. The types of event occurring most frequently were similar for all four villages, and the events reported, especially abdominal pain and diarrhoea were temporally accociated with azithomycin administration as expected. The study demonstrated that it is feasible to administer the three drugs together, which would significantly reduce the logistical demands on conducting mass treatment of LF and trachoma in this setting.

However, given the limitations of the current study, further investigation would be desirable. Any new study will need to consider the design issues raised here, and especially whether a cluster randomised approach is appropriate. Furthermore, the studies need to be conducted in areas where there is an appreciable prevalence of LF to determine whether coadministered treatment increases the incidence of AEs associated with microfilaraemia.

## Supporting Information

Checklist S1 **Consort Checklist.**
(DOC)

Figure S1 **Map of Mali and of Sikasso district showing position of study villages and roads in relation to the regional capital.**
(TIF)

Figure S2 **Overall duration of adverse events.** The overall duration is calculated as the time from the first day when any events were reported to the last day when any events were reported. The diamond represents the mean duration with the horizontal bar in the box showing the median. The box contains the inter-quartile range (IQR), with whiskers extending to 1.5 times the IQR. Values outside that range are depicted as circles. The median number of days (depicted by the left-most vertical bar in the boxes in the plots) was 5 days or less for all villages. The means (depicted by the diamonds) are somewhat higher due to the skewdness of the data. The unspecified (Other) events were of generally longer duration than those specified in the Case Record Form. Abdominal pain tended to be of shortest duration. The events in the standard treatment village in Bougouni lasted mostly for one day only.
(TIFF)

Text S1 **Study Protocol.** Final version of protocol entitled ‘A pharmacovigilance study on the safety of integrated treatment of Trachoma and Lymphatic Filariasis in children and adults living in the Sikasso region of Mali’ dated 8^th^ August 2009. This protocol was produced in both French and English.
(DOC)

Text S2 **Ethics Approval.** Approval (in French) of Version 2 of protocol issued on 5^th^ October 2009 by the Ethics Committee of the Faculty of Medicine, Pharmacy and Dentistry of the University of Bamako, Mali.
(PDF)
