# Supplementary figures and images for: A Cluster Randomized Study of The Safety of Integrated Treatment of Trachoma and Lymphatic Filariasis in Children and Adults in Sikasso, Mali
Source: PLoS Negl Trop Dis. 2013 May 9;7(5):e2221. doi: 10.1371/journal.pntd.0002221 (PMC3649960; doi:10.1371/journal.pntd.0002221)

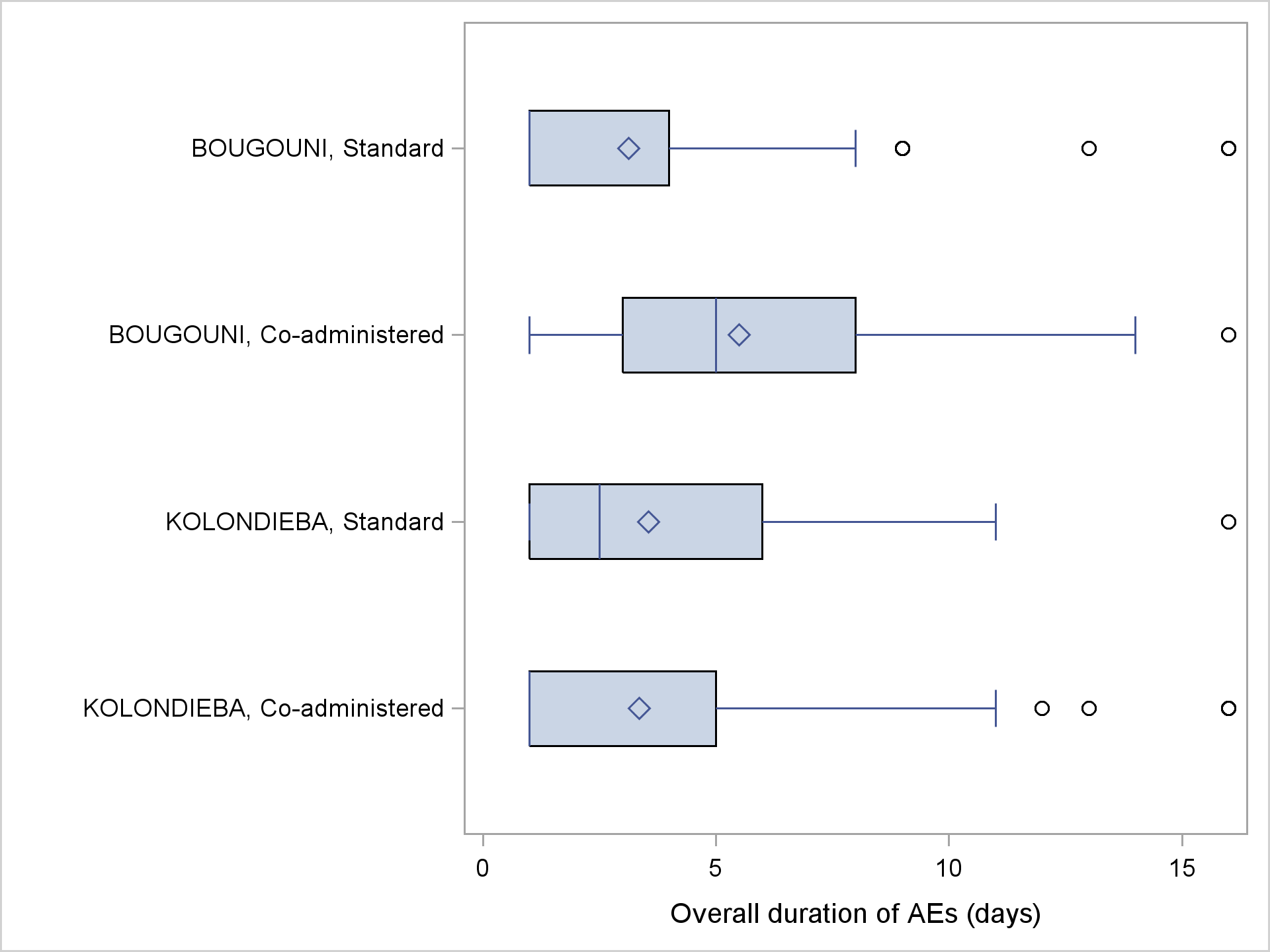

Supplement: Figure S1 — Map of Mali and of Sikasso district showing position of study villages and roads in relation to the regional capital. (TIF) [file pntd.0002221.s002.tif]

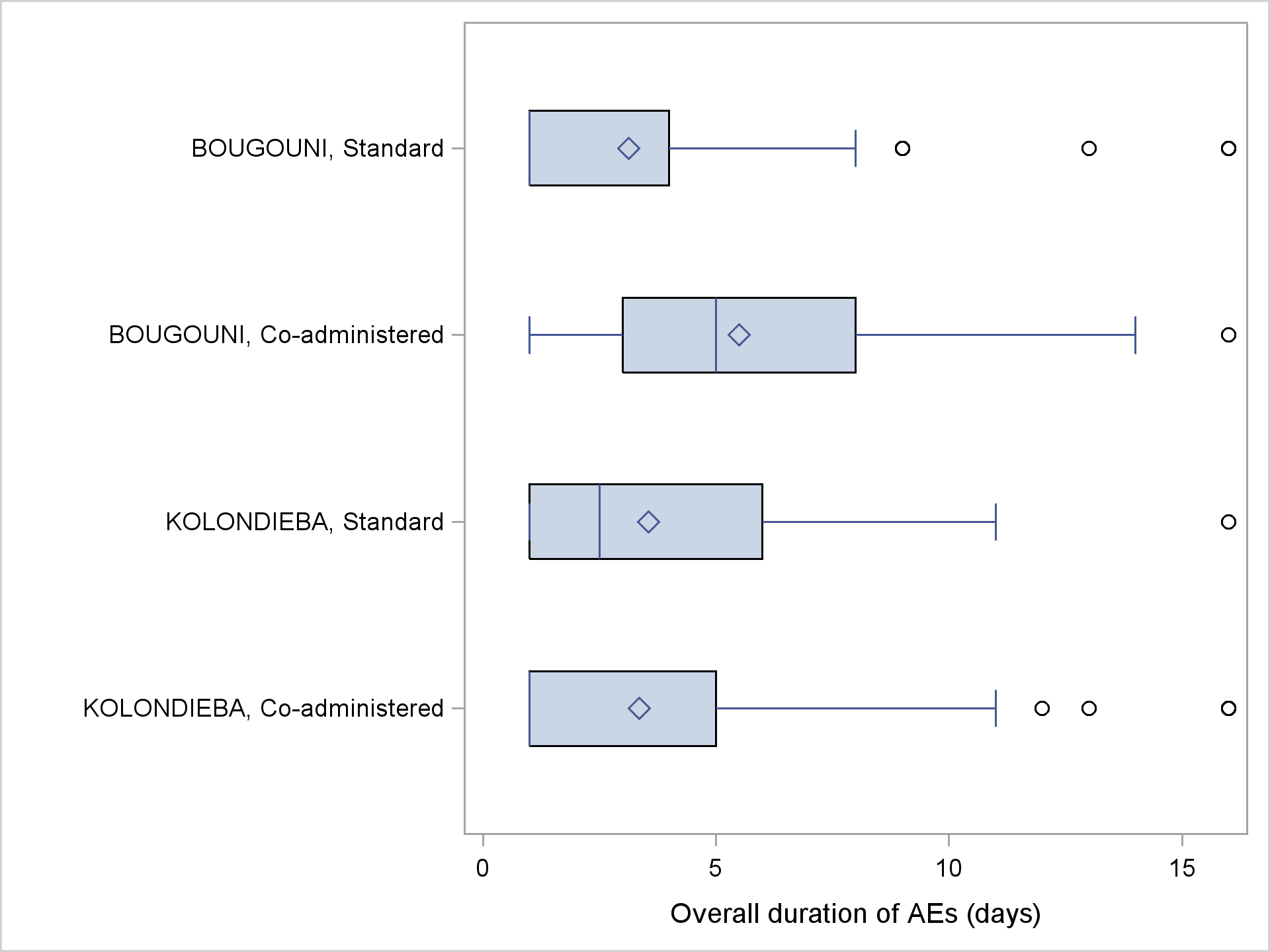

Supplement: Figure S2 — Overall duration of adverse events. The overall duration is calculated as the time from the first day when any events were reported to the last day when any events were reported. The diamond represents the mean duration with the horizontal bar in the box showing the median. The box contains the inter-quartile range (IQR), with whiskers extending to 1.5 times the IQR. Values outside that range are depicted as circles. The median number of days (depicted by the left-most vertical bar in the boxes in the plots) was 5 days or less for all villages. The means (depicted by the diamonds) are somewhat higher due to the skewdness of the data. The unspecified (Other) events were of generally longer duration than those specified in the Case Record Form. Abdominal pain tended to be of shortest duration. The events in the standard treatment village in Bougouni lasted mostly for one day only. (TIFF) [file pntd.0002221.s003.tiff]
