# Supplementary material for: A Cluster Randomized Study of The Safety of Integrated Treatment of Trachoma and Lymphatic Filariasis in Children and Adults in Sikasso, Mali
Source: PLoS Negl Trop Dis. 2013 May 9;7(5):e2221. doi: 10.1371/journal.pntd.0002221 (PMC3649960; doi:10.1371/journal.pntd.0002221)
Supplement: Text S1 — Study Protocol. Final version of protocol entitled ‘A pharmacovigilance study on the safety of integrated treatment of Trachoma and Lymphatic Filariasis in children and adults living in the Sikasso region of Mali’ dated 8th August 2009. This protocol was produced in both French and English. (DOC) [file pntd.0002221.s004.doc]

| **RÉPUBLIQUE DU MALI**  **UN PEUPLE UN BUT UNE FOI** | | **COOPÉRATION INTERNATIONALE** |
| --- | --- | --- |
| **ministÈre de la santÉ du mali**  **----------------------------** | **Ministère deS enseignementS SECONDAIRE supÉrieur et de la rEcherche scientifique** | **International trachoma initiative** |
| **DNS/CVD/CNAM** | **FMPOS** | **ITI-MALI** |

**RESEARCH PROTOCOL**

# A PHARMACOVIGILANCE STUDY ON THE SAFETY OF INTEGRATED TREATMENT OF TRACHOMA AND LYMPHATIC FILARIASIS IN CHILDREN AND ADULTS LIVING IN THE SIKASSO REGION OF MALI

**Final Version**


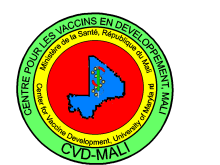

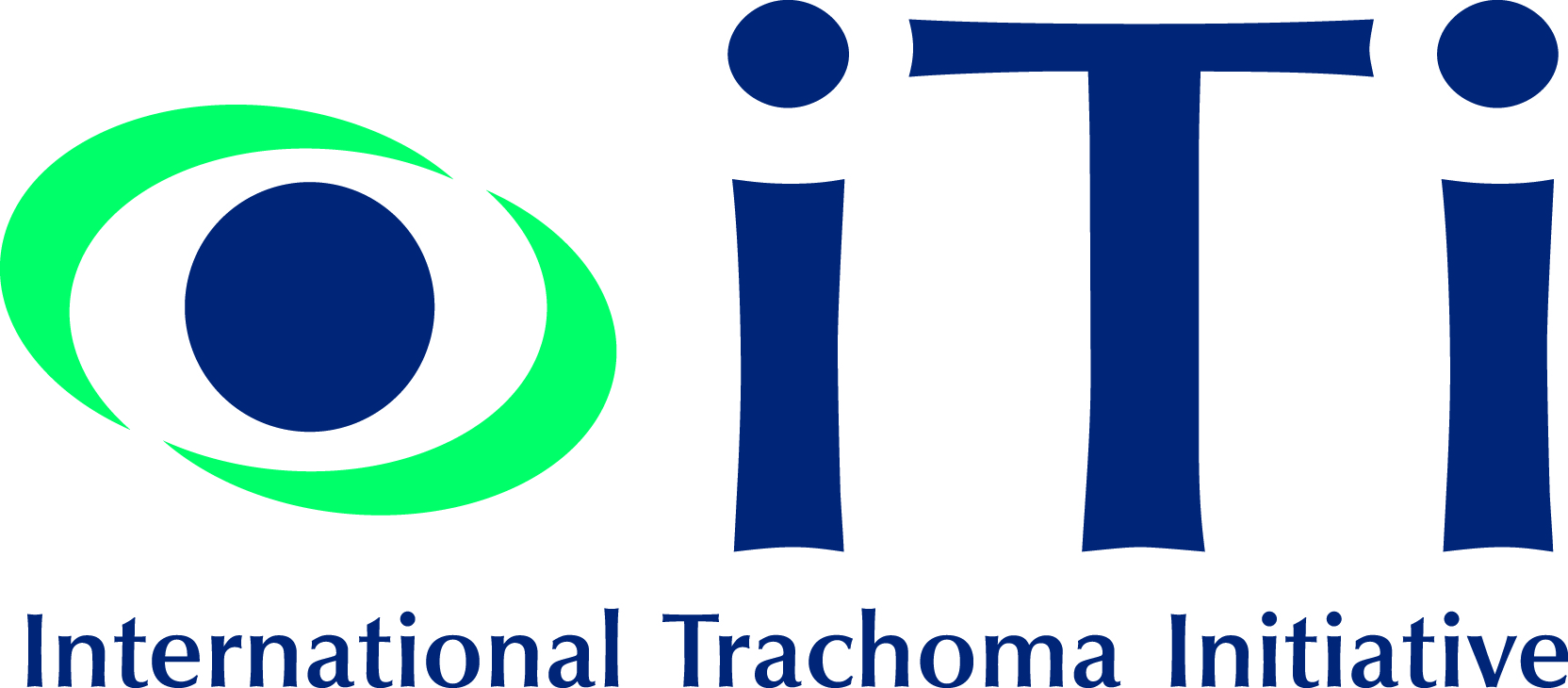


**PRINCIPAL INVESTIGATORS:**

1. Pr Samba SOW: Head of CVD-Mali, Principal Investigator.
2. Dr Moussa Hama Sankaré: Research Coordinator/International Trachoma Initiative/Mali
3. Dr Yaya Coulibaly: Head of Filariasis Department/MRTC and CNAM Research Scientist
4. Dr Mahamadou Kéita, Medical Researcher, CVD-Mali
5. Dr Sory Ibrahima Bamba, Head of the Blindness Prevention and Control Division
6. Pr Mamadou Souncalo Traoré, Head of the Public Health Research Department, FMPOS
7. Dr Fadima Cheick Haïdara, Medical Researcher, CVD-Mali

TABLE OF CONTENTS

| **Executive Summary** | **4** |
| --- | --- |
| **1. Introduction** | **5** |
| 1.1 Background | 5 |
| 1.2 Description of Drugs | 5 |
| 1.3 Safety Data | 7 |
| 1.4 Study Rationale | 9 |
| 1.5 Assumption | 9 |
| **2. Objectives** | **10** |
| 2.1 General Objective | 10 |
| 2.2 Specific Objectives | 10 |
| **3. Methodology** | **11** |
| 3.1 Study Type | 11 |
| 3.2 Study Sites | 11 |
| 3.3 Selection Criteria | 11 |
| 3.4 Investigational Treatment and Surveillance | 15 |
| 3.5 Sampling | 16 |
| 3.6 Randomization Criteria | 17 |
| 3.7 Awareness-raising | 18 |
| 3.8 Study Process | 19 |
| 3.9 Data Collection Tools | 20 |
| 3.10 Data Collection Methods | 21 |
| 3.11 Investigator's Guide | 22 |
| **4. Operational Definitions** | **24** |
| **5. Event Assessment** | **26** |
| 5.1 Severity Assessment | 26 |
| 5.2 Causality Assessment | 26 |
| 5.3 Safety and Feasibility Evaluation of the Study Triple Therapy | 26 |
| **6. Data Analysis** | **27** |
| 6.1 Data Acquisition and Presentation | 27 |
| 6.2 Primary Analysis | 27 |
| 6.3 Comparative Analysis | 27 |
| 6.4 Adverse Events | 27 |
| 6.5 Demographic and Baseline Characteristics | 27 |
| 6.6 Medical History | 27 |
| 6.7 Medical and Non Medical Treatments | 27 |
| **7. Ethical Considerations** | **29** |
| 7.1 Study Risks | 29 |
| 7.2 Study Benefits | 29 |
| **8. Presentation of the Study Report** | **31** |
| **9. Resources** | **32** |
| 9.1 Human Resources | 32 |
| 9.2 Material Resources | 34 |
| 9.3 Financial Resources & Budget | 35 |
| **10. Time Diagram** | **36** |
| **11. Bibliographical References** | **38** |
| **12. Appendices** | **40** |

**Addresses of researchers**

(principal investigator, co-investigators, research associates)

| **First name, last name and title** | **Address** | **Percentage**  **of Time Spent** |
| --- | --- | --- |
| ***Principal Investigator:***  Pr Samba SOW,  Head of CVD-Mali | CVD-Mali, Ministère de la Santé, Ex-Institut Marchoux, BP 251, Bamako, Mali/CNAM quartier Djikoroni Para, Bamako  Office Tel. +223 20 23 60 31  Fax: + 223 20 22 28 83  Email: [ssow@medecine.umaryland.edu](mailto:ssow@medecine.umaryland.edu) | 20 % |
| ***Co-investigator:***  Dr Moussa Hama Sankaré | ITI Mali Research Coordinator  Hamdallaye ACI 2000 ; Bamako  OfficeTel.: (+223) 229 61 11  Fax: (+223) 229 32 01  Tel GSM: 66 95 53 60  Email: [itimaligao@afribonemali.net](mailto:itimalisearch@afribonemali.net) | 40 % |
| ***Co-investigator:***  Dr Yaya Coulibaly | Head of Filariasis Department/MRTC  CNAM/MRTC, Bamako  Tel GSM: (+223) 66 74 23 41  Email: [yicoulibaly@mrtcbko.org](mailto:yicoulibaly@mrtcbko.org) | 20 % |
| ***Co-investigator:***  Dr Sory Ibrahima BAMBA, Head of the Blindness Prevention and Control Division (DPLM/DNS) | Direction Nationale de la Santé  Bamako (Mali)  Home: Moribabougou  Tel: (+223) 222 64 97/917 81 42  E-mail: [sibamba@dnsmali.org](mailto:sibamba@dnsmali.org) | 10 % |
| ***Co-investigator:***  Pr Mamadou Souncalo TRAORE  Head of the Public Health Research Department at FMPOS | Faculté de Médecine, de Pharmacie et d’Odonto-Stomatologie  Tel: (+223) 222 52 77/GSM: 675 90 51  Fax: (+223) 222 96 58 ;  Bamako: Faladié SEMA Rue 881 Porte 69.  Tel: (+223) 220 6868 ;  E-mail: [traorem@afribonemali.net](mailto:traorem@afribonemali.net) | 10 % |
| ***Co-investigator:***  Dr Mahamadou Keita  Medical Researcher | CVD-Mali, BP 251, Quartier Djikoroni Para, Bamako  Office GSM: (+223) 66 71 45 37  Email: [mkeita@medicine.umaryland.edu](mailto:mkeita@medicine.umaryland.edu) | 20 % |
| ***Co-investigator:***  Dr Fadima Cheick Haïdara | CVD -Mali, BP 251, Bamako  Tel GSM: (+223 ) 66 73 34 91  E-mail: [fadimacheic@yahoo.fr](mailto:fadimacheic@yahoo.fr) | 10% |

**EXECUTIVE SUMMARY**

Infectious diseases, such as trachoma, lymphatic filariasis (LF) and onchocerciasis continue to be problematic in various regions of Africa and Asia. Disease elimination programs exist for each of these diseases. However, it is both costly and inefficient to have different groups of health care workers treat these diseases separately. An integrated delivery of disease elimination programs that maximizes operational efficiency in endemic areas is needed. One possible integrated disease elimination strategy would be based on mass administration of a 3-drug combination: ivermectin and albendazole for lymphatic filariasis, and azithromycin for trachoma. We propose an open-label community based randomized triple co-administration study of azithromycin, ivermectin and albendazole for children and adults living in the Sikasso region of Mali.

The primary objective of the study is to establish the safety and feasibility of integrated mass treatment of trachoma and LF using azithromycin in combination with albendazole and ivermectin. The secondary objective is to evaluate the outcome of the integrated mass treatment. The endpoints of the study are the incidence of adverse events reported after initiation of therapy, as well as safety data observed during the study period following therapy initiation.

For the study design, two groups of 2 villages will be chosen: 2 Villages A and 2 Villages B. Eligible individuals in Villages A of 90 cm or more will receive the standard treatment of ivermectin based on height (12 mg max) and 400 mg of albendazole per subject. One week later, the same subjects will receive azithromycin based on height (1 g max). Individuals in Villages B will receive the triple therapy using the three above-mentioned drugs at the same dosages. A double-sided height pole specifically graduated for azithromycin tablets and ivermectin will be used for determining doses based on height.

For this study, data will be summarized using descriptive statistics. Categorical variables will be summarized using percentages. For each continuous variable, the mean, median and standard deviation will be computed. The analysis will be based on all subjects who received at least one dose of study drug by comparing the frequency of adverse events in groups A and B.

After review and validation of the results from this study by a Data Safety Monitoring Board (DSMB) and the *Direction Nationale de la Santé*, district wide co-administration under the oversight of the Malian Ministry of Health could be considered.

# 1. INTRODUCTION

**1.1 Background**

While the separate treatments of trachoma and lymphatic filariasis are well documented, their integrated treatment still represents a global challenge. Azithromycin has been employed for over 10 years in trachoma control programs. The combination of ivermectin and albendazole is also considered a standard for the treatment of LF and has been shown to reduce transmission over several rounds of mass drug distribution (Ottesen, 1999).

Several studies reported on the safety of azithromycin taken with the ivermectin/albendazole combination.

A community study performed in Zanzibar by Mohammed KA *et al.* (2008) on the interaction of three drugs (praziquantel, ivermectin and albendazole) showed that 10 % (N=5,055) of treated subjects experienced mild adverse events, the most frequent being dizziness resolving within 24 hours of onset. The vast majority of adverse events (87.3%) occurred within 24 hours.

Another pharmacokinetic study on the interaction of the three drugs (azithromycin, ivermectin and albendazole) performed by Amsden GW *et al.* (2007) in the United States, on 18 healthy volunteers revealed that an increase of ivermectin doses did not result in an accompanying increase of adverse events, such as dizziness, typically associated with ivermectin. One subject reported mild indigestion following administration of the ivermectin/albendazole combination. The other subjects reported mild disequilibrium eight days after taking the three drugs together. None of these events required treatment.

Given the encouraging outcomes of this pharmacokinetic study, a pharmacovigilance protocol was initiated by the International Trachoma Initiative (ITI) to perform a pilot study of the adverse events associated with the co-administration of azithromycin, ivermectin and albendazole in the mass treatment of trachoma and lymphatic filariasis in children aged 5 (over 90 cm) and over and adults in the Sikasso region. It is an open-label, community–based, randomized study comparing a control group A which is to receive standard care (ivermectin + albendazole followed by azithromycin after 7 days) with a group B, which is to receive the investigational triple therapy. The study aims to assess the safety and feasibility of this triple mass therapy. The study outcomes could be used by Neglected Tropical Disease (NTD) control programs and for the scaling-up of the triple therapy at national or international levels in areas of coendemicity of LF and trachoma as well as in areas of triple endemicity of LF, onchocerciasis and trachoma. The study follows up the work on Zanzibar (Mohammed KA et al, 2008) which demonstrated the safety of a triple combination where schistosomiasis was coendemic with LF. In the Mali setting trachoma is the co target with LF and such as study is prerequisite to up scaling in many areas of West Africa. The Zanzibar study recommended that further studies be undertaken on triple therapy in areas which although treated had not been subject to 5 rounds of MDA and hence had potentially higher parasite loads than Zanzibar communities.

## 1.2 Description of Study Drugs

### *1.2.1 Azithromycin (Appendix H)*

Azithromycin is an azalide, a subclass of macrolides, for oral administration. It is quickly absorbed and peak plasma concentration is reached in 2 to 3 hours. Tissue levels of azithromycin are greater than plasma levels (which can be up to 50 times the maximum plasma concentration). The liver is the main biotransformation agent of azithromycin which is eliminated mainly by the bile and partly by urine. The plasma terminal half-life is 2-4 days.

It is indicated for patients with mild to moderate infections i.e. respiratory-tract infections; otitis media; skin and soft-tissue; infections; genital chlamydial infections; non-gonococcal urethritis etc…caused by susceptible strains of microorganisms (Pfizer, 2007).

The recommended dose for the mass treatment of trachoma is a single-dose of azithromycin 20mg/kg (Mabey & Bailey, 1999).

Azithromycin is available as 250 mg or 500 mg tablets and as 300 mg, 600 mg, 900 mg, or 1,200 mg bottles for oral suspension (Pfizer, 2007). In this study, 250mg tablets will only be given in single doses based on height as indicated in section 3.8.2 Triple Therapy Drugs and Administration.

### *1.2.2 Ivermectin (Appendix I)*

Ivermectin is an anthelminthic agent for oral administration. It is available in 3mg tablets. Ivermectin is indicated for the treatment of onchocerciasis, or river blindness caused by *Onchocerca volvulus* and strongyloidiasis caused by *Strongyloides stercoralis* (Merck, 1998).

Ivermectin is metabolized in the liver, and ivermectin and/or its metabolites are excreted almost exclusively in the feces over an estimated 12 days, with less than 1% of the administered dose excreted in the urine. The apparent plasma half-life of ivermectin is approximately at least 16 hours following oral administration.^10^

The recommended dosage for treatment of lymphatic filariasis is a single oral dose designed to provide approximately 150µg of ivermectin per kg of body weight. In this study, doses will be given based on height as indicated in section 3.8.2 Triple Therapy Drugs and Administration.

In international mass distribution campaigns, the most commonly used dose interval is 12 months. For the treatment of individual patients, retreatment may be considered at intervals as short as 3 months.

### *1.2.3 Albendazole (Appendix I)*

Albendazole is a benzimidazole carbamate. It is a broad-spectrum anthelminthic agent acting on nematodes, cestodes and some protozoa. Albendazole is orally administered. It appears to cause irreversible inhibition of glucose uptake by parasites. It is also active on larvae at the beginning of tissue migration (GlaxoSmithKline, 2003). It is indicated for the treatment of parenchymal neurocysticercosis due to active lesions caused by larval forms of the pork tapeworm, *Taenia solium*. It is also indicated for the treatment of cystic hydatid disease of the liver, lung, and peritoneum, caused by the larval form of the tapeworm, *Echinococcus granulosus* (GlaxoSmithKline, 2003).

Annual treatment with a single dose of 400 mg albendazole (or 15 mg/kg) in combination with ivermectin for lymphatic filariasis, a disease caused by *Wuchereria bancrofti (Wb)* and *Brugia malayi*, has been shown to reduce blood filarial counts and interrupt further transmission.

Following oral administration, the low proportion of absorbed albendazole (< 5 %) is metabolized to albendazole sulfoxide and sulfone. The plasma concentration in sulfoxide, which is the primary circulating active metabolite, is greatest about two and a half hour after administration. The plasma half-life of albendazole sulfoxide is 8 hours and 30 minutes. Albendazole sulfoxide and its metabolites seem to be mainly eliminated through the bile while urinary excretion would appear to be minimal (GlaxoSmithKline, 2003).

Albendazole is available as a 400 mg tablet. In young children, the tablets should be crushed or chewed and swallowed with a glass of drinking water. In this study, a 400 mg tablet will be given to all subjects.

1.3 Safety Summary

Multiple studies of the adverse events associated with albendazole, ivermectin and azithromycin administered together or separately have been carried out. Safety of the combination of albendazole, ivermectin and azithromycin delivered together is a key aspect that needs to be determined in this study.

***1.3.1 Safety of the co-administration of medications***

Two pharmacokinetic studies on the co-administration of ivermectin, albendazole, and praziquantel (Na-bangchang K *et al.,* 2006) and the co-administration of ivermectin, albendazole, and azithromycin (Amsden GW *et al.,* 2006) were carried out. A community study on the co-administration of ivermectin, albendazole, and praziquantel was carried out in Zanzibar (Mohamed KA *et al.,* 2008).

In a study examining co-administration of ivermectin, albendazole and praziquantel to over 5,000 children and adults in Zanzibar (Republic of Tanzania) as a pilot, prior to the wider administration of these drugs as a triple combination to over 700,000 individuals, both passive and active collection of adverse events were performed (Mohammed KA *et al.,* 2008). This study based its rationale on the fact that a prior study (Horton J *et al.,* 2000) showed that co-administration of albendazole and ivermectin proved extremely effective after single administration when used for treatment of lymphatic filariasis (LF) and had rare side-effects, other than those seen as therapeutic effects, which were usually due to dying parasites.

The more notable side effects, which are part of the individual’s inflammatory response to the dying parasites, are proportional to the parasitic load: the greater the load, the more frequent and severe are the reactions. They can range from systemic reactions such as headache, nausea, vomiting, abdominal discomfort, drowsiness, lightheadedness, myalgia, anorexia or malaise, to the more localized reactions of lymphadenitis, epididymitits, lymphangitis and abscess formation. It has been demonstrated that only in very heavily infected patients are these major or requiring more than symptomatic treatment (Loukas A & Hotez PJ, 2000).

Key to the rationale of the Zanzibar study was that pharmacokinetic data collected in healthy subjects by Na-bangchang K *et al.* in 2006 indicated no pharmacologic interactions among the three drugs. Co-administration of ivermectin, albendazole and praziquantel did not appear to increase their relative toxicities.

The Zanzibar study was conducted after five rounds of MDA for LF and enrolled individuals over 90 cm (5 years old and older), with those from 10 to 19 years representing the largest group. Only one individual reported to a health center to complain of vomiting. Active surveillance consisted of interviews with 5,055 persons between Days 5 and 7 after drug administration. Adverse experiences were reported by approximately 10% of those treated, with the vast majority (87.3%) occurring within 24 hours of triple drug administration. This decreased to 11.9% on the second day and 0.8% on the third day. Dizziness was the most commonly reported event and all symptoms were mild, resolving within 24 hours of onset. With general administration of the triple therapy (praziquantel, ivermectin, albendazole) to 700,000 subjects, active surveillance consisted of interviews with 19,043 persons. There were 266 (1.4%) individuals reporting side effects. All side effects were mild, transient and resolved within 24 hours. The most frequently reported was fatigue (Mohammed KA *et al.,* 2008).

***1.3.2 Safety of the administration of medications given separately***

Considered separately, each of the drugs in the triple drug co-administration (azithromycin, ivermectin, and albendazole) has well-documented associated adverse effects.

The safety and tolerability, as well as pharmacokinetics, of ivermectin, were examined by Guzzo *et al* (2002) in healthy volunteers in the United States. Of the 51 subjects receiving active drug, 12 (24%) reported at least one clinical adverse event, including six affecting the nervous system. Most commonly reported were headache, nausea, dizziness and exanthema.

In another investigational study conducted by Pfizer Inc. in 2007 and encompassing a median 87.5 days of therapy and 88 subjects, the reported events for azithromycin, given once at a dose of 1 gram, occurring in at least 1% of subjects include: diarrhea/loose stools (7%), nausea (5%), abdominal pain (5%), vomiting (2%), dyspepsia (1%) and vaginitis (1 %). Other events observed in this study in more than 5% of subjects include abdominal pain (14%), nausea (14%), vomiting (13%), diarrhea (12%), flatulence (5%), headache (5%) and abnormal vision (5 %). Of 88 subjects, 8 (9.1%) discontinued due to side effects considered related to study drug. Laboratory abnormalities seen during azithromycin administration include elevated serum creatinine phosphokinase, potassium, alanine aminotransferase (ALT), gamma-glutamyl transpeptidase and aspartate aminotransferase, occurring at a rate of 1-2 %.

Prescribing information for ivermectin lists pruritus and dizziness as the most frequently reported adverse effects (each at a rate of 2.8 %) when the drug is given in the range of 170 to 200 μg/kg for treatment of strongyloidiasis. Other reported adverse reactions occurring at rates of at least 1% include diarrhea (1.8%) and nausea (1.8 %). Laboratory abnormalities include elevation in ALT or aspartate aminotranferase (AST) (2%) and a decrease in leukocyte count (3 %). This data comes from a study of 109 patients.

It is expected that people with higher LF parasite loads will experience more severe Mazzotti reactions following treatment. This Mazzoti reaction is an expected and anticipated effect of treating patients with LF related to the death of the large number of parasites and the immunologic reactions to that event. Mazzotti reactions, described are: pruritus, skin involvement, including edema, popular and pustular or frank uriticarial exanthema, fever, tenderness or enlargement of inguinal, axillary and cervical lymph nodes and arthralgia/synovitis.

In a clinical study conducted by Mabey & Bailey (1999) on 963 subjects having received 100 to 200µg/kg of ivermectin, the most clinical complaints possibly, probably or definitely related to the study drug (> 1% of patients) include tachycardia (3.5%), peripheral edema (3.2%), facial edema (1.2%) and orthostatic hypotension (1.1%). The most common adverse experiences, regardless of causality were headache (22.3%) and myalgia (19.7 %). Ophthalmologic changes (limbitis) were observed primarily three days after treatment in a small proportion of adult patients. Safety of subjects aged 6 to 13 years reveals a similar profile to that observed in adults.

For albendazole, rates of adverse events depend on the disease being treated. Table 1 displays the adverse events rates (limited to those > 1%) for two diseases when treated with albendazole (GlaxoSmithKline, 2007). In rare cases, treatment with albendazole can reveal pre-existing neurocysticercosis and its co-administration with ivermectin can result in Steven Johnson syndrome.

**Table 1**: Adverse events linked to the treatment of hydatid disease and neurocysticercosis with albendazole

| **Adverse Events** | **Hydatid Disease (%)** | **Neurocysticercosis (%)** |
| --- | --- | --- |
| Abnormal liver function tests | 15.6 | < 1.0 |
| Abdominal Pain | 6.0 | 0 |
| Nausea/vomiting | 3.7 | 6.2 |
| Headache | 1.3 | 11.0 |
| Dizziness/Vertigo | 1.2 | < 1.0 |
| Increase intracranial pressure | 0 | 1.5 |
| Meningeal signs | 0 | 1.0 |
| Reversible alopecia | 1.6 | < 1.0 |
| Fever | 1.0 | 0 |

**1.4 Study Rationale**

Integrating the treatment of LF and trachoma would present logistic, health and economic advantages, helping to reduce the burden on an already-strained healthcare system.

Weighing the benefits and risks of implementing triple drug therapy in endemic areas should be done in the context of the known data and the perceived risk of extreme handicap faced by a substantial proportion of the population.

As described in extant literature, the major adverse events for azithromycin added to the ivermectin/albendazole combination that is already in use are gastrointestinal in nature and are not substantial (Pfizer 2007).

Azithromycin is also considered a standard for mass treatment of trachoma, a chronic follicular conjunctivitis caused by ocular strains of *Chlamydia trachomatis* that, although susceptible to several classes of antibacterials, remains the leading cause of preventable blindness worldwide (Pfizer 2007).

In Mali, 34.9% of children aged between 1 and 10 years old suffer from active trachoma, 2.5% of women aged 15 years old and over suffer from trichiasis (Strategic Plan for Trachoma Elimination 2005-2009) and 7.07% of the population suffer from lymphatic filariasis, i.e. more than 800,000 persons (Strategic Plan for Trachoma Elimination 2005-2009). More than 200,000 people are at risk of being handicapped by these two diseases if the prevalence of trachoma among children suffering from lymphatic filariasis is taken into account.

Given the significant impact and consequences of untreated LF and trachoma, the large populations at risk for both of these diseases and the resource constraints of the endemic areas, the potential to improve the lives of the patients, to optimize the available healthcare resources and to reach the maximum number of people make the risks of initiating this new triple drug administration acceptable.

However, before integration can occur, the safety of co-administration of the three drugs (azithromycin, ivermectin and albendazole) has to be taken into account.

**1.5 Assumption**

The incidence of adverse events associated with this triple drug administration would not be different to the incidence of those normally observed in standard treatments with ivermectin + albendazole and azithromycin.

**2. OBJECTIVES**

**2.1 General Objective**

Assess the safety and feasibility of integrated mass treatment using azithromycin in combination with albendazole and ivermectin.

**2.2 Specific Objectives**

1. Establish the incidence of adverse events associated with the mass triple drug administration;
2. Establish the incidence of serious adverse events associated with the mass triple drug administration.

**3. METHODOLOGY**

**3.1 Study Type**

This is an open-label, community–based, randomized, triple drug administration study of azithromycin, ivermectin and albendazole.

**3.2 Study Site**

***3.2.1 Mali***

The Republic of Mali is a landlocked nation in western Africa and the seventh largest country in Africa, with a population of approximately 12 million people. Globally, Mali is the world’s twenty-fourth largest country, comparable in size to South Africa or Peru (Loukas & Hotez, 2000).

***3.2.2 The Sikasso region***

*3.2.2.1 Presentation of the study region*

The Sikasso region covers 76,480 km^2^ and has a total population of 2,309,260 (2008). It has the highest population density of the country with 31 people/km^2^ in 2008 and a demographic growth rate of 2.4%. Located 380 km from Bamako, Mali’s capital city, the Sikasso region borders the Republic of Côte d’Ivoire to the south, Burkina Faso to the east, the Republic of Guinea-Conakry to the south-west and the Koulikoro region of Mali to the west. The region is composed of seven (7) “cercles” and eight (8) health districts: Bougouni, Kadiolo, Kolondieba, Koutiala, Sélingué, Sikasso, Yanfolila, and Yorosso. The Yanfolila “cercle” includes the Yanfolila and Sélingué health districts. The population lives in rural areas and is mainly made up of farmers and livestock farmers. Cotton farming is dominant and is the main source of income. The districts of Bougouni and Kolondieba, with a total population of 585,773, have been suggested as the project area.

**Figure 1:** Administrative Map of the Sikasso region

**Bougouni**

**Sikasso**

**KOULOKORO REGION**

**SEGOU REGION**

**Kadiolo**

**Kolondieba**

**Yorosso**

**Koutiala**

**Yanfolila**

**GUINEA-CONAKRY**

**COTE D’IVOIRE**

**BURKINA FASO**

*(Source: Communication de la Direction Régionale de la Santé de Sikasso, NTD Magazine, 2008)*

*3.2.2.2 Overview of Epidemiological Diseases*

The Sikasso region is located in the southern Sudan savannah where the climate is conducive to vector borne infections. The vectors encountered in the region are: mosquitoes for malaria and LF (*An. gambiae sl, An. funestus*), blackflies (*Simulium*) for onchocerciasis, sandflies (*Phlebotomus*) for leishmaniasis and midges of the Ceratopogonidae family for *Mansonella perstans*. Some villages are currently receiving treatment for ivermectin under a community directed approach as well as epidemiological surveillance for onchocerciasis. The treatment of some communities of the health districts of Sikasso, Bougouni, Kolondiéba, Yanfolila and Sélingué is underway. The prevalence of onchocerciasis is estimated at less than 0.05% in the Sikasso region. Schistosomiasis is mostly endemic in the area of the Sélingué dam / Yanfolila “cercle” with a prevalence of 27.4% for urinary schistosomiasis and 86% for intestinal schistosomiasis respectively. Soil-transmitted helminthiasis is endemic in all the districts of the region and is a public health concern, in particular ancylostomiasis with a prevalence within the Sikasso region ranging between 0 and 34.5% depending on villages (Communication de la Direction Régionale de la Santé de Sikasso, NTD magazine, 2008).

Trachoma is highly endemic in the Sikasso region and, according to preliminary results of the prevalence investigation conducted by ITI in June 2008, the prevalence of active trachoma is between 0.49% in Koutiala and 13.51% in Sélingué (*Table 2*). The prevalence in Bougouni and Kolondiéba is respectively 5.77% and 7.45%. These prevalence rates are lower than the 10% threshold recommended at the international level to justify mass treatment with azithromycin in a district. This should not, however, lessen the usefulness of the current study.

## *Overview of some research studies related to NTDs*

## The control interventions included in the 2004-2009 five-year Neglected Tropical Disease Control Plan and the implementation of some programme components, such as training, mass treatment, epidemiological surveillance, sentinel sites and *research,* will contribute to reducing the impact of these diseases. Since 2001, the United States National Institutes of Health (NIH) has conducted jointly with the Bamako Faculty of Medicine several research studies on NTDs and LF in particular.

- One of them, a study on the impact of the coendemicity of LF and *Mansonella perstans* in Mali as related to adverse events associated with the standard albendazole/ivermectin treatment in the Koulikoro region revealed that neither the frequency nor the severity of adverse events were increased in subjects co-infected with *W. bancrofti* and *M. perstans* (Keiser et al, 2003).

## A FMPOS study is currently assessing the efficacy and safety of albendazole and ivermectin administered twice yearly to *subjects living in endemic areas* in doses (albendazole 800 mg and ivermectin 330 µg/kg) that are double the standard annual dose of the National LF Elimination Programme. Preliminary results indicate an increased efficacy for the double dose given twice yearly (every 6 months) 12 months after the treatment. The frequency and intensity of adverse events encountered were similar in both groups.

- The pilot study of the impact of a single annual dose of albendazole + ivermectin in the Sikasso region also showed that LF transmission stopped after 5 rounds of annual mass treatment. The figures below show this impact on the transmission potential of mosquitoes (Figure 2) and on the level of human infection (Figure 3).

# Figure 2: Transmission potential of mosquitoes, impact of 5 mass treatment rounds


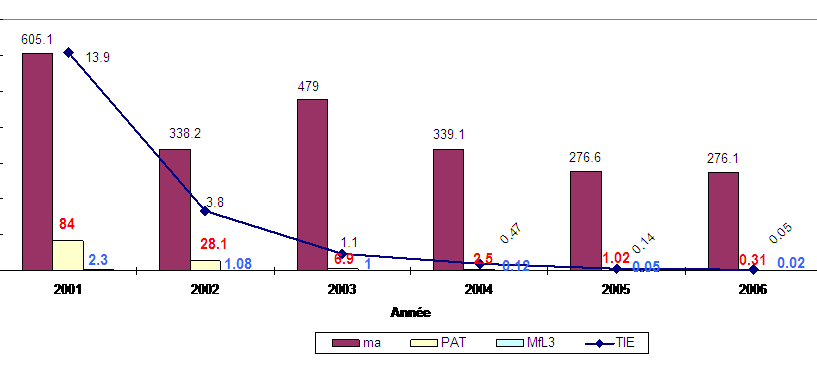


ma: Number of mosquito bites per person/per year

TIE: Insect inoculation rate (number of infective mosquito bites per person)

PAT: Annual transmission potential (cumulated TIE per year)

ML3: Average number of larvae at stage 3 per infective mosquito (more or less equal to 1 each year)

**Figure 3:** Human infection levels, impact of 5 mass treatment rounds

#
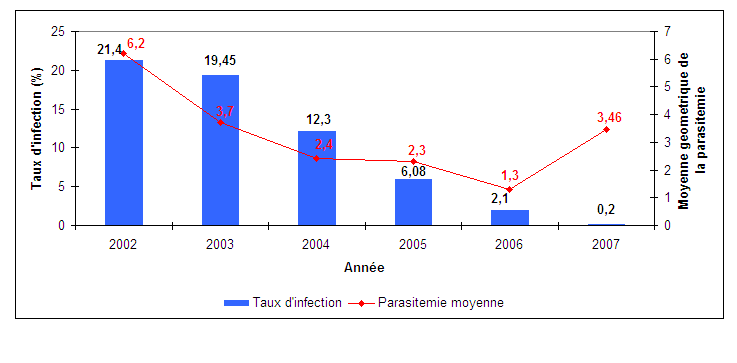


- The results of a mapping exercise of trachoma and LF conducted in June 2008 in the Sikasso region by ITI in cooperation with the *Direction Nationale de la Santé* through its national programs (*Programme National de Lutte contre la Cécité* and *Programme National d’Elimination de la Filariose lymphatique*) found a high endemicity of the two diseases in the region, with a particularly high prevalence of co-endemicity in Bougouni and Kolondiéba. The prevalence of active trachoma, Trachomatous Inflammation Follicular (TF) and Trachomatous Inflammation Intense (TI), is 14.65% and 15.14%, respectively. The prevalence of lymphatic filariasis is 14% in Bougouni and 24.4% in Kolondiéba.

**Table 2:** Population data and Trachoma and LF Prevalence in the Sikasso Region in June 2008

| **District** | **Population 2008** | **Trachoma Prevalence (%TF/TI)** | **Trachoma Prevalence (%TT)** | **LF prevalence (%)** |
| --- | --- | --- | --- | --- |
| **Bougouni** | 401,263 | 5.77 | 0.65 | 17.6 |
| **Kadiolo** | 188,574 | 2.95 | 0.80 | 7.41 |
| **Kolondieba** | 184,510 | 7.45 | 1.47 | 24.40 |
| **Koutiala** | 541,858 | 0.49 | 0.51 | 18.33 |
| **Sélingué** | 83,318 | 13.51 | 1.53 | 4.33 |
| **Sikasso** | 713,069 | 6.83 | 0.65 | 4.94 |
| **Yanfolila** | 147,566 | 12.45 | 1.74 | 4.44 |
| **Yorosso** | 192,902 | 3.73 | 1.20 | 8.80 |

**TF:** Trachomatous Inflammation Follicular

**TI:** Trachomatous Inflammation Intense

**TT:** Trachomatous Trichiasis

**Table 3:** Results of prevalence investigations (investigations and sentinel sites) and mass treatment conducted in the Sikasso region in relation to trachoma and LF.

| **Study Sites** | Trachoma prevalence based on studies | | Coverage  of  trachoma mass treatment in % | | LF prevalence in % based on studies | | | Coverage  of  LF mass treatment of in % | | | | |
| --- | --- | --- | --- | --- | --- | --- | --- | --- | --- | --- | --- | --- |
|  | Mapping 1997 | Prevalence study  2008 | 2007 | 2008 | Mapping validation study  2004 | Sentinel sites  2005 | Prevalence study  2008 | | 2005 | 2006 | 2007 | 2008 |
| **REGION** | **32** | **6.85** | **84** | **86** | **-** | **-** | **11.40** | | **-** | 80 | 82 | 81 |
| Bougouni | 31.7 | 5.77 | 82 | 84 | - | 23.2 | 17.6 | | 83 | 83 | 87 | 82 |
| Kadiolo | 31.7 | 2.95 | 87 | 95 | 20 | 24.4 | 7.41 | | - | 74 | 81 | 81 |
| Kolondieba | 31.7 | 7.45 | 90 | 86 | - | 37 | 24.40 | | 81 | 84 | 75 | 78 |
| Koutiala | 31.7 | 0.49 | 80 | 90 | - | - | 18.33 | | - | 73 | 79 | 82 |
| Yanfolila | 31.7 | 12.45 | 91 | 83 | 20 | 19.8 | 4.44 | | 87 | 87 | 87 | 82 |
| Selingue | 31.7 | 13.51 | 93 | 87 | - | - | 4.33 | | - | 80 | 84 | 85 |
| Yorosso | 31.7 | 3.73 | 92 | 92 | 16 | - | 8.80 | | - | 82 | 75 | 81 |
| Sikasso | 31.7 | 6.83 | 82 | 81 | - | - | 4.94 | | - | 72 | 78 | 82 |

**Sources:** *Reports of trachoma mapping in Mali (1997) and the Sikasso region (2008), report of the validation study of the mapping of LF in Mali in 2004, report on the prevalence of LF in sentinel sites in 2005, report on NTD mass treatment in the Sikasso region from 2005 to 2008.*

- The mass treatment coverage of around 80% has been reached each year in the Sikasso region meaning that at least 1,700,000 people have received 3 treatments with ivermectin + albendazole and 2 treatments with azithromycin between 2006 and 2008. The people of Bougouni, Kolondiéba and Yanfolila received 4 rounds of mass treatment with ivermectin and albendazole between 2005 and 2008 and 2 rounds of treatment with azithromycin in 2007 and 2008. Results of these treatments are indicated in the table above. The evolution of trachoma and LF prevalence indicated in Table 3 above shows that health and socioeconomic development interventions have had a positive impact on the epidemiological situation of the two diseases in the region. The results of the mass treatments were calculated on the basis of the general population targeted i.e. 2,028,258 people in 2008.

**3.3 Subject Selection Criteria**

The study will enroll children aged 5 years (over 90 cm) and above and adults aged 65 or less living in the Bougouni and Kolondiéba “cercles” of the Sikasso region of Mali.

***3.3.1 Eligibility Criteria***

- Must have been residing in the village for at least three months;
- Must be 90 cm tall or more;
- Must be between 5 years and 65 years of age;
- Must not be pregnant;
- Must not be lactating.

***3.3.2 Ineligibility Criteria***

- Subjects under 5 years of age or less than 90 cm in height;
- Subjects over 65 years of age;
- Subjects who cannot swallow tablets;
- Subjects who are sick and bedridden (see serious diseases in Appendix E);
- Pregnant women (clinical appreciation in the study);
- Lactating women;
- History of allergies to the drugs being studied (azithromycin, ivermectin, albendazole).

**3.4 Investigational Treatment and Surveillance**

The treatment and surveillance phase will have a 15 day duration and will involve two groups of villages in the “cercles” of Bougouni and Kolondieba: one group of villages A (control group) for the standard mass treatment and one group of villages B for the mass triple therapy.

Study drugs (ivermectin, albendazole and azithromycin) will be given based on height. The teams responsible for investigations and treatments will have standard tools to minimize selection and evaluation bias.

Administration of the drugs will be as follows (refer to Appendix A: study design and Appendix B: study flow chart):

- **Villages A (control group): Standard Treatment**

**On Day 1**, subjects included in the study will receive concomitantly a dose of ivermectin based on height (maximum four 3mg tablets) and one (1) tablet of albendazole 400 mg irrespective of height. Following administration of ivermectin and albendazole and up to Day 7, subjects will be asked to report to researchers and investigators any adverse effects.

**On Day 8**, the same subjects will be examined clinically and then they will receive an azithromycin tablet at the indicated dose based on height (up to four 250mg tablets). During this clinical examination, subjects will be asked a series of questions regarding adverse effects and the results will be recorded on the Questionnaire. They will then be asked to report any adverse events to researchers and investigators up to **7 days** following this treatment.

**On** **Day 15,** there will be another medical exam in order to assess adverse events in subjects included in the study.

- **Villages B: Triple therapy**

**On Day 1,** subjects included in the study will receive concomitantly azithromycin (up to 1 g or four 250 mg tablets), ivermectin (up to 12 mg or four 3 mg tablets) and albendazole (one 400 mg tablet per included subject irrespective of height and age). Following the triple therapy, subjects included in villages B will be asked to report to researchers any adverse effects, up to Day 7.

**On Day 8,** the same subjects will be interviewed and examined for adverse events. While treatment will have stopped, they will still be asked to continue to report adverse events that may occur up to 7 days after this clinical exam.

**On Day 15**, after the triple therapy, a further medical exam will take place to assess adverse events.

People not participating in the study should be given azithromycin as part of the National Trachoma program recommendations since this drug can be administered to children > 6 months and in lactating women, unlike ivermectin.

- **Subject Mobilization Mechanism in Villages A and B**

In both groups, investigators will be located in village health centers or, when this is not possible, on a site chosen with the agreement of the villagers. Subjects will go to the agreed site where they will be examined for potential contraindications before receiving treatment. Volunteers chosen by the villagers will guide households which will be grouped by sector and scheduled for their meeting with the investigators. These volunteers will help community leaders check that all the households have been surveyed. Subjects included in the study will report events experienced and the volunteers will look for those who do not present themselves on Day 7 and Day 15. This is a crucial step to guarantee the regular attendance of the participating subjects, ensure a smooth interaction with the volunteers and the villagers thereby encouraging the fullest participation and the best surveillance of effects.

Individuals not seen by the 14^th^ day for their day 8 clinical exam will be excluded from the study. All subjects not seen before the 17^th^ day for their Day 15 clinical exam will also be excluded from the study. Those who will have lost their identification cards will be given a copy of the stub matching the lost card.

**3.5 Sampling**

The required number of subjects will be calculated using Epi Info by comparing proportions of equal sized groups for a cohort study with a risk α of 5% and a power of 80%. The theoretical proportion of subjects having experienced adverse events after treatment with azithromycin is estimated at 5% (Pfizer, 2007). The frequency expected for the triple therapy is estimated at 8% with a confidence level of 95%. Statistical calculations with Epi Info produced a minimum sample of 1,125 subjects for each of the 2 study groups (A and B), or a total sample of 2,250 subjects. The study will take place in 4 villages: Group A and Group B will be subdivided into two subgroups A1 and A2 and B1 and B2. Each subgroup will correspond to one village. Assuming a refusal rate of 18 %, the size of each group can be increased from 1,125 to 1,328 subjects, which will be rounded up to 1,500 to obtain a sample of 3,000 subjects. This will increase the sampling power. We will thus be looking for 750 subjects per village. The population of the villages to be selected will have to be between 1,100 and 1,200, taking into account ineligible subjects and those who may be away during the study period.

## 3.6 Randomization Criteria

For this study, two villages will be assigned to the control spaced therapy and two others to the triple therapy. This will be performed by the Malian investigators with the authority of the Ministry of Health who will be supported by the Regional and District (equivalent) health authorities and the health district at the time of the launch of the study at the regional and local levels. Randomization will take place when selecting the village which is to receive the triple therapy (a coin will be tossed after assigning each side to Group A or Group B). The participation of all eligible subjects will be obtained and they will be subjected to inclusion and non inclusion criteria in each village.

***3.6.1 First Phase***

The villages will be chosen for convenience:

- To avoid problems of geographical inaccessibility, remoteness from a health facility (CSCOM, CSREFF) and proximity to each other to prevent any contamination from the intervention.
  - Villages must be within 5 km of a serviceable road, a CSCOM town or not, within 15 km of a *Centre de santé communautaire* or a *Centre de santé de référence* (CSCOM, CSREF) and there must be a distance of 15 to 20 km between villages A and B.
- To promote homogenous environmental, epidemiological and sociocultural characteristics and ensure a large enough population to provide the calculated number of subjects per village.
  - Villages located within the same district should preferably be on an East-West epidemiological axis given the endemicity/epidemiology of filariasis will be more homogenous than on a North/South axis and have a population of 1,100 to 1,200, including any hamlets and be made up of the local ethnic group and hence have similar sociocultural habits. The villages in Bougouni and Kolondiéba have the same experience in terms of mass treatment of filariasis and trachoma.

The four villages will be selected based on these criteria by the investigators with the support of the local health authorities of the Sikasso region. Two villages will be selected each in the Bougouni and Kolondiéba districts.

To identify Group A villages and Group B villages, a coin will be tossed in each health district: heads for the control group (Group A village) and tails for the investigational group (Group B village).

***3.6.2 Second Phase***

It consists in comprehensively selecting the eligible subjects through door-to-door visits to all households to conduct a census of subjects in all the villages. The target sample is 750 people per village, which represents the entire population except for ineligible persons (which is calculated to be about 25% of the population). At this stage, a map of the village will be drawn up and divided into sectors with the list of family heads to minimize the risk of census officers missing concessions or households.

***3.6.3 Third Phase***

This phase consists of obtaining the informed consent and the assent of children aged (over 90 cm) 5 to 17 and then in interviewing and physically examining consenting subjects to identify any complaints and establish that they do not suffer from a serious disease contraindicating the triple therapy (see the list of diseases contraindicating the triple therapy in Appendix E).

It is only during this phase that the inclusion of subjects in the study will be determined and finalized; the direct witnessing of drug administration will then take place. This direct witnessing is imperative, systematic and exhaustive for all consenting persons. Passive surveillance, adverse events assessment and treatment of complications of targeted diseases will also take place during this stage.

The study will need substantial awareness-raising activities within the relevant institutions and at community and individual levels. These activities will take place prior to the intervention.

**3.7 Awareness-raising**

A participatory approach will be developed from the Region towards the study sites in full compliance with the required administrative steps. This approach will be implemented during the launch of the study by the investigators (see Team composition in Section 9.1.4) who will be supported by a Regional team, a District team and a community and health area team.

***3.7.1 During the first phase***

A series of gatherings (meetings, general assemblies) will take place before the census of subjects in the four villages. Full details on the objectives and methodology of the study and the expected benefits at the local, national and international levels will be given to the subjects during those gatherings.

At the international level, an independent council made up of researchers (see section 9.1.2) will discuss the protocol in Mali and the outcomes of the implementation. It will contribute to the approach and the improvement of documents in accordance with ethical and international standards.

At the national level, the protocol will be presented to the ethics committee of the *Faculté de Médecine de Pharmacie et d'Odonstomatologie*.

At the regional level, the protocol will be presented to the executive teams of the *Direction régionale de la santé et du développement social et de l’économie solidaire* and to the regional governor.

At the level of *cercles* and health districts, the protocol will be presented to the executive teams of the *Centre de santé de référence*, the *Service de développement social et de l’économie solidaire*, the *Conseil de cercle* and the *Préfet de cercle*. Villages will be identified at this level.

At the area and township level, the protocol will be presented to the mayor and the *Association de santé communautaire*.

Finally, a general assembly will be convened in each selected village for collective informed consent.

***3.7.2 During the second phase***

A team of investigators consisting of social workers/facilitators will be deployed in the villages to conduct a census of the population. Houses will be numbered and divided into sectors and the investigators will use the administrative list of family heads available through the mayor or the village chief to identify the houses. Each identified person will be given a 4-digit identification number which will establish a connection between the individual and his/her family head and sector within the village. Example of identification number: Subject 10**A**0001 is identified as follows:

**10**: family book number

**A**: sector number

**0001**: subject number

**3.8 Study Process**

During this phase, three survey locations or stands will collaborate closely in Villages A and Villages B:

- At one station, investigators will seek the informed consent and assent of subjects and issue inclusion and exclusion cards; clinicians will interview, examine and proceed with the final inclusion of the subjects and complete survey forms;
- At a second station, investigators will measure subjects with the height pole and administer the specific drugs;
- At a third station, investigators will provide care services and evacuation referrals and update the care register. This station will also manage the non-specific drugs, the triple therapy drugs and the equipment in stock.
- Subjects excluded from the study will also receive care services and mass treatment as per usual regimen if required. To differentiate them from the other subjects, they will each be issued with a blue card indicating that they are not included in the study, the same applying to cases of refusal. These subjects will receive 7 days later the same standard mass treatment as that given to subjects in Group A villages. This task will be performed by the three teams responsible for the inclusion of participants, their treatment and other care services.

***3.8.1 Clinical examination***

This will take place after obtaining the informed consent of eligible residents.

It should focus on looking for contraindications (e.g. known allergy to a triple therapy drug, high fever; signs of impairment of liver, kidney or heart functions; treatments based on rye ergot derivatives...) for the triple therapy and also:

- identify complaints and assess the health status of the consenting subject,
- diagnose trachoma and related after-effects,
- diagnose complications of lymphatic filariasis: hydrocele and lymphoedema
- An inclusion or exclusion card will be issued to each examined person.

The investigators will be clinical physicians, medical assistants, health technicians and social workers, with experience in mass treatment and this type of activity. They will be supervised by medical researchers.

***3.8.2 Triple Therapy Drugs and Administration***

The triple therapy drugs will come from donations to the Malian NTD programme and the adverse events drugs will be bought by ITI. ITI will request the specific quantities of drugs needed for the mass treatment of villages included in the study to the Direction Nationale de la Santé and then, will make them available to the CVD.

The triple therapy will combine the administration of 3 drugs already known to the population through previous MDAs: ivermectin, albendazole, azithromycin. The administration teams will consist of 2 to 3 persons, each assigned a specific task, to ensure the proper conduct of the triple therapy under acceptable hygiene conditions and impeccable ethical standards. The investigators will have at their disposal all the required equipment and use simple and sound techniques to facilitate the ingestion of drugs. The drugs will be swallowed with the drinking water consumed by villagers using plastic bowls (250 ml maximum). Subjects will be informed of all the measures taken at the level of the village and the CSCOM to deal with possible adverse events. Drugs will be taken after a meal and this will be indicated on the survey sheet. They will be in their usual form (azithromycin as a dehydrated 250mg tablet, ivermectin as a 3mg tablet and albendazole as a 400mg tablet). The doses will depend on the height of the subjects for ivermectin and azithromycin and will be determined by one of the sides of the specifically designed height pole. A 400mg albendazole tablet will be the standard dose irrespective of the height of the subject included in the study. Drug administration will be directly witnessed by the distribution officer who will show empathy in trying to help the subjects swallow the tablets.

**Therapeutic Regimen**

- **For ivermectin + albendazole**

Ivermectin will be given based on the height of subjects while the dosage of albendazole will be standard, i.e. a 400mg tablet for all eligible persons.

**Table 4:**  Correspondence between height in cm and doses of ivermectin and albendazole tablets

| **Patient height (cm)** | **Ivermectin (3mg tablet)** | **Albendazole (400 mg tablet)** |
| --- | --- | --- |
| Less than 90 | None | None |
| 90-119 | 1 tablet | 1 tablet |
| 120-140 | 2 tablets | 1 tablet |
| 141-158 | 3 tablets | 1 tablet |
| 159 + | 4 tablets | 1 tablet |

- **For azithromycin**

In this study, it will be given based on the height of subjects rather than their weight or age. Treatment will be given to all subjects included in the sample who are at least 90 cm tall. Azithromycin tablets must be swallowed with a glass of water without chewing. Azithromycin will be given after a meal.

**Table 5:**  Correspondence between height in cm and doses of azithromycin tablets

| **Height (cm)** | **Quantity of product to be given** |
| --- | --- |
| **TABLET** | |
| 90 – 94 | 1 tablet |
| 95 – 123 | 2 tablets |
| 124 – 143 | 3 tablets |
| Over 144 | 4 tablets |

**3.9 Data Collection Tools**

The standardization of data collection methods and tools acts as a safeguard for the study. With the exception of the height pole, the tools will make up a confidential file for each subject included in the study with all the required attachments.

***3.9.1 Height Pole***

This is a double-sided pole with separate graduations (one side for azithromycin tablet and one for ivermectin). The sides will be easily differentiated using words and symbols. The distribution officers will be appropriately trained to easily handle the tools, including the height pole, the questionnaire, the informed consent form, the inclusion and exclusion cards, the census and care registers. A height pole will be designed for children aged between 6 months and 5 years who are not included in the study so that they can receive the standard treatment for trachoma or soil transmitted helminthes alone.

***3.9.2 Informed Consent Form and How to Complete Instructions for persons aged 18 and over*** It will present essential information about the survey and whether a subject is included or not in the study sample. It is intended to be user friendly. It is the only field tool that will give credibility to the legality of a person's acceptance to participate in the survey. An informed consent is to be obtained for any eligible resident of 18 years and over willing to participate in the study. This will include reading the informed consent form, and commenting on it if necessary; persons or their custodian who can read in French or the local language (Bambara) will be asked to read it themselves. Persons who accept to participate in the survey will receive a green inclusion card. Those who do not will receive a blue card indicating their exclusion from the study and will be treated according to the standard regimen for the duration of the survey. The consent form will be signed by the subjects themselves. Subjects who are unable to sign will put their fingerprint (left index finger) on the informed consent form. The investigator and a literate witness from the village will sign the form. The informed consent form will be signed in duplicate, with one copy for the subject and the other for the investigator. The informed consent will be translated in local language and recorded on audio tape for playing to the subjects in small groups.

***3.9.3 Assent Form for children aged 5 (over 90 cm) to 17***

Its content is identical to the informed consent form. It will be read and clearly explained to the children to allow them to give their consent to participate in the study. Once appropriately informed and, if in agreement, the children will sign the form together with their custodian and witness.

***3.9.4 Inclusion and Exclusion Cards and How to Complete Instructions***

Cards will be green for included subjects and blue for excluded subjects. They will come with a stub to facilitate the identification and traceability of consenting persons who are entitled to all the benefits of the study. The completed cards and stubs will be signed and the cards will be issued to their holder for the duration of the survey while the stubs will be kept by the investigators/supervisors. Green cards will only be issued to subjects included in the survey and blue cards only to subjects excluded from the study. The investigator will write on the card the identification number used during the census.

***3.9.5 Questionnaire and How to Complete Instructions***

The questionnaire is given in Appendices D3, D4, and E and comprises 6 sections: introduction, identification of the investigator and included subjects, interview and clinical examination, treatment coadministrator, surveillance of adverse events, case management and comments.

The questionnaire will record complaints existing before the triple therapy, their evolution and the occurrence of adverse events after the triple therapy. It will be completed in total safety for both parties before drug administration and during surveillance activities. Surveillance of adverse events will be passive for the 7 days following treatment. On Day 8, all treated subjects of Villages A and B will be questioned on the occurrence of adverse events by experienced health care professionals and/or researchers who will have been involved in previous health surveys performed in the country. Surveillance activities will continue for 15 days after treatment.

**3.10 Data Collection Methods**

***3.10.1 Collection of identity information and registration of complaints***

This is performed on the questionnaire. The investigator will write on the subject’s questionnaire the identification number of the inclusion card which corresponds to the identification number given during the census.

The questionnaire includes a table for recording all complaints and their evolution**.** All health complaints reported by consenting subjects will be recorded without discrimination on the survey form prior to the administration of drugs. The health care worker will consider whether or not to include the subjects in the sample. Common illnesses identified during the clinical examination will be treated. All drugs received concomitantly will be recorded on the questionnaire (see Appendix D4).

***3.10.2 Recording of Adverse Events***

The reporting of adverse events will be systematic and comply with guidelines to avoid bias related to the wording of the question as well as any active investigation of adverse events. Such events will be recorded on the questionnaire and in an examination register. The examination register will be available with the duty team and used for recording all adverse events, beneficial effects as well as the evolution of complaints observed prior to the triple therapy, and for giving the required care or advice. The questionnaires will be kept by the investigators and the care register by the health care officers. They will work in close collaboration and all information relating to the surveillance of adverse events will be available on the subjects' questionnaires while the register will allow for more detailed case recording.

Investigators will not read the list of adverse events printed on the questionnaire during surveillance and assessment activities but will ask subjects about adverse events experienced. They will listen and tick the relevant answers, mention events not listed and clarify whether complaints reported or discovered during the interview and clinical examination have improved.

Reported adverse events will be classified according to their intensity (none, minor, moderate or major).

The evolution of older complaints will be classified as: exacerbated, ongoing, improved or none.

The causal relationship of adverse events observed with the triple therapy will be assessed as: Unrelated, Unlikely or Likely.

***3.10.3 Follow-up of Serious Cases in Villages A and B***

This consists of a clinical follow-up with case management and surveillance of overall health status, temperature, blood pressure, heart rate and loss of fluids.

Follow-up will start immediately after the drugs are taken for subjects experiencing serious or major adverse reactions who may benefit from free emergency evacuation in order to receive appropriate care.

In addition to existing standard documents, a technical evacuation form will also be available. To improve the prognosis of serious or major adverse events, a 4-wheel drive vehicle will be available in study villages for evacuation purposes. The *Centre de Santé de référence du Cercle* (Health Center) and the nearest hospital will be informed of and involved in the follow-up process.

## *3.**10.4 Subject Withdrawal*

Subjects may withdraw from the study at any time at their own request, or they may be withdrawn at any time at the discretion of the investigator for patient safety reasons. If a subject does not return for a scheduled visit, every effort should be made to contact the subject. In any circumstance, every effort should be made to document subject outcome, if possible.

If the subject withdraws from the study, the investigators will analyze, with the subject's approval, any data collected before such withdrawal of consent.

**3.11 Investigator's Guide**

The Investigator’s Guide is a leaflet of a few pages designed to standardize all the services and minimize the number of technical and ethical errors while collecting data so as to facilitate the conduct of the survey. It will include advice and definitions that will assist investigators, supervisors, health care workers and evacuation teams in their work.

**4. OPERATIONAL DEFINITIONS**

**Region**: Administrative area incorporating several *cercles*. It is administered by a regional governor. The region is home to the *Direction Régionale de la Santé* (DRS) headed by a *Directeur Régional de la Santé*.

**Cercle**: Administrative area incorporating several townships. It is administered by a *Préfet de Cercle.*

**Health District**: Health zoning matching the *cercle*. A health district consists of several health areas. The medical structure at the level of the health district is called *Centre de santé de Référence* (CSREF). It is run by a Chief Medical Officer.

**Health area:** Geographical area having on average a 5 to 15 km radius and a population of 5,000 to 10,000 distributed in several villages or zones sharing a community health structure called *Centre de Santé Communautaire* (CSCOM). The CSCOM is run by a health worker or a medical officer. A township may include one or more health areas.

**Triple Therapy**: Triple therapy is defined as the triple co-administration of azithromycin + ivermectin + albendazole (AZI + IVM + ALB) at the same time to a consenting subject enrolled in the study, occurring under the direct supervision of a co-administration officer**.**

**Complaint**: A complaint is defined as any abnormal event experienced (or not) by a subject prior to the study triple therapy; it can be ongoing, exacerbated, improved or disappear during the surveillance or observation period.

**Adverse Event**: An adverse event is defined as any untoward medical occurrence in a clinical investigation patient administered a product or medical device; the event need not necessarily have a causal relationship with the treatment or usage. An adverse event is a reported new adverse effect or an existing complaint exacerbated after initiation of the treatment or an unexpected extension of hospital care.

The criteria to be used by the investigator for determining, after further analysis, whether an abnormal objective test finding should be reported as an adverse event are as follows:

- Test result is associated with accompanying symptoms, and/or
- Test result requires additional diagnostic testing or medical/surgical intervention, and/or
- Test result leads to a change in study dosing or discontinuation from the study, significant additional concomitant drug treatment, or other therapy, and/or
- Test result is considered to be an adverse event by the investigator.

Merely repeating an abnormal test, in the absence of any of the above conditions, does not constitute an adverse event. Any abnormal test result that is determined to be an error does not require reporting as an adverse event

**Serious Adverse Event**: A serious adverse event or serious adverse drug reaction is defined as any untoward medical occurrence at any dose that:

- Results in death
- Is life-threatening (immediate risk of death)
- Requires inpatient hospitalization or prolongation of existing hospitalization
- Results in persistent or significant disability/incapacity
- Results in congenital anomaly/birth defect

Medical and scientific judgment should be exercised in determining whether an event is an important medical event. An important medical event may not be immediately life-threatening and/or result in death or hospitalization. However, if it is determined that the event may jeopardize the patient and/or may require intervention to prevent one of the other adverse event outcomes, the important medical event should be reported as serious.

Examples of such events are intensive treatment in an emergency room or at home for allergic bronchospasm; blood dyscrasias or convulsions that do not result in hospitalization; or development of drug dependency or drug abuse.

**Beneficial Effects**: A beneficial effect is defined as any event experienced by a subject as a feeling of relief and well-being after the study triple therapy, with a likely or certain causality. The list will be drawn up during the surveillance or observation period.

**5. ADVERSE EVENTS ASSESSMENTS**

# 5.1 Severity Assessment

The severity will be assessed based on the intensity of adverse events, which may be minor, moderate or major.

- MINOR: does not interfere with patient's usual function
- MODERATE: interferes to some extent with patient's usual function.
- MAJOR: interferes significantly with patient's usual function

Note the distinction between a serious adverse event and a major adverse event. A major event is not necessarily a serious event. For example, a headache may be major (interferes significantly with patient's usual function) but would not be classified as serious unless it met one of the criteria for serious adverse events, listed above.

**5.2 Causality Assessment**

The investigator’s assessment of causality must be provided for all adverse events (serious and non-serious); the investigator must record the causal relationship in the CRF, as appropriate, and report such an assessment in accordance with the serious adverse reporting requirements if applicable. An investigator’s causality assessment is the determination of whether there exists a reasonable possibility that the investigational product caused or contributed to an adverse event. If the investigator’s final determination of causality is unknown and the investigator does not know whether or not investigational product caused the event, then the event will be handled as “related to investigational product” for reporting purposes. If the investigator's causality assessment is "unknown but not related to investigational product", this should be clearly documented on subject’s records.

In addition, if the investigator determines a serious adverse event is associated with study procedures, the investigator must record this causal relationship in the questionnaire, the clinical exam register and the case referral form as appropriate. The assessment will be performed in accordance with the serious adverse event reporting requirements, if applicable. The Ethics Committee must be notified within 72 hours of information reaching the investigators.

**5.3 Safety and Feasibility Evaluation of the Triple Therapy Study**

There are no tangible criteria for assessing the safety and feasibility of the triple therapy as their appreciation can vary from country to country in view of the epidemiological and socioeconomic impact of the targeted diseases and the political commitment.

**Safety**: The appreciation will depend on the interpretation given by the *Ministère de la Santé du Mali* of the number of serious cases observed, their incidence compared to the burden of the treated diseases (mortality, morbidity, handicap, economic impact, etc.), the benefits of the triple therapy to the community and the safety of scaling it up at the district level. The incidence of serious adverse events, as defined in Section 4 will be determined on the basis of causal relationships assessed by the investigators and the DSMB.

**Feasibility**: The appreciation will also depend on the interpretation given by the *Ministère de la Santé* of the results of the logistical approach. However, the safety of the triple therapy could be deemed acceptable if the incidence of adverse events observed is similar to that of standard treatments with azithromycin and ivermectin+albendazole.

**6. DATA ANALYSIS**

**6.1 Data Acquisition and Presentation**

Data will be entered in Access and analyzed with statistical software packages SPSS version 12 and Epi Info, SAS version 9. Data will be summarized using descriptive statistics. Categorical variables will be summarized using percentages. For each continuous variable, the mean, median and standard deviation will be computed.

Parameters to be determined are:

- Population of study villages: composition by age group and sex.
- Rate of therapeutic coverage in Villages A and B.
- Prevalence and incidence of adverse events by age group, sex, and village
- Trends in adverse events in each village/group of villages
- Severity of adverse events in each village/group of villages
- Average time before the occurrence of the first adverse events
- Minimum and maximum time after which an adverse event can be observed
- Average age of subjects experiencing adverse events

The analysis will be based on all subjects who received at least one dose of study drug.

**6.2 Primary Analysis**

Percentage of subjects with at least one adverse event will be calculated for each group. In addition the percentage will be calculated for each adverse event by drug-relatedness and severity. For the latter, if a subject has more than one occurrence of the same event, the most severe occurrence will be considered.

**6.3 Comparative Analyses**

In order to compare prevalence, the incidence of adverse events after treatment between villages and visits will be compared using Pearson's chi-square test, Yates' chi-square test or Fisher's exact test.

A multivariate analysis will also be performed to determine the most significant risk factors in the occurrence of adverse events, in particular the most serious and most frequent events.

For continuous variables, non-parametric tests (Wilcoxon rank sum test or Mann-Whitney U) will be used to compare the 2 treatment groups.

**6.4 Adverse Events**

The descriptions of adverse events as well as their incidence and severity will be summarized. Adverse events reported several times will also be summarized.

#### Physical examination information will be tabulated by status (normal or abnormal).

**6.5 Demographic and baseline characteristics**

Sex, age, weight, height, and other relevant demographic characteristics will be summarized using means, standard deviations, and ranges for continuous data and frequencies for categorical data.

**6.6 Medical History**

Medical history relevant to the study will be summarized.

**6.7 Drug and non-drug treatment**

All prior medications not related to the primary diagnosis and all prior medications that a subject continues to take during the study will be summarized. Concomitant non-drug treatment refers to all those taken during the effective duration of study treatment (from Day 1 to Day 15), whether or not they are recorded at baseline.

# 7. ETHICAL CONSIDERATIONS

The protocol will be submitted, before the start of the study, to the double approval of the Ethics Committee of the *Faculté de Médecine de Pharmacie et d'Odonstomatologie* (Faculty of Medicine, Pharmacy and Odonto-stomatology - FMPOS) and the IRB Ethics Committee of the CVD, University of Maryland, Baltimore, USA.

**7.1 Study Risks**

Clinical examinations cannot pose a major risk as the implementation of strict aseptic measures will minimize any risk of contamination during the study. These will include:

- Disinfecting examiners' hands and technical equipment with 70% ethanol;
- Examiners wearing protection gloves during examinations**;**
- Using single-use sterile equipment, disinfecting the skin at the point of collection of blood samples;
- Packaging sharps and biomedical waste in safety boxes or sealed bins.
- Adverse events associated with the three study drugs are known when the drugs are administered separately. They are rare and mild in intensity.

**7.2 Study Benefits**

The benefits of the study include:

***7.2.1 Benefits for surveyed populations***

- Children with signs of evolutive trachoma will be treated free of charge by the survey team.
- Cases of trichiasis and hydrocele observed during the clinical examinations will be treated by submission for surgical procedures.
- Subjects suffering from trichiasis will be treated free of charge, subject to their approval, by a special mission of trichiasis operators when all the survey data will be collected. A team of trichiasis operators will be deployed in each village to treat all identified TT cases starting from the 3rd week of the survey.
- Subjects suffering from hydrocele will be treated subject to their approval. They will be referred to the nearest operating room where they will be treated at the same time as the cases of trichiasis.
- Subjects suffering from lymphoedema will receive free medical treatment on site subject to their approval and instructed in home-based care of the affected limbs by being provided with equipment (plastic buckets, soap and towels for limb washing and care; antibiotic and antifungal cream).
- Medical care will be given to cases of elephantiasis and other lymphoedema for the duration of the study.
- Cases of fever with presumed or confirmed malaria will be divided into patients requiring concomitant treatment and patients whose inclusion in the sample should be delayed according to their state of health.
- Other eye infections showing no improvement in children after the triple therapy will be treated with tetracycline ointment after the collection of survey data.
- Increased population awareness of the adoption of behaviors conducive to the prevention and treatment of trachoma and filariasis.

***7.2.2 Benefits for technical services***

- Producing a trachoma and filariasis database for assessing the impact of future actions;
- Survey forms and basic data will be stored by ITI and remain confidential until 2011 when the project completes.
- The buddy system used during the study period will contribute to the training by specialists of part of the districts' medical staff.

***7.2.3 Compensation***

Compensation measures in compliance with good clinical practices will be applied and include giving a piece of “Koulikoro” soap to each person or family examined.

# 8. FORMAT OF THE STUDY REPORT

The study outcomes will be recorded in a report in Word format and a PowerPoint presentation. They will be validated during a meeting of all the people involved in the protocol validation process, all the other human health research institutions (INRSP, MRTC, CREDOS, DERSP), the partners working on NTDs and blindness control and the representatives of the direct beneficiaries of the study.

The final report will take into account the observations and suggestions made.

Once amended accordingly, the report will be distributed at the national level with the involvement of the *Direction Régionale de la Santé* of the Sikasso region and submitted to the Health Minister's office to facilitate the implementation of the next stages (progressive scaling-up).

Survey outcomes will be circulated to the populations concerned. They will be published in international scientific journals. The report will also be used to assess the impact of further actions.

**9. RESOURCES**

**9.1 Human Resources**

This study will require a large number of persons and vast expertise. A human resources ledger will be implemented to document the different tasks assigned. This ledger will help monitor the roles and division of duties.

***9.1.1 Scientific Group Pharmacovigilance Study***

National researchers in charge of designing and implementing research at the national level. They are responsible for the successful outcome of the study. They will visit field teams and ensure the timely discharge of the various tasks.

- Professor Samba SOW (CVD): Principal Investigator
- Dr Moussa Hama Sankaré (ITI): Co-investigator
- Dr Yaya Coulibaly (CNAM/MRTC): Co-investigator
- Dr Mahamadou Keita (CVD): Co-investigator
- Dr Sory Ibrahima Bamba (DNS): Co-investigator
- Pr Mamadou Soungalo Traoré (FMPOS): Co-investigator
- Dr Fadima Cheick Haïdara (CVD) : Co-investigator

***9.1.2 Independent Data Safety Board***

National and international researchers who will provide guidance for the study include:

- Dr Baboua Traoré (Direction Nationale de la santé/Ministère de la Santé du Mali)
- Dr Moussa Yattara (Direction Nationale de la santé/Ministère de la Santé du Mali)
- Professor David Molyneux (Liverpool School of Tropical Medicine)
- Dr Boakye Boatin (WHO TDR Geneva)
- Dr Massambou Sacko (WHO-Mali)
- Dr Charles Knirch (Pfizer Inc.)
- Dr Emma Andrews (Pfizer Inc.)
- Dr Els Mathieu (Centers for Disease Control and Prevention, Atlanta)
- Dr Danny Haddad (ITI)
- Ms Lisa Rotondo (ITI)
- Ms PJ Hooper (ITI)
- Dr Antandou Telly (ITI-Mali)

***9.1.3 CVD Team***

The survey will be preferably performed by officers with a good experience of surveys.

***9.1.4 Team conducting the initial village identification mission***

Team of 7 people and two drivers on duty for 7 days.

Dr Sory Ibrahima Bamba (Direction Nationale de la Santé/Ministère de la Santé du Mali),

Dr Antandou Telly (ITI), Dr Moussa Hama Sankaré (ITI)

Dr Yaya Coulibaly (CNAM/MRTC)

Dr Kéita Mahamadou (CVD)

A representative from the Sikasso DRS

One representative from the Bougouni CSREF and one from the Kolondiéba CSREF

A representative from the CSCOM associated with the selected villages

***9.1.5 National Data Safety Monitoring Board (DSMB)***

It is made up of several specialists (1 pharmacist, 1 pediatrician, 1 internist, 1 epidemiologist, 1 socio-anthropologist) to facilitate the treatment of evacuees and referred patients, to **take care that regulatory provisions are followed and that the best case investigation practices are implemented**, to **ensure the efficient management of serious cases,** and to investigate such cases. The DSMB will meet at least twice during the time of the study and will communicate via the Internet. The Principal Investigator and the Research Coordinator will inform the DSMB daily. The DSMB will monitor surveillance data daily during the entire period of the study and one month after the study. The following experts will be part of the DSMB:

1. Pr Marouf Keita, Pediatrician (FMPOS)

2. Pr Hamar Alassane Traoré, Internist (FMPOS)

3. Dr. Samba Diop, Socio-anthropologist (FMPOS)

4. Dr. Ibrahim Coulibaly, Pharmacist (Inspection de la Santé)

5. Pr David Molyneux (Liverpool School of Tropical Medicine)

***9.1.6 Social Worker Teams***

For raising the awareness of and identifying people living in villages selected for the study, assessing the equipment available at CSCOM and CSREF for the purposes of the study (24 social workers and 2 supervisors, 2 drivers for 7 days) for 70 informed and identified persons (or 10 visited households) per day and two-person team.

***9.1.7 Data Monitoring Council***

This council is made up of at least two field supervisors per village, one co-investigator and one research coordinator. They are responsible for the proper implementation of the adopted methodology and of all phases of the study (awareness-raising phase, survey phase and treatment phase for cases of trichiasis and hydrocele). They will also coordinate administrative matters with the competent health authorities (travel order, liaison with administrative, health and customary authorities).They will be in charge of verifying and validating all the data collected by the teams and will provide a report on the study activities which will be transmitted to the scientific group together with the validated data. The Council will also be responsible for the presentation of the survey on the field during a report to the various authorities at the time of withdrawal.

***9.1.8 Investigator Teams***

The investigators will be health workers and social development officers meeting the following criteria:

- Have experience in similar studies: clinical trials, pharmacovigilance, etc.
- Have experience in ophthalmologic surveys, in filariasis, and managing a NTD mass treatment team
- Have appropriate clinical and ethical experience
- Be available for the duration of the survey
- Be able to work in a team environment.

The investigator teams will be split into three (3) critical stations:

- **Station 1**: The team seeking the patients' informed consent: 2 two-person teams per village i.e.: 2 social workers per village or 8 persons for the study for the day of card issuance and treatment and the days of surveillance.

This team will interview and examine the consenting subjects and issue the inclusion and exclusion cards. There will be 3 two-person teams per village i.e.: 24 health workers (2 ophthalmologists, 2 doctors, 2 health technicians) per village during the days of treatment and surveillance. This team will also be responsible for passive and active surveillance and mass treatment of subjects not enrolled in the study.

- **Station 2**: The team giving treatment in the villages: 2 two-person teams per village i.e.: 8 health workers (1 doctor and 1 health technician) per village for 15 days of close follow-up of treated subjects;
- **Station 3**: The team in charge of logistics and administration: 10 people (one administrator, one secretary, one electrician, one cook and one laborer). There will be one team of logisticians per health district (5 people per district). These teams will be supported by at least one village volunteer per station.

***9.1.9 The team giving treatment to evacuees and referred patients at the district level and in hospitals***

Normal CSREF and hospital duty teams used for evacuation in the national health system: duty teams from the Bougouni and Kolondieba CSREF and the Sikasso hospital will be involved and motivated.

***9.1.10 The team operating the trichiasis after the survey***

One team of ophthalmology assistants with a driver who will travel to the four villages for a total of 10 days (5 days per district). This team will also organize the referral of hydrocele cases to CSREF as well as their management and follow-up.

***9.1.11 Data entry officers***

4 persons experienced in entering survey data. They will be responsible for the entry of data collected in the field. Data will be entered in two separate files that will be compared and any discrepancy will be corrected using the questionnaires. The “Compare Wizz” software application will be used to track differences between the two Access files.

**9.2 Material Resources**

***9.2.1 Logistics and consumables***

- Scientific group team: 3 vehicles (2 for supervision, 1 for coordination)
- Village identification mission: 2 vehicles
- Awareness-raising and census team: 2 vehicles and 12 motorbikes (?)
- Monitoring Council: 2 vehicles
- Survey Team: 8 vehicles including one ambulance and 12 motorbikes
- Trichiasis Surgery Team: 1 vehicle
- 2 x 1kW generators and 6 x 20 liter jerry cans
- Consumables kit (fuel, gas, batteries, wicks, candles, bulbs...)

***9.2.2 Equipment***

- 20 beds, 20 light mattresses, 20 thick mattresses per village
- 20 impregnated mosquito nets per village, 30 plastic mats, 2 hammers, 2 pliers, 4 screwdrivers
- 8 medium-sized camping tarpaulins (1 per station), 20 individual tarpaulins per village
- 4 iceboxes, 4 thermoses
- 200 meters of rope
- Cooking equipment (stoves, pots, cups, plates, spoons, forks, knives, coffee pots, tea pots, kettles...)
- 2 Orange mobile phones with Orange credit
- 4 metal crates for drug kits
- Medical equipment: sphygmomanometers, stethoscopes, height poles, thermometers, buckets, cups, pots, trachoma diagnostic kits (8 binocular loupes, 8 flashlights, protection gloves, Koulikoro soap cakes)
- Medical emergency kit (Anti-tetanus serum:ATS, antivenin, Phenergan, glucose 50%, plasma, Solumedrol, Ephidrine…)
- Laboratory kits (TDR, gloves)
- 8 folding tables; 20 folding chairs and 10 benches
- 4 folding screens
- Office equipment: 2 PCs, 2 printers, 2 photocopiers, 2 UPS units, 2 heavy-duty staplers with staples, 2 rolls of sticky tape, 2 boxes of files/folders, 1 multimedia, 2 flipcharts

***9.2.3 Office supplies and survey tools***

- 4,500 survey sheets
- 4,500 inclusion and exclusion cards
- 200 surveillance sheets:
- 50 evaluation sheets
- 100 stock sheets
- 8 consultation registers
- 2 hospitalization registers
- 50 x 100-page census books
- 40 strap folders
- 40 notebooks
- Pencils: 4 boxes
- 40 rubbers:
- 40 pencil sharpeners
- 1 box of ball-point pens
- 2 rolls of large-size paper
- 24 felt pens
- 40 reams of paper
- 2 photocopier toner cartridges
- 2 printer toner cartridges

***9.2.4 Drugs***

- Drug kit (trichiasis, malaria, serious reactions, minor reactions)
- Triple therapy drugs (albendazole = 5,000, ivermectin = 30,000, azithromycin = tablets 15,000, syrup 1,000, tetracycline ointment = 500)
- Bleach: 10 l, Betadine: 2 l, Absorbent cotton: 10 packs, Surgical spirit: 20 l.

**9.3 Financial resources and budget estimated at 80 millions**

**10:** TIME DIAGRAM

| **Activities, implementation period and person in charge** | | | **May** | | | | **June** | | | | **July** | | | | **August** | | | | **September** | | | | **October** | | | |
| --- | --- | --- | --- | --- | --- | --- | --- | --- | --- | --- | --- | --- | --- | --- | --- | --- | --- | --- | --- | --- | --- | --- | --- | --- | --- | --- |
| **Activities** | **Days** | **Action taken by** | S1 | S2 | S3 | S4 | S1 | S2 | S3 | S4 | S1 | S2 | S3 | S4 | S1 | S2 | S3 | S4 | S1 | S2 | S3 | S4 | S1 | S2 | S3 | S4 |
| Protocol development | 45 | ITI/CVD, National researchers |  |  |  |  |  |  |  |  |  |  |  |  |  |  |  |  |  |  |  |  |  |  |  |  |
| Submission to Ethics Committee | 15 | ITI/CVD |  |  |  |  |  |  |  |  |  |  |  |  |  |  |  |  |  |  |  |  |  |  |  |  |
| Submission to Ministry of Health | 7 | ITI/CVD |  |  |  |  |  |  |  |  |  |  |  |  |  |  |  |  |  |  |  |  |  |  |  |  |
| Preparation of service contracts (CVD, transport, data entry officers, administration team, enq...) | 30 | ITI/CVD |  |  |  |  |  |  |  |  |  |  |  |  |  |  |  |  |  |  |  |  |  |  |  |  |
| Setting up of monitoring system (PV) | 10 | Scientific group/CVD |  |  |  |  |  |  |  |  |  |  |  |  |  |  |  |  |  |  |  |  |  |  |  |  |
| Study launching mission | 10 | Scientific group/CVD |  |  |  |  |  |  |  |  |  |  |  |  |  |  |  |  |  |  |  |  |  |  |  |  |
| Identification of census officers and creation of DSMB | 7 | ITI/CVD |  |  |  |  |  |  |  |  |  |  |  |  |  |  |  |  |  |  |  |  |  |  |  |  |
| Preparation of the census | 2 | Scientific group/CVD |  |  |  |  |  |  |  |  |  |  |  |  |  |  |  |  |  |  |  |  |  |  |  |  |
| Training of census officers | 2 | Scientific group/CVD |  |  |  |  |  |  |  |  |  |  |  |  |  |  |  |  |  |  |  |  |  |  |  |  |
| Census implementation (data collection) | 7 | Monitoring council/CVD |  |  |  |  |  |  |  |  |  |  |  |  |  |  |  |  |  |  |  |  |  |  |  |  |
| Purchase of equipment and other inputs | 7 | ITI/CVD |  |  |  |  |  |  |  |  |  |  |  |  |  |  |  |  |  |  |  |  |  |  |  |  |
| Preparation of census report | 7 | CVD |  |  |  |  |  |  |  |  |  |  |  |  |  |  |  |  |  |  |  |  |  |  |  |  |
| Recruitment of investigators | 7 | CVD |  |  |  |  |  |  |  |  |  |  |  |  |  |  |  |  |  |  |  |  |  |  |  |  |
| Identification of administrative team | 5 | ITI/CVD |  |  |  |  |  |  |  |  |  |  |  |  |  |  |  |  |  |  |  |  |  |  |  |  |
| Training of co-administration investigators | 3 | CVD |  |  |  |  |  |  |  |  |  |  |  |  |  |  |  |  |  |  |  |  |  |  |  |  |
| Facilities and equipment for survey team | 2 | Scientific group/CVD |  |  |  |  |  |  |  |  |  |  |  |  |  |  |  |  |  |  |  |  |  |  |  |  |
| Data collection (field phase) | 11 | Investigators and supervisors/CVD |  |  |  |  |  |  |  |  |  |  |  |  |  |  |  |  |  |  |  |  |  |  |  |  |
| Treatment of subjects identified in the study | 2 | Investigators and supervisors/CVD |  |  |  |  |  |  |  |  |  |  |  |  |  |  |  |  |  |  |  |  |  |  |  |  |
| Withdrawal of survey teams and equipment | 2 | Scientific group/CVD |  |  |  |  |  |  |  |  |  |  |  |  |  |  |  |  |  |  |  |  |  |  |  |  |
| Report to local authorities | 2 | Monitoring council/CVD |  |  |  |  |  |  |  |  |  |  |  |  |  |  |  |  |  |  |  |  |  |  |  |  |
| Identification of data collection officers | 7 | CVD |  |  |  |  |  |  |  |  |  |  |  |  |  |  |  |  |  |  |  |  |  |  |  |  |
| Computer entry | 10 | Data collection officers/Scientific group/CVD |  |  |  |  |  |  |  |  |  |  |  |  |  |  |  |  |  |  |  |  |  |  |  |  |
| Data processing and analysis | 15 | Scientific group/CVD |  |  |  |  |  |  |  |  |  |  |  |  |  |  |  |  |  |  |  |  |  |  |  |  |
| Preparation of case treatment team: TT and hydrocele | 7 | PNLC/PNEFL/ITI/ CVD |  |  |  |  |  |  |  |  |  |  |  |  |  |  |  |  |  |  |  |  |  |  |  |  |
| Treatment of TT and hydrocele cases | 20 | OPT/PNLC/PNEFL/  CSREF/ITI/CVD |  |  |  |  |  |  |  |  |  |  |  |  |  |  |  |  |  |  |  |  |  |  |  |  |
| Report on the treatment of TT and hydrocele cases | 2 | OPT/CSREF team |  |  |  |  |  |  |  |  |  |  |  |  |  |  |  |  |  |  |  |  |  |  |  |  |
| Co-administration preliminary report | 15 | Scientific group/CVD |  |  |  |  |  |  |  |  |  |  |  |  |  |  |  |  |  |  |  |  |  |  |  |  |
| Presentation of report and validation at the national level | 1 | Scientific group/CVD |  |  |  |  |  |  |  |  |  |  |  |  |  |  |  |  |  |  |  |  |  |  |  |  |
| Final Report | 2 | Scientific group/CVD |  |  |  |  |  |  |  |  |  |  |  |  |  |  |  |  |  |  |  |  |  |  |  |  |
| Presentation of report and validation at the regional level (Sikasso) | 1 | Scientific group/CVD |  |  |  |  |  |  |  |  |  |  |  |  |  |  |  |  |  |  |  |  |  |  |  |  |
| Preparation of the publication |  | Scientific group/CVD |  |  |  |  |  |  |  |  |  |  |  |  |  |  |  |  |  |  |  |  |  |  |  |  |

**11. BIBLIOGRAPHICAL REFERENCES**

Amsden GW, Gregory TB, Michalak CA, Glue P, Knirsch CA (2007). Pharmacokinetics of azithromycin and the combination of ivermectin and albendazole when administered alone and concurrently in healthy volunteers. *Am J Trop Med Hyg.* 76 (6):1153-1157.

Direction Nationale de la Santé, 2007, “Plan stratégique de lutte contre les maladies tropicales négligées, 2007-2011”, Bamako, Mali.

GlaxoSmithKline (2003). Consumer Medicine Information, ZENTEL® (albendazole). Visited 10 March 2009 at <http://www.gsk.com.au/resources.ashx/prescriptionmedicinesproductschilddatadownloads/527/File/A7E87EACB287E55DFC66DEA3F77FC4FB/CMI_Zentel.pdf>

[Guzzo CA](http://www.ncbi.nlm.nih.gov/sites/entrez?Db=pubmed&Cmd=Search&Term="Guzzo CA"%5BAuthor%5D&itool=EntrezSystem2.PEntrez.Pubmed.Pubmed_ResultsPanel.Pubmed_DiscoveryPanel.Pubmed_RVAbstractPlus), [Furtek CI](http://www.ncbi.nlm.nih.gov/sites/entrez?Db=pubmed&Cmd=Search&Term="Furtek CI"%5BAuthor%5D&itool=EntrezSystem2.PEntrez.Pubmed.Pubmed_ResultsPanel.Pubmed_DiscoveryPanel.Pubmed_RVAbstractPlus), [Porras AG](http://www.ncbi.nlm.nih.gov/sites/entrez?Db=pubmed&Cmd=Search&Term="Porras AG"%5BAuthor%5D&itool=EntrezSystem2.PEntrez.Pubmed.Pubmed_ResultsPanel.Pubmed_DiscoveryPanel.Pubmed_RVAbstractPlus), [Chen C](http://www.ncbi.nlm.nih.gov/sites/entrez?Db=pubmed&Cmd=Search&Term="Chen C"%5BAuthor%5D&itool=EntrezSystem2.PEntrez.Pubmed.Pubmed_ResultsPanel.Pubmed_DiscoveryPanel.Pubmed_RVAbstractPlus), [Tipping R](http://www.ncbi.nlm.nih.gov/sites/entrez?Db=pubmed&Cmd=Search&Term="Tipping R"%5BAuthor%5D&itool=EntrezSystem2.PEntrez.Pubmed.Pubmed_ResultsPanel.Pubmed_DiscoveryPanel.Pubmed_RVAbstractPlus), [Clineschmidt CM](http://www.ncbi.nlm.nih.gov/sites/entrez?Db=pubmed&Cmd=Search&Term="Clineschmidt CM"%5BAuthor%5D&itool=EntrezSystem2.PEntrez.Pubmed.Pubmed_ResultsPanel.Pubmed_DiscoveryPanel.Pubmed_RVAbstractPlus), et al. (2002). Safety, tolerability, and pharmacokinetics of escalating high doses of ivermectin in healthy adult subjects. [*J Clin Pharmacol.*](javascript:AL_get(this, 'jour', 'J Clin Pharmacol.');) 42(10):1122-1133.

[Horton J](http://www.ncbi.nlm.nih.gov/sites/entrez?Db=pubmed&Cmd=Search&Term="Horton J"%5BAuthor%5D&itool=EntrezSystem2.PEntrez.Pubmed.Pubmed_ResultsPanel.Pubmed_DiscoveryPanel.Pubmed_RVAbstractPlus), [Witt C](http://www.ncbi.nlm.nih.gov/sites/entrez?Db=pubmed&Cmd=Search&Term="Witt C"%5BAuthor%5D&itool=EntrezSystem2.PEntrez.Pubmed.Pubmed_ResultsPanel.Pubmed_DiscoveryPanel.Pubmed_RVAbstractPlus), [Ottesen EA](http://www.ncbi.nlm.nih.gov/sites/entrez?Db=pubmed&Cmd=Search&Term="Ottesen EA"%5BAuthor%5D&itool=EntrezSystem2.PEntrez.Pubmed.Pubmed_ResultsPanel.Pubmed_DiscoveryPanel.Pubmed_RVAbstractPlus), [Lazdins JK](http://www.ncbi.nlm.nih.gov/sites/entrez?Db=pubmed&Cmd=Search&Term="Lazdins JK"%5BAuthor%5D&itool=EntrezSystem2.PEntrez.Pubmed.Pubmed_ResultsPanel.Pubmed_DiscoveryPanel.Pubmed_RVAbstractPlus), [Addiss DG](http://www.ncbi.nlm.nih.gov/sites/entrez?Db=pubmed&Cmd=Search&Term="Addiss DG"%5BAuthor%5D&itool=EntrezSystem2.PEntrez.Pubmed.Pubmed_ResultsPanel.Pubmed_DiscoveryPanel.Pubmed_RVAbstractPlus), [Awadzi K](http://www.ncbi.nlm.nih.gov/sites/entrez?Db=pubmed&Cmd=Search&Term="Awadzi K"%5BAuthor%5D&itool=EntrezSystem2.PEntrez.Pubmed.Pubmed_ResultsPanel.Pubmed_DiscoveryPanel.Pubmed_RVAbstractPlus) et al. (2000). An analysis of the safety of the single dose, two drug regimens used in programmes to eliminate lymphatic filariasis. [*Parasitology.*](javascript:AL_get(this, 'jour', 'Parasitology.');) 121 Suppl: S147-160.

[Keiser PB](http://www.ncbi.nlm.nih.gov/sites/entrez?Db=pubmed&Cmd=Search&Term="Keiser PB"%5BAuthor%5D&itool=EntrezSystem2.PEntrez.Pubmed.Pubmed_ResultsPanel.Pubmed_DiscoveryPanel.Pubmed_RVAbstractPlus), [Coulibaly YI](http://www.ncbi.nlm.nih.gov/sites/entrez?Db=pubmed&Cmd=Search&Term="Coulibaly YI"%5BAuthor%5D&itool=EntrezSystem2.PEntrez.Pubmed.Pubmed_ResultsPanel.Pubmed_DiscoveryPanel.Pubmed_RVAbstractPlus), [Keita F](http://www.ncbi.nlm.nih.gov/sites/entrez?Db=pubmed&Cmd=Search&Term="Keita F"%5BAuthor%5D&itool=EntrezSystem2.PEntrez.Pubmed.Pubmed_ResultsPanel.Pubmed_DiscoveryPanel.Pubmed_RVAbstractPlus), [Traore D](http://www.ncbi.nlm.nih.gov/sites/entrez?Db=pubmed&Cmd=Search&Term="Traore D"%5BAuthor%5D&itool=EntrezSystem2.PEntrez.Pubmed.Pubmed_ResultsPanel.Pubmed_DiscoveryPanel.Pubmed_RVAbstractPlus), [Diallo A](http://www.ncbi.nlm.nih.gov/sites/entrez?Db=pubmed&Cmd=Search&Term="Diallo A"%5BAuthor%5D&itool=EntrezSystem2.PEntrez.Pubmed.Pubmed_ResultsPanel.Pubmed_DiscoveryPanel.Pubmed_RVAbstractPlus), [Diallo DA](http://www.ncbi.nlm.nih.gov/sites/entrez?Db=pubmed&Cmd=Search&Term="Diallo DA"%5BAuthor%5D&itool=EntrezSystem2.PEntrez.Pubmed.Pubmed_ResultsPanel.Pubmed_DiscoveryPanel.Pubmed_RVAbstractPlus) et al. (2003). Clinical characteristics of post-treatment reactions to ivermectin/albendazole for Wuchereria bancrofti in a region co-endemic for Mansonella perstans. [*Am J Trop Med Hyg.*](javascript:AL_get(this, 'jour', 'Am J Trop Med Hyg.');) 69(3):331-335

[Knirsch C](http://www.ncbi.nlm.nih.gov/sites/entrez?Db=pubmed&Cmd=Search&Term="Knirsch C"%5BAuthor%5D&itool=EntrezSystem2.PEntrez.Pubmed.Pubmed_ResultsPanel.Pubmed_DiscoveryPanel.Pubmed_RVAbstractPlus) (2007). Trachoma: ancient scourge, disease elimination, and future research. [*Curr Infect Dis Rep.*](javascript:AL_get(this, 'jour', 'Curr Infect Dis Rep.');) 9(1):21-28.

Loukas A, Hotez PJ (2000). Chemotherapy of helminth infections. In Brunton LL, Lazo JS, Parker KL (Ed.) *Goodman and Gilman’s The Pharmacological Basis of Therapeutics, 11^th^ Edition.*. (pp. 1073-1093). New York, McGraw-Hill.

[Mabey D](http://www.ncbi.nlm.nih.gov/sites/entrez?Db=pubmed&Cmd=Search&Term="Mabey D"%5BAuthor%5D&itool=EntrezSystem2.PEntrez.Pubmed.Pubmed_ResultsPanel.Pubmed_DiscoveryPanel.Pubmed_RVAbstractPlus), [Bailey R](http://www.ncbi.nlm.nih.gov/sites/entrez?Db=pubmed&Cmd=Search&Term="Bailey R"%5BAuthor%5D&itool=EntrezSystem2.PEntrez.Pubmed.Pubmed_ResultsPanel.Pubmed_DiscoveryPanel.Pubmed_RVAbstractPlus) (1999). Eradication of trachoma worldwide. [Br J Ophthalmol.](javascript:AL_get(this, 'jour', 'Br J Ophthalmol.');) 83 (11):1261-1263

Merck & Co., Inc. (1998). Stromectol ®, Ivermectin tablets. Prescribing information. Visited 20 February 2009 at http://www.merck.com/mmpe/sec14/ch182/ch182e.html#sec14-ch182-ch182e-1808.

[Mohammed KA](http://www.ncbi.nlm.nih.gov/sites/entrez?Db=pubmed&Cmd=Search&Term="Mohammed KA"%5BAuthor%5D&itool=EntrezSystem2.PEntrez.Pubmed.Pubmed_ResultsPanel.Pubmed_DiscoveryPanel.Pubmed_RVAbstractPlus), [Haji HJ](http://www.ncbi.nlm.nih.gov/sites/entrez?Db=pubmed&Cmd=Search&Term="Haji HJ"%5BAuthor%5D&itool=EntrezSystem2.PEntrez.Pubmed.Pubmed_ResultsPanel.Pubmed_DiscoveryPanel.Pubmed_RVAbstractPlus), [Gabrielli AF](http://www.ncbi.nlm.nih.gov/sites/entrez?Db=pubmed&Cmd=Search&Term="Gabrielli AF"%5BAuthor%5D&itool=EntrezSystem2.PEntrez.Pubmed.Pubmed_ResultsPanel.Pubmed_DiscoveryPanel.Pubmed_RVAbstractPlus), [Mubila L](http://www.ncbi.nlm.nih.gov/sites/entrez?Db=pubmed&Cmd=Search&Term="Mubila L"%5BAuthor%5D&itool=EntrezSystem2.PEntrez.Pubmed.Pubmed_ResultsPanel.Pubmed_DiscoveryPanel.Pubmed_RVAbstractPlus), [Biswas G](http://www.ncbi.nlm.nih.gov/sites/entrez?Db=pubmed&Cmd=Search&Term="Biswas G"%5BAuthor%5D&itool=EntrezSystem2.PEntrez.Pubmed.Pubmed_ResultsPanel.Pubmed_DiscoveryPanel.Pubmed_RVAbstractPlus), [Chitsulo L](http://www.ncbi.nlm.nih.gov/sites/entrez?Db=pubmed&Cmd=Search&Term="Chitsulo L"%5BAuthor%5D&itool=EntrezSystem2.PEntrez.Pubmed.Pubmed_ResultsPanel.Pubmed_DiscoveryPanel.Pubmed_RVAbstractPlus) et al. (2008). Triple co-administration of ivermectin, albendazole and praziquantel in Zanzibar: a safety study. [*PLoS Negl Trop Dis.*](javascript:AL_get(this, 'jour', 'PLoS Negl Trop Dis.');) 2(1):e171

Na-Bangchang K, Kietinun S, Pawa KK, Hanpitakpong W, Na-Bangchang C, Lazdins J (2006). [Assessments of pharmacokinetic drug interactions and tolerability of albendazole, praziquantel and ivermectin combinations.](http://www.ncbi.nlm.nih.gov/pubmed/16271272?ordinalpos=2&itool=EntrezSystem2.PEntrez.Pubmed.Pubmed_ResultsPanel.Pubmed_DefaultReportPanel.Pubmed_RVDocSum) *Trans R Soc Trop Med Hyg.* 100(4):335-345. Epub 2005 Nov 2.

Ottesen EA, Ismail MM, Horton J (1999). The role of albendazole in programmes to eliminate lymphatic filariasis. *Parasitology Today*, 15(9):382-386.

Pfizer Inc (2007). Zithromax®, azithromycin tablets and azithromycin for oral suspension. Prescribing information. Visited 15 March 2009 at http://media.pfizer.com/ files/products /uspi_zithromax.pdf

Programme National d’Élimination de la Filariose Lymphatique (2005). Plan Stratégique National d’Élimination de la Filariose Lymphatique 2005-2009.

Programme National de Lutte contre la Cécité (2005). Plan Stratégique National d’Élimination du Trachome 2005-2009.

**12. APPENDICES**

There are two types of appendices: general information and data gathering tools.

**12.1 Appendices A, B, C, H, I**

These appendices present the general information needed to understand this study.

1. Appendix A: Study Design
2. Appendix B: Study Flowchart
3. Appendix C: Background information on filariasis and trachoma
4. Appendix H: Summary of Product Characteristics for Azithromycin
5. Appendix I: Summary of Product Characteristics for Ivermectin and albendazole

**12.2** **Appendices D, E, F, G, J, K**

These are the tools used for gathering data. The treatment register (Appendix J) and

height poles (Appendix K) however are not included in the subject’s file.

***12.2.1 Subject’s file***

Each subject included in the study should have a file with the following elements:

1. Informed consent or assent form (Appendix D1),
2. Inclusion card (Appendix D2),
3. Questionnaire (Appendix D3),
4. Concomitant treatment and adverse events observation form (Appendix D4),
5. Clinical examination form (Appendix E),
6. Evacuation referral form (Appendix F),
7. Adverse events notification form (Appendix G).

***12.2.2 Chronology for filling the data gathering tools***

The questionnaire and other appendices should be filled in sequence:

- Appendices D1, D2 and E and sections I and II of Appendix D3 should be filled at the time of the first medical examination, on the first day of therapy.
- Section III of Appendix D3 should be filled at the time the medicine is administered.
- Section IV and Appendices D4, F, G and J should be filled during the surveillance phase, when effects are notified or at the time of the clinical evaluation.

**APPENDIX A: Study Design – Administration/Procedures**

| Randomize villages into treatment A or treatment B | | | Dosages: According to size   - Azithromycin (1 g = up to 4 tablets) - Ivermectin (12 mg = up to 4 tablets) - Albendazole (400 mg = 1 tablet each)   Adverse Event reporting:  **Day 0**: Following drug intake, subjects will be asked to report any adverse effects to the investigators at the health center or at the CSCOM, and to continue to report such effects until Day 7. They will be asked to come back on Day 8 for an evaluation.  **Day 8**: Subjects in Group A and Group B will be asked if they experienced any adverse effects following drugs intake. Questions about specific signs and symptoms will be asked and the answers will be recorded on individual questionnaires. For the control village, the next treatment (azithromycin) is administered following a clinical examination. Adverse effects are reported up to **Day 14**. On **Day 15**, subjects come back for an evaluation.  **Day 15**: Evaluation of adverse effects in group A and group B. |
| --- | --- | --- | --- |
|  | Village A  (Control group) | Village B  (Triple therapy) |
| Day 0 | **Ivermectin +**  **Albendazole** | **Azithromycin**  **+Ivermectin**  **+Albendazole** |
| Day 0 to  Day 7 | Report adverse effects | |
| Day 8 | Evaluation: Physical Health Inquiry | |
| Day 8 | **Azithromycin** |  |
| Day 8 to  Day 14 | Report adverse effects for group A and group B | |
| Day 15 | Evaluation: Physical Health Inquiry for group A and group B | |

**ANNEX B: Study Flowchart**

Staff Education/Training Sessions

**NO**

Local Mass Media Information Campaign

Door to door visits to households to conduct a census of subjects

**Authorization from Ethics Committee and Ministry of Health**

YES

Implementation of study at CSCOM or health station level

**Villages A (Control)**

**(Ivermectin+albendazole )**

Report adverse events

Report adverse events

Report adverse events

**Evaluation**: Physical health inquiry, then treatment (**Azithromycin)**

Report adverse events

**Evaluation**: Physical health inquiry

Excluded: Receive standard treatment standard

Consent?

Treatment: Doses according to size

**Villages B (Test)**

(**Tritherapy: AZI+IVM+ALB)**

**Evaluation**: Physical health inquiry: reassure the subject and encourage him to continue to report adverse events

1-7

D8

9-14

D15

D0

**STUDY FLOWCHART**

**Evaluation**: Physical health inquiry

Report results

**ANNEX C: Background information on filariasis & trachoma**

Background information on filariasis, trachoma and control strategies according to WHO standards and in Mali

## 1. Lymphatic filariasis

## According to WHO standards, filariasis represents a public health event when its prevalence in a community exceeds 1 %. The control strategy recommended by WHO has two components: interrupting the transmission and treating already affected individuals and, if the prevalence of the disease is greater than 1 % in a given district, the WHO guidelines recommend mass drug administration (MDA) with albendazole and ivermectin throughout the region. If the prevalence is lower than 1 %, individual treatment is recommended and epidemiological surveillance strengthened. In Mali, the lymphatic filariasis elimination program provides for drug access to all the members of an endemic community and interrupting disease transmission in the entire community by minimizing the reservoir of infection in the blood. In addition to MDA, the program includes surgery of hydrocele, lymphoedema treatment and the use of mosquito nets impregnated with insecticide.

## 2. Trachoma

The trachoma control strategy recommended by WHO through the GET 2020 program (Global Eradication of Trachoma) is based on the SAFE strategy (S: Surgery of trichiasis, A: Antibiotics, F: Facial Cleanliness, E: Environmental improvement). In the case of active trachoma (trachomatous inflammation – follicular [TF]/trachomatous inflammation – intense [TI]), the WHO strategy is as follows: if the prevalence in the district is greater than 10% in children aged 1 to 9, an annual MDA with azithromycin over 3 years is prescribed as one of the main tools for eliminating this disease. If the prevalence of active trachoma in children aged 1 to 9 remains greater than 10% following three azithromycin MDAs, mass treatment of the entire population is recommended. If the prevalence of active trachoma in children aged 1 to 9 is between 5 and 10%, the treatment of endemic communities is recommended. If the prevalence of active trachoma in children aged 1 to 9 is lower than 5 %, individual treatment is desirable. The object of MDAs is to treat all the members of a community while containing the transmission of active trachoma within the community. In addition to MDAs, the trachoma elimination program includes surgery for persons with trachomatous trichiasis (TT), environmental changes (e.g. construction of latrines and provision of clean water) and health education to improve hygiene.

## 3. Background information on the integrated treatment of NTDs in Mali

Treatment occurs over a minimum of 5 weeks with three interventions as indicated in the following table, in lieu of isolated campaigns.

| Week 1 | Week 2 | Week 3 | Week 4 | Week 5 |
| --- | --- | --- | --- | --- |
| AZI + Tetra 1 % |  | *IVM* + ALB |  | PZQ |
|  | PAUSE |  | PAUSE |  |

**AZI= Azithromycin (250 mg and suspension), IVM= Ivermectin (3 mg), ALB= Albendazole (400 mg), PZQ= Praziquentel (600 mg). Tetra 1% = Tetracycline ointment 1%**

**ANNEX D1: Consent and Assent Form**


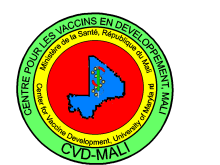


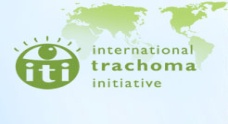


Name of investigator: …………………….. Name of supervisor: ……………………….

**Identification of study subject**:

Health Area……………Village………code V. /…/…Municipality…………… District:…….......... Identification Number: */__/__/__/__/* __/__/ __/__/

Study subject: Name and surname ……………………….Outsider: Yes/__ / No/__/

For the purposes of this consent form, “you” refers to “you and your child”**.**

*We invite you to participate in a research study conducted by ITI /Pfizer,FMPOS,CNAM,CVD. It is important that you understand the general principles that apply to everyone participating in this study. This study focuses primarily on the integrated treatment of lymphatic filariasis and trachoma.*

*Lymphatic filariasis, trachoma, onchocerciasis, and intestinal worms are endemic diseases in your community. These diseases can affect anyone, without exception. They are transmitted by insects (mosquitoes and flies), damp soil and an unsanitary environment. People can become infected without knowing it.*

*In order to control effectively these diseases, International Trachoma Initiative (ITI) in collaboration with the Centre pour le Développement des Vaccins du Mali (CVD), the School of medicine, pharmacy and odontostomatology in Mali ask you to participate in this study to better understand these diseases and better treat them, at a lower cost, in your community and your village. The researchers have been authorized by the Ministry of Health, the Direction Nationale de la Santé (DNS), the Direction Régionale de la Santé (DRS) in Sikasso, and the Centres de Santé de Référence (CSREF) in Bougouni and Kolondiéba.*

*In fact, for several years, in your region:*

- *the National Program to Eliminate Lymphatic Filariasis has organized treatment campaigns combining ivermectin and albendazole in order to control lymphatic filariasis;*
- *the National Program to Control Blindness has conducted a treatment with azithromycin tablets and oral suspension, and tétracycline 1% ointment in the villages to control trachoma;*
- *finally, albendazole (a drug used to control intestinal worms) was associated several times with vitamin A during the National Nutrition Weeks (SIAN, Semaines d’Intensification Alimentaire et Nutritionnelle) and praziquantel during the campaigns to control schistosomiasis.*

*To enhance the treatments efficiency at the global level, researches have shown that the association of ivermectin and albendazole is safe and efficient to treat lymphatic filariasis, onchocerciasis, and intestinal worms. Consequently, a study undertaken in 2006 in the United Stated regarding the three drugs where azithromycin +ivermectin+albendazole were given at the same time to healthy subjects; no serious adverse effects were observed during this trial. Our study aims to test the possibility of treating persons who suffer from or are at risk of contracting these diseases by administering at the same time AZITHROMICIN, ivermectin and ALBENDAZOLE, in order to identify the tolerance to these drugs. We want to be certain there are no major adverse effects linked to mass treatment with the three drugs in the community. This information will help us understand the treatment efficiency and safety. Our medical team will conduct a confidential physical examination to check for any potential health problem and indicate if you are eligible for the study.*

*We will ask you to participate in this study, but first you can ask us questions regarding the study. Your participation is voluntary. There will be no cost if you participate. The basic information collected during the study will remain confidential and will not be published. If the results are published, the subjects’ identity will be kept confidential. By signing a written informed consent form, you agree to have your medical history accessed within the limits authorized by the law.*

*As for the impact of the treatment, there are no direct benefits for healthy participants; however if you suffer from a disease, you will be treated with medicines. It is possible that your body will react with some effects that you will have to report. Our teams will take care of these adverse events. Your participation can help us better understand the safety of this co-administration and the scaling up of the drugs distribution will stop the transmission of lymphatic filariasis, trachoma, onchocerciasis, intestinal worms in the Sikasso region and throughout the country. We hope that 3,000 people will be willing to participate in this study. The information collected and the observations obtained will be of high value for the community and humanity as a whole.*

*You are not obliged to participate in this study, we ask you to participate and you can withdraw at any time. If you choose to participate, please check the following exclusion criteria:*

***Not included in this study****: children under age 5, pregnant women or women breastfeeding their baby, people suffering from severe illness or handicapping chronic disease, subjects being treated with drugs not compatible with the study drugs, persons who are allergic to the study drugs, persons not residing in the village or who will not be present during the two weeks of this study.*

*You can also stop your participation to this study at any time, simply by talking to the leader of the investigation team or to any other investigator in this study.*

*Few risks are associated with the proposed treatment. The intake at the same time of these drugs can result in headaches, itchiness, gastric disorders, and other problems that you will need to report immediately to the medical station. You will be examined and treated if necessary, and advised.*

*After the medicine intake, the surveillance of adverse effects will continue for two weeks. You will be examined at least twice during this surveillance phase and you will have the opportunity to visit the health agents who will offer advice and report any complaints you may have after the treatment, they will treat you if necessary.*

*You will receive a piece of Koulikoro soap to thank you for the time spent during the study.*

*Did the explanations help you understand what we want to achieve and what is expected of you during this study? Yes /__ / No /__ /*

*Do you have any questions regarding your participation in this study? Yes/__ / No/__ /*

***Consent***

*"Do you agree to participate and answer some questions?"Yes/__ / No/__ /*

**Assent**

**_** *PARENTS /GUARDIANS. "Given the fact that your child is too young to make a decision, we ask you to decide in his/her place. Do you agree to answer a few questions regarding your child?”*

*Yes/__ / /No /__ /*

*If you have any questions or concerns, you can talk to a member of our team or you can call Pr Samba Sow ………………. Dr. Antandou Telly……………………………Moussa Hama Sankaré……………*

*Dr Yaya Coulibaly……………..*

*If you agree to participate in this study, please sign or affix your fingerprint at the bottom of this page.*

***______________________________________***

***Signature, Fingerprint (Adult) Date Name_____________________***

**Signature or Fingerprint (Child ___ years old) Date Name_____________**

*If parents are illiterate, a literate witness must be present and sign:*

**Witness’ Signature: Date Name___________________**

**Investigator’s Signature Date Name_____________**

**ANNEX** D2: Study Inclusion Card

| **Inclusion card for study on co-administration of azithromycin+ivermectin+albendazole** | **Inclusion card for study on co-administration of azithromycin+ivermectin+albendazole** |
| --- | --- |
| Sikasso Region **ID N° : /_/_/_/_/_/_/_/_/**  Cercle: Bougouni/_/Kolondieba/_/  Municipality: ……………………………  Health Area: ………………………….  Village: ……………………………  Included respondent name and surname ………………………………………  Name of guardian (*for children under 18 years of age)*  …………………………………………..  Relation to the child: Father/_/Mother/_/Uncle/_/Aunt/_/ Brother/_/ Sister/_/ Cousin/_/ Grand-father(mother)/_/Other………….  Acceptance Date /_/_/_/_/2008  **Signature or left index finger:**  Respondent  Guardian | Sikasso Region **ID N°:/_ /_/_/_/_/_/_/_/**  Cercle: Bougouni/_/Kolondieba/_/  Municipality:………………………………  Health Area: ………………………….  Village: ……………………………  Included respondent name and surname …………………………………  Name of guardian (*for children under 18 years of age)*  …………………………………………..  Relation to the child: Father/_/Mother/_/Uncle/_/Aunt/_/ Brother/_/ Sister/_/ Cousin/_/ Grand-father(mother)/_/Other…………  Acceptance Date /_/_/_/_/2008  **Signature or left index finger:**  Respondent  Guardian |

**Study Exclusion Card**

| **Exclusion card for study on co-administration of azithromycin+ivermectin+albendazole** | **Exclusion card for study on co-administration of azithromycin+ivermectin+albendazole** |
| --- | --- |
| Sikasso Region **ID N° :/_/_/_/_/_/_/_/_/**  Cercle: Bougouni/_/Kolondiéba/_/  Municipality: ……………………………  Health Area: ……………………….  Village: ……………………………  Excluded respondent name and surname ……………………………  Name of guardian (*for children under 18 years of age)*  …………………………………………..  Relation to the child: Father/_/Mother/_/Uncle/_/Aunt/_/ Brother/_/ Sister/_/ Cousin/_/ Grand-father(mother)/_/ Witness/__/Other………….  Date of refusal to participate /_/_/_/_/2009  **Signature or left index finger:**  Respondent  Guardian / | Sikasso Region **ID N°:/_ /_/_/_/_/_/_/_/**  Cercle: Bougouni/_/Kolondiéba/_/  Municipality: ……………………………  Health Area: ………………………….  Village: ……………………………  Excluded respondent name and surname ……………………………  Name of guardian (*for children under 18 years of age)*  …………………………………………..  Relation to the child: Father/_/Mother/_/Uncle/_/Aunt/_/ Brother/_/ Sister/_/ Cousin(e)/_/ Grand-father(mother)/_/ Witness/__/Other…………  Date of refusal to participate /_/_/_/_/2009  **Signature or left index finger:**  Respondent  Guardian |

## ANNEX D3: Questionnaire

**Safety study on co-administration of ivermectin, albendazole and azithromycin in the Sikasso region of Mali**

**International Trachoma Initiative (ITI) in collaboration**

**with CVD/CNAM/FMPOS**

Name of investigator: ………………………… Name of supervisor: ……………………

**I-SUBJECT IDENTIFICATION:**

Q001 Health area……………Q002 Village:………code V./…/….Q003 Municipality…………… Q004 District:…………….

Q005 Consent Card Number:*/__/__/__/__/* Q006 Survey Sheet Number:*/__/__/__/__/*

Q007 Subject: Surname and first name………………………. Foreigner: Yes/__ / No/__/

Q009 Age:*/__/__/ Q010* *Sex/__/ Q011* TA:…/… Q012 Temperature:*/__/__/*

**II- Interview and examination prior to treatment:**

**Table 1:** Complaints prior to treatment on D0

| Events prior to treatment: Complaints | | Complaints  existing prior to treatment | | Code  Do not complete |
| --- | --- | --- | --- | --- |
|  | **Symptoms/Signs** | **Yes** | **No** |  |
| Q013 | Headaches |  |  |  |
| Q014 | Fever |  |  |  |
| Q015 | Tinnitus |  |  |  |
| Q016 | Deafness |  |  |  |
| Q017 | Jaundice |  |  |  |
| Q018 | Weakness |  |  |  |
| Q019 | Fatigue |  |  |  |
| Q020 | Nausea |  |  |  |
| Q021 | Vomiting |  |  |  |
| Q022 | Diarrhea |  |  |  |
| Q023 | Abdominal pain |  |  |  |
| Q024 | Flatulence/dyspepsia |  |  |  |
| Q025 | Constipation |  |  |  |
| Q026 | Joint/muscular pain |  |  |  |
| Q027 | Swelling of (upper/lower) limbs |  |  |  |
| Q028 | Swelling of eyelids/abnormal feeling in the eyes |  |  |  |
| Q029 | Rash/plaque |  |  |  |
| Q030 | Scrotal reaction |  |  |  |
| Q031 | Skin nodules |  |  |  |
| Q032 | Worm expulsion |  |  |  |
| Q033 | Haematuria |  |  |  |
| Q034 | Adenopathy |  |  |  |
| Q035 | Palpitation/tachycardia |  |  |  |
| Q036 | Orthostatic hypotension |  |  |  |
| Q037 | Others |  |  |  |
| Q037.99 | To be specified……………………. |  |  |  |

*After completing the table, the investigator will answer the following question (the answer will be yes if one complaint is ticked in the above table):*

Q038: Did the examined subject have complaints prior to the treatment? YES/__/ NO/__/

Q039: Main complaint:………………………………………

Q040: How would you rate this complaint? **0 = None; 1 = minor; 2 = moderate**

Q041: Is the subject being examined for filariasis? YES/___/ NO/___/

**Table 2: Diagnostic of complaints from lymphatic filariasis**

|  | **Result of final examination for filariasis** | | |
| --- | --- | --- | --- |
|  |  | **YES** | **NO** |
| Q042 | Elephantiasis |  |  |
| Q043 | Hydrocele |  |  |
| Q044 | Lymphoedema |  |  |
| Q045 | Other signs of filariasis |  |  |
| Q045.99 | To be specified |  |  |

Q046: Are eyelids being examined for trachoma?: YES/___/ NO/__/

Result of eyelid examination:

TF: Follicular Trachoma TI: Intense Trachoma TS: Scarring Trachoma

CO: Corneal Opacity TT: Trachomatous Trichiasis

Q047: OD (right eye): Normal/__/ TF/__/ TT/__/ TS/__/ CO/__/ Other/__/

To be specified:

Q048: OD (left eye): Normal/__/ TF/__/ TT/__/ TS/__/ CO/__/ Other/__/

To be specified:

**Table 3:** Final result of physical examination for trachoma

| **Final result for trachoma** | | | |
| --- | --- | --- | --- |
|  |  | **Yes** | **No** |
| Q049 | TF |  |  |
| Q050 | TI |  |  |
| Q051 | TT |  |  |
| Q052 | TS |  |  |
| Q053 | CO |  |  |
| Q054 | Trachoma |  |  |

*(Thank the subject for his participation and accompany him to the treatment station with his file)*

**III-CO-ADMINISTRATION OF DRUGS**

Q056 Height:……m…….cm Q057 Weight:/_/_/ kg

Number of tablets: IVM:/__/ AZI Tablets:/__/ ALB:/__/ Date:… /…/….

Hours:……H… mn

Q058 Did the subject swallow the drug in your presence? YES/___/ NO/__/

Q059 Timing of drug administration in relation to meals: Empty stomach:/___/ After a meal:/__/

Q060 Did the subject regurgitate the drugs YES/__/ NO/__/

Q061 Were the drugs taken again after regurgitating: YES/__/ NO/__/

Q062 Treatment validated YES/__/ NO/___/

(Thank the subject for his participation and keep his file for surveillance)

**IV- PASSIVE SURVEILLANCE AND ACTIVE SURVEILLANCE**

**4.1 : Adverse Effects (non serious) Pharmacovigilance from D0 to D8**

**Note: This section is to be completed following the treatment from D0 to D8**

Q063: Dates of first filling-in for surveillance purposes:…../……/……/

Q064: Which day (please circle): D0 D2 D3 D4 D5 D6 D7 D8

Q065: Place of first filling-in?: CSCOM/__/ Survey site/__/ Home/__/ Other/___/

Q065.99: To be specified…………………………………………………………….

Q066: Did you take the drugs? YES/__/ NO/__/

Q067: Did you regurgitate the drugs? YES/__/ NO/__/

Q068: When did you take the drugs (circle the day)?: D0 D2 D3 D4 D5 D6 D7

Q069: When did you take the drugs in relation to meals? Empty stomach/__/ After a meal/__/ Other:/__/

Q070: How did you feel after taking the drugs?

Fine (better than before):/__/ Same as before:/__/ Bad:/__/ Do not know:/__/

*If* ***Fine (better than before),*** *review the table of complaints (Table 4) to describe their evolution after the triple therapy from* ***Q071 to Q104*** *and ignore the table of side-effects(Tables 5.1 and 5.2, Q105 to Q131))and* ***go to Question Q104.***

*If* ***Bad****, first specify the side effects (****Q105 to Q131)*** *and the days they occurred before completing the table of complaints (Table 4).*

*(If treatment, please indicate in the table of treatments the drugs taken.)*

**For complaints or events prior to the treatment:** Write the answer in each box below, using the following code :

- 0 = None: the complaint has gone
- 1 = Improved: the complaint is significantly less than before the treatment
- 2 = Ongoing: the complaint is the same as before the treatment
- 3 = Exacerbated: the complaint increased after the treatment

If severe and exacerbated, i.e. the subject cannot work and needs medical assistance, please complete the table of severe adverse effects and the notification slip for serious adverse events:

**Table 4.1:** Evaluation of complaints following treatment from D0 to D8

| **Fine (feeling of satisfaction)** | |  |  |
| --- | --- | --- | --- |
| **Events existing before the treatment** | | **Complaints**  **0 = None,  1 = Improved**  **2 = Ongoing**  **3 = Exacerbated** | **Code** |
|  | **Symptoms/Signs** | After the treatment from D0 to D8 |  |
| Q071 | Headaches |  |  |
| Q072 | Fever |  |  |
| Q073 | Tinnitus |  |  |
| Q074 | Deafness |  |  |
| Q075 | Jaundice |  |  |
| Q076 | Weakness |  |  |
| Q077 | Fatigue |  |  |
| Q078 | Nausea |  |  |
| Q079 | Vomiting |  |  |
| Q080 | Diarrhea |  |  |
| Q081 | Abdominal pain |  |  |
| Q082 | Flatulence/dyspepsia |  |  |
| Q083 | Constipation |  |  |
| Q084 | Joint/muscular pain |  |  |
| Q085 | Swelling of (upper/lower) limbs |  |  |
| Q086 | Swelling of eyelids/abnormal feeling in the eyes |  |  |
| Q087 | Rash/plaque |  |  |
| Q088 | Scrotal reaction |  |  |
| Q089 | Skin nodules |  |  |
| Q090 | Worm expulsion |  |  |
| Q091 | Haematuria |  |  |
| Q096 | Adenopathy |  |  |
| Q097 | Palpitation/tachycardia |  |  |
| Q098 | Orthostatic hypotension |  |  |
| Q099 | Others to be specified…………………… |  |  |
| Q100b |  |  |  |
| Q101c |  |  |  |

*After completing the table, the investigator will answer the following question (the answer will be yes if one complaint is ticked 3 =* ***exacerbated*** *in the above table):*

Q102: Did the interviewed/examined subject experience complaints after taking the 3 drugs? YES/___/ NO/__/ If yes,

Q103: Which one?

Q104 How would you qualify your main complaint ………………………. after taking the 3 drugs/___/  **0=None 1 = Improved 2 = Ongoing 3 = Exacerbated**

**For adverse events:** Write the response in each box below, using the following code:

- - **0 = did not feel sick:** no adverse effect
  - **1 = minor:** easily tolerated, does not interfere with daily activity
  - **2 = moderate :** uncomfortable enough to interfere with daily activity
  - **3 = severe :** precludes daily activity

**Table 5.1:** Adverse effects experienced after the treatment from D0 to D8

|  | **Adverse Events** | **BAD (malaise)** | | | | | | | | |  |
| --- | --- | --- | --- | --- | --- | --- | --- | --- | --- | --- | --- |
|  | **Symptoms/signs** | **Days when side effects appeared D0 to D8**  **0 = did not feel sick, 1 = minor, 2 = moderate,**  **3 = major** | | | | | | | **Evaluation** | **Code** | |
|  |  | **D0** | **D1** | **D2** | **D3** | **D4** | **D5** | **D8** | **D8** |  | |
| Q105 | Headaches |  |  |  |  |  |  |  |  |  | |
| Q106 | Fever |  |  |  |  |  |  |  |  |  | |
| Q107 | Tinnitus |  |  |  |  |  |  |  |  |  | |
| Q108 | Deafness |  |  |  |  |  |  |  |  |  | |
| Q109 | Jaundice |  |  |  |  |  |  |  |  |  | |
| Q110 | Weakness |  |  |  |  |  |  |  |  |  | |
| Q111 | Fatigue |  |  |  |  |  |  |  |  |  | |
| Q112 | Nausea |  |  |  |  |  |  |  |  |  | |
| Q113 | Vomiting |  |  |  |  |  |  |  |  |  | |
| Q114 | Diarrhea |  |  |  |  |  |  |  |  |  | |
| Q115 | Abdominal pain |  |  |  |  |  |  |  |  |  | |
| Q116 | Flatulence/dyspepsia |  |  |  |  |  |  |  |  |  | |
| Q117 | Constipation |  |  |  |  |  |  |  |  |  | |
| Q118 | Joint/muscular pain |  |  |  |  |  |  |  |  |  | |
| Q119 | Swelling of (upper/lower) limbs |  |  |  |  |  |  |  |  |  | |
| Q120 | Swelling of eyelids/abnormal feeling in the eyes |  |  |  |  |  |  |  |  |  | |
| Q121 | Rash/plaque |  |  |  |  |  |  |  |  |  | |
| Q122 | Scrotal reaction |  |  |  |  |  |  |  |  |  | |
| Q123 | Skin nodules |  |  |  |  |  |  |  |  |  | |
| Q124 | Worm expulsion |  |  |  |  |  |  |  |  |  | |
| Q125 | Haematuria |  |  |  |  |  |  |  |  |  | |
| Q126 | Adenopathy |  |  |  |  |  |  |  |  |  | |
| Q127 | Palpitation/tachycardia |  |  |  |  |  |  |  |  |  | |
| Q128 | Orthostatic hypotension |  |  |  |  |  |  |  |  |  | |
| Q129 | Others…………………… |  |  |  |  |  |  |  |  |  | |
| Q130 |  |  |  |  |  |  |  |  |  |  | |
| Q131 |  |  |  |  |  |  |  |  |  |  | |

**Table 5.2** will be completed during the second week of the study.

(Distribution of Azithromycin in villages belonging to group A, on the 8^th^ day)

Number of tablets: AZI tablets:/__/ Date:…/…/… Time: ….h…..mn

Q132 Did the subject swallow the drug in your presence? YES/___/ NO/__/

Q133 Timing of drug administration in relation to meals: Empty stomach:/___/ After a meal:/__/

Q134 Did the subject regurgitate the drugs YES/__/ NO/__/

Q135 Were the drugs taken again after regurgitating: YES/__/ NO/__/

Q136 Treatment validated YES/__/ NO/___/

(*Thank the subject for his participation, encourage him to continue with the study and to report any adverse effects, ask him to come back on the 15^th^ day for another medical examination even if he feels well*)

**4.2: Adverse Effect (non serious) Pharmacovigilance between D8 and D15**

*(The subject will be interviewed and examined again between D8 and D15: Review the complaints from Table 5.2 without reading them. In case of a new complaint, write them down in the appropriate box, then fill Table 4.2 (from Day 8 to D15) and check the complaints that were reported at the beginning of the study)*

**Table 4.2:** Evaluation of complaints following treatment from D8 to D15

| **Fine (feeling of satisfaction)** | |  |  |
| --- | --- | --- | --- |
| **Events existing before the treatment** | | **Complaints**  **0 = None,  1 = Improved**  **2 = Ongoing**  **3 = Exacerbated** | **Code** |
|  | **Symptoms/Signs** | After the treatment and/or evaluation on D8 |  |
| Q071b | Headaches |  |  |
| Q072b | Fever |  |  |
| Q073b | Tinnitus |  |  |
| Q074b | Deafness |  |  |
| Q075b | Jaundice |  |  |
| Q076b | Weakness |  |  |
| Q077b | Fatigue |  |  |
| Q078b | Nausea |  |  |
| Q079b | Vomiting |  |  |
| Q080b | Diarrhea |  |  |
| Q081b | Abdominal pain |  |  |
| Q082b | Flatulence/dyspepsia |  |  |
| Q083b | Constipation |  |  |
| Q084b | Joint/muscular pain |  |  |
| Q085b | Swelling of (upper/lower) limbs |  |  |
| Q086b | Swelling of eyelids/abnormal feeling in the eyes |  |  |
| Q087b | Rash/plaque |  |  |
| Q088b | Scrotal reaction |  |  |
| Q089b | Skin nodules |  |  |
| Q090b | Worm expulsion |  |  |
| Q091b | Haematuria |  |  |
| Q096b | Adenopathy |  |  |
| Q097b | Palpitation/tachycardia |  |  |
| Q098b | Orthostatic hypotension |  |  |
| Q099b | Others to be specified…………………… |  |  |
| Q100bb |  |  |  |
| Q101cb |  |  |  |

Q102b: Did the interviewed/examined subject experience complaints after taking the 3 drugs? YES/___/ NO/__/ If yes,

Q103b: Which one?

Q104b How would you qualify your main complaint ………………………. after taking the 3 drugs/___/  **0=None 1 = Improved 2 = Ongoing 3 = Exacerbated**

**Table 5.2**: Adverse Effects experienced after the treatment from **D8 to D15**

|  | **Adverse Events** | **BAD (malaise)** | | | | | | | | |  |
| --- | --- | --- | --- | --- | --- | --- | --- | --- | --- | --- | --- |
|  | **Symptoms/signs** | **Days when side effects appeared D8 to D15**  **0 = did not feel sick, 1 = minor, 2 = moderate,**  **3 = major** | | | | | | | **Evaluation** | **Code** | |
|  |  | **D8** | **D9** | **D10** | **D11** | **D12** | **D13** | **D14** | **D15** |  | |
| Q105b | Headaches |  |  |  |  |  |  |  |  |  | |
| Q106b | Fever |  |  |  |  |  |  |  |  |  | |
| Q107b | Tinnitus |  |  |  |  |  |  |  |  |  | |
| Q108b | Deafness |  |  |  |  |  |  |  |  |  | |
| Q109b | Jaundice |  |  |  |  |  |  |  |  |  | |
| Q110b | Weakness |  |  |  |  |  |  |  |  |  | |
| Q111b | Fatigue |  |  |  |  |  |  |  |  |  | |
| Q112b | Nausea |  |  |  |  |  |  |  |  |  | |
| Q113b | Vomiting |  |  |  |  |  |  |  |  |  | |
| Q114b | Diarrhea |  |  |  |  |  |  |  |  |  | |
| Q115b | Abdominal pain |  |  |  |  |  |  |  |  |  | |
| Q116b | Flatulence/dyspepsia |  |  |  |  |  |  |  |  |  | |
| Q117b | Constipation |  |  |  |  |  |  |  |  |  | |
| Q118b | Joint/muscular pain |  |  |  |  |  |  |  |  |  | |
| Q119b | Swelling of (upper/lower) limbs |  |  |  |  |  |  |  |  |  | |
| Q120b | Swelling of eyelids/abnormal feeling in the eyes |  |  |  |  |  |  |  |  |  | |
| Q121b | Rash/plaque |  |  |  |  |  |  |  |  |  | |
| Q122b | Scrotal reaction |  |  |  |  |  |  |  |  |  | |
| Q123b | Skin nodules |  |  |  |  |  |  |  |  |  | |
| Q124b | Worm expulsion |  |  |  |  |  |  |  |  |  | |
| Q125b | Haematuria |  |  |  |  |  |  |  |  |  | |
| Q126b | Adenopathy |  |  |  |  |  |  |  |  |  | |
| Q127b | Palpitation/tachycardia |  |  |  |  |  |  |  |  |  | |
| Q128b | Orthostatic hypotension |  |  |  |  |  |  |  |  |  | |
| Q129b | Others…………………… |  |  |  |  |  |  |  |  |  | |
| Q130b |  |  |  |  |  |  |  |  |  |  | |
| Q131b |  |  |  |  |  |  |  |  |  |  | |

*After completing Tables 5.1 and 5.2, the investigator will answer the following question (the answer will be yes if a* ***minor, moderate or intense*** *adverse event is ticked in the table above)*

Q151: Did the interviewed/examined subject experience an adverse event after receiving the treatment? YES/___/ NO/__/

Q137: How would you qualify the adverse events experienced by the interviewed/examined subject?/___/

**0 = did not feel sick,**

**1 = minor,**

**2 = moderate,**

**3 = severe**

Q138: Was your malaise treated? YES/___/ NO/__/

**4.3 Pharmacovigilance of Serious Adverse Effects between D0 and D8**

**Table 6.1: Serious Adverse Effects between the first day and the evaluation day (D8)**

| **CASES OF SERIOUS EFFECTS** | | | | | | | | | | | | |
| --- | --- | --- | --- | --- | --- | --- | --- | --- | --- | --- | --- | --- |
|  |  | **Severe effects** | **Days when serious side effects occurred between Day 0 and Day 8**  **0 = None, 1 = Severe, 2 = Very severe** | | | | | | | | | **Code** |
|  |  |  | **D0** | **D1** | **D2** | **D3** | **D4** | **D5** | **D6** | **D7** | **D8** |  |
| Q139 |  | Angioneurotic oedema/anaphylactic shock |  |  |  |  |  |  |  |  |  |  |
| Q140 |  | Anuresis |  |  |  |  |  |  |  |  |  |  |
| Q141 |  | Seizures |  |  |  |  |  |  |  |  |  |  |
| Q142 |  | Erythema multiforme |  |  |  |  |  |  |  |  |  |  |
| Q143 |  | Epidermal necrolysis |  |  |  |  |  |  |  |  |  |  |
| Q144 |  | Recurrent symptoms |  |  |  |  |  |  |  |  |  |  |
| Q145 |  | Stevens Johnson Syndrome |  |  |  |  |  |  |  |  |  |  |
| Q146 |  | Others to be specified…… |  |  |  |  |  |  |  |  |  |  |

**4.4 Pharmacovigilance of Serious Adverse Effects between D8 and D15**

**Table 6.2: Serious Adverse Effects between D8 and D15**

| **CASES OF SERIOUS EFFECTS** | | | | | | | | | | | |
| --- | --- | --- | --- | --- | --- | --- | --- | --- | --- | --- | --- |
|  |  | **Severe effects** | **Days when serious side effects occurred between Day 8 and Day 15**  **0 = None, 1 = Severe, 2 = Very severe** | | | | | | | | **Code** |
|  |  |  | **D8** | **D9** | **D10** | **D11** | **D12** | **D13** | **D14** | **D15** |  |
| Q139b |  | Angioneurotic oedema/anaphylactic shock |  |  |  |  |  |  |  |  |  |
| Q140b |  | Anuresis |  |  |  |  |  |  |  |  |  |
| Q141b |  | Seizures |  |  |  |  |  |  |  |  |  |
| Q142b |  | Erythema multiforme |  |  |  |  |  |  |  |  |  |
| Q143b |  | Epidermal necrolysis |  |  |  |  |  |  |  |  |  |
| Q144b |  | Recurrent symptoms |  |  |  |  |  |  |  |  |  |
| Q145b |  | Stevens Johnson Syndrome |  |  |  |  |  |  |  |  |  |
| Q146b |  | Others to be specified…… |  |  |  |  |  |  |  |  |  |

*After completing Tables 6.1 and 6.2, the investigator will answer the following question (the answer will be yes if a severe effect is ticked in the above table):*

Q147: Did the interviewed/examined subject experience a serious/severe adverse event after taking the 3 drugs? YES/___/ NO/__/

Q148: If yes, which one……………………………………………………..

**Causality assessment criteria:**

**Q149: Is the serious effect related to the therapy?** Not related/___/ Probably/___/ Unlikely/___/

**Arguments:………………………………………………………………………………….**

Q150: Remarks/Observations regarding this serious adverse effect case management:

(Note: Serious adverse effects will be treated and observed for 24 hours (maximum) by the physicians on the field. If there is no improvement, the subject will be transferred to the Centre de Référence or to the most appropriate hospital).

Thank you for your collaboration

## ANNEX D4: Fever and serious adverse events surveillance form

Name and surname……………………………….. Age…………….. Sex…………..Village…..Card Number/_/_/_/_/_/_/_/_/.  Date of onset:………………

Agent in charge of case management:…………………………………………………

Q151: If this is a potential case of malaria fever: Is the malaria rapid diagnostic test positive: YES/__/ NO/__/ **Table 8:** Fever evaluation

**Table7:** Observation of fever /___/ and other events:/___/…………………………………

| Fever | | Normal | reduced | constant | augmented |
| --- | --- | --- | --- | --- | --- |
| Q160 | Après 1H |  |  |  |  |
| Q161 | Après 2H |  |  |  |  |
| Q162 | Après 3H |  |  |  |  |
| Q163 | Après 4H |  |  |  |  |

| Events | | | | Surveillance hours after triple therapy and other treatment | | | | | | |
| --- | --- | --- | --- | --- | --- | --- | --- | --- | --- | --- |
| Fever | | 0 | | 1H | 2H | 3H | 4H | 5H | 6H | 7H |
| Q152 | Triple therapy  Yes /__/ No /__/ | Treatment  Fever: Yes /__/ No/___/ | Q154 | …… | …… | …… | …… | …… | …… | …… |
| Q153 | Concomitant1  Drugs……………………… | Temperature | Q159 | …… | …… | …… | …… | …… | …… | …… |

After filling table 9, the investigator will answer question Q164: How did the fever evolve after the triple therapy?

Q164: Codification of evolution of fever concomitant treatment: Remission /__/Persistent/__/Exacerbation/__/

Following days

**Table 8:** Malaria: /_/ Other fever /___/ Other diagnostic :………………………………………………Date of onset /__/__/__ /__/0/9

| Events | | | | Surveillance hours after triple therapy and other treatment | | | | | | |
| --- | --- | --- | --- | --- | --- | --- | --- | --- | --- | --- |
| Fever | | D0=Day of treatment | | Day1 | Day2 | Day3 | Day4 | Day5 | Day 6 | Day7 |
| Q165 | Triple therapy  Yes/__/  No/__/ | Concomitant treatment 2  Yes /__ / No /___/  Drugs………… … | | …… | …… | …… | …… | …… | …… | …… |
| Q166 | ……… | Temperature  ………….. | Morning | …… | …… | …… | …… | …… | …… | …… |
|  |  |  | Evening |  |  |  |  |  |  |  |
|  |  | Bp……….. | Morning |  |  |  |  |  |  |  |
|  |  |  | Evening |  |  |  |  |  |  |  |
|  |  | Pulse…….. | Morning |  |  |  |  |  |  |  |
|  |  |  | Evening |  |  |  |  |  |  |  |
| Observations: Precisions concerning treatment and evolution…………………………………. | | | |  |  |  |  |  |  |  |

**Date of remission of adverse effect:**/__/__/__/__/2009 Q167**: Serious adverse effect related to therapy: No link**/__/**Probable/__/Unlikely**

**ANNEX E: Clinical examination card**

**Card Number/ID Number** /_/_ /_/_/_ /_/_/_/

**1. List of medical history events to research**:

| **MEDICAL HISTORY** | **YES** | **NO** |
| --- | --- | --- |
| 1. Convulsions |  |  |
| 1. Icterus |  |  |
| 1. Allergy |  |  |
| 1. Vaginitis |  |  |
| 1. Deafness |  |  |
| 1. Behavior disorder (aggressiveness or depression) |  |  |
| 1. Epilepsy |  |  |
| 1. Lymphadenopathy |  |  |
| 1. Medicine intake: antiacids, antiretrovirals, cardio-vascular or headache medicines, anticoagulants |  |  |
| 1. Others |  |  |
| 1. If others, please specify: |  |  |

**2. List of contraindications to look for**:

| **Contraindications** | **YES** | **NO** |
| --- | --- | --- |
| 1. Pregnancy |  |  |
| 1. Breastfeeding |  |  |
| 1. Children less than 5 years of age (≤90cm) |  |  |
| 1. Allergy to one of the study drugs: azithromycin, Ivermectin, Abendazole |  |  |
| 1. Medicine intake: antiacids containing aluminium or magnesium, nelfinar, ergot derivatives |  |  |
| 1. Serious illness (systemic edema, severe dyspnea, etc.) |  |  |
| 1. Others……………………….. |  |  |
| 1. If others, please specify: |  |  |
| **DECISION REGARDING INCLUSION IN THE STUDY** |  |  |

## Concomitant Treatments: 0: Not administered 1: Administered

| Concomitant Treatments | Day0 | Day 1 | Day2 | Day3 | Day4 | Day5 | Day6 | Day7 | Day8 | Day9 | Day10 | Day11 | Day12 | Day13 | Day14 | Day15 | code |
| --- | --- | --- | --- | --- | --- | --- | --- | --- | --- | --- | --- | --- | --- | --- | --- | --- | --- |
| Antalgic treatment: paracétamol |  |  |  |  |  |  |  |  |  |  |  |  |  |  |  |  |  |
| Anti-inflammatory treatment: diclofenac |  |  |  |  |  |  |  |  |  |  |  |  |  |  |  |  |  |
| Anti-malarial treatment: artemisin and quinine compounds |  |  |  |  |  |  |  |  |  |  |  |  |  |  |  |  |  |
| Antibiotics treatment: all categories |  |  |  |  |  |  |  |  |  |  |  |  |  |  |  |  |  |
| Local treatment (eyes, lymphoedema, skin, etc.) |  |  |  |  |  |  |  |  |  |  |  |  |  |  |  |  |  |
| Solutes |  |  |  |  |  |  |  |  |  |  |  |  |  |  |  |  |  |
| Corticoids |  |  |  |  |  |  |  |  |  |  |  |  |  |  |  |  |  |
| Cardio-stimulants |  |  |  |  |  |  |  |  |  |  |  |  |  |  |  |  |  |
| Anxiolytics |  |  |  |  |  |  |  |  |  |  |  |  |  |  |  |  |  |
| Antihistaminics |  |  |  |  |  |  |  |  |  |  |  |  |  |  |  |  |  |

Concomitant Treatment: Number of days/__/__/ days Evolution: Favorable: YES/__/ NO/__/

Sikasso Region Cercle: ……………………

**ANNEX F: Referral/Evacuation Form**

Patient’s Name and Surname………………………………. Card Number/__/__/__/__/__/__/__/__/

Age…………….. Sex…………..Village…………………..Municipality…………………

Health Area ………………………………….

Study treatment: Triple Therapy/_/ Standard Treatment /_/ Other /_/ To be specified………

Diagnostic of adverse effect: …………………………………………………………

Date of onset (DD/MM/YY) /_/_/_/_/0/9/ Time of onset/_/_/hour/_/_/mn

The agent in charge of case management who made the diagnostic and decided the referral or evacuation…………………………Title……………………. …….Signature……………

Evacuation /_/ Referral/_/ Date (DD/MM/YY) :/_/_/_/_/0/9/ Time of departure/_/_/hour/_/_/mn

Treatment undertaken at study site…………………………………………………………

……………………………………………………………………………………………………

……………………………………………………………………………………………………

Evacuation/Referral means…………………………………………………………………

Referral/Evacuation site ………………………………………………………………………

Name of agent in charge of reception at the Centre de Référence/Hospital …………………………

Title …………………….Signature……………………….

Arrival Date /_/_/_/_/0/9/ Arrival Time/_/_/hour/_/_/mn

Treatment undertaken at the reference site……………………………………………

……………………………………………………………………………………………………

……………………………………………………………………………………………………

Confirmation of diagnostic/adverse event…………………………………………

**Evaluation criteria according to study therapy:**

Adverse event related to therapy: No relation /___ / Probable /___/ Unlikely /__/

Arguments:………………………………………………………………………………….

………………………………………………………………………………………………..

**Evaluation by:**

Investigator/__/ Name

Main Investigator/__/ Name

DSMB Investigator /__/ Name

Others/__/ Specify

Date ……………………………………………………………Date………………………….

Study team leader Reference site manager

Name, surname, and signature Name, surname, and signature

**APPENDIX G**

Ministère de la santé République du Mali

************* Un Peuple Un But Une Foi

Region _____________________________ Cercle ___________________________

**Centre _________________________________ Telephone___________________________**

**Serious adverse events following medicine intake**

**Notification Form**

**1. Patient’s Identification**  Inclusion Card Number:I_I__I__I__I__I__I__I__I

**Age_____________________Sex_______________Weight________________Height_______**

**Occupation______________________________________Residence_____________________**

**Marital Status**

**Married /__/ Single /__/ Number of dependent children /__/ Others/ __/ Specify**

**2. Medical, serology and psychiatric, etc. history:_____________________________________________**

**Abuse or addiction history____________________________________________**

**_____________________________________________________________________________**

**Known allergies:____________________________________________________________**

**3.History**

|  |
| --- |

**4. Clinical description**

|  |
| --- |

(Be as precise as possible)

| PATIENT’S HEALTH STATUS | EVOLUTION |
| --- | --- |
| Hospitalization or prolongation of hospitalization/__/  Permanent disability or handicap /__/  Guarded prognosis /__/  Death /__/ | Recovery /__/  Death /__/  Sequela /__/  Number of lost working days /__/  Others (specify) /__/……………………… |

**5. Medicines**

| Summary of Product Characteristics (SPC) | | CONFUSION | |
| --- | --- | --- | --- |
| Form/Dosage |  | Form/Dosage |  |
| Batch Number |  | Batch Number |  |
| Expiry Date |  | Expiry Date |  |
| Posology |  | Posology |  |
| Method of administration |  | Method of administration |  |
| Treatment duration |  | Treatment duration |  |
| Indication |  | Indication |  |
| Manufacturer |  | Manufacturer |  |
| Expected reactions |  | Expected reactions |  |

**6. Procurement Method**

Prescription (indicate reason):…………………………………………………………………

Self-medication:…………………………………………………………………………………

Others (specify):………………………………………………………………………………

**7. Research, dosage of products eventually taken**

| Date | Researched product | Biological sample | Result |
| --- | --- | --- | --- |
|  |  |  |  |
|  |  |  |  |

**8. Reaction** Treatment

| Reaction Treatment |  |
| --- | --- |
| Form /Dosage |  |
| Posology |  |
| Method of administration |  |
| Treatment duration |  |
|  |  |
|  |  |

## 9. Medicines associated with study treatment

| Designation | Form/ Dosage | Targeted pathology (diagnostic) | Method of administration | Relations between the targeted pathology and the study drugs adverse effects | Posology | Treatment duration | Observation |
| --- | --- | --- | --- | --- | --- | --- | --- |
|  |  |  |  |  |  |  |  |
|  |  |  |  |  |  |  |  |

**10. Conclusion**

**Signatures**

Declarant Investigator (sworn agent)

Name, first name, and address Name, surname, address, and seal

………………………………… ……………………………………...

………………………………… ……………………………………….

…………………………………. …………………………………………

## APPENDIX H: Azithromycin Technical Note

*Summary of Product Characteristics (SPC):*

**PRESENTATION** OF **AZITHROMYCIN:**

**Azithromycin: Zithromax***: Sold in 500 mg and 250 mg tablets and in 1,200 mg bottles for oral suspension.

**ABSORPTION-DISTRIBUTION:**

- Azithromycin is quickly absorbed following oral administration.
- The tablet absorption is not influenced by food intake.
- Peak plasma concentrations are attained 2-3 hours after intake.
- In pharmacokinetic studies it has been demonstrated that the concentrations of azithromycin measured in tissues are noticeably higher (as much as 50 times) than those measured in plasma, which indicates that the agent strongly binds to tissues. It has also been demonstrated that an administration of 1.5 g of azithromycin for 3 or 5 days is similar.
- The terminal plasma elimination half-life closely reflects the elimination half-life tissues of 2-4 days.
- Azithromycin is widely distributed throughout the body: after a single intake of 500 mg, observed concentrations in target tissues exceed CMI90 of germs most often responsible for respiratory or prostatic infections, or tonsillitis. Macrolides penetrate and accumulate in the phagocytes (neutrophils, monocytes, peritoneal and alveolar macrophages).

Intraphagocytic concentrations are high in humans. These properties explain the effect of azithromycin on intracellular bacteria.

- In experimental infections, higher quantities of azithromycin have been found in phagocytes. It has also been established that during active phagocytes higher concentrations of azithromycin are released from inactive phagocytes. In animal models, this results in high concentrations of azithromycin being delivered to the site of infection.
- Azithromycin is bound in plasma protein at a rate of 20%.

**ELIMINATION:**
High concentrations of unchanged azithromycin have been found in bile and urines.

The liver is the main biotransformation agent of azithromycin, via N-demethylation. Azithromycin is eliminated mainly by the bile.

There is also a minor urinary elimination of the product. During a 5 days treatment, the product could be traced in urines after 24 hours and up to three weeks after intake.

**PRECAUTIONS FOR USE:**

- No dose adjustment is necessary in patients with mild renal impairment (GFR > 40 ml/mn). In the absence of data for patients with a GFR < 40 ml/mn, caution should be used when azithromycin is prescribed.
- In patients with moderate (class A) to mild (class B) renal impairment, there was no significant change in the serumal kinetic of azithromycin when compared to those of patients with normal renal functions.
  In such patients, there is an increase in urinary elimination, probably to compensate the reduced GFR. This is why no dosage adjustment is recommended in patients suffering from mild to moderate renal impairment.
  Since azithromycin is metabolized in the liver, the drug is not recommended for patients suffering from severe liver disease or severe cholestasis.
- As with erythromycin and other macrolides, rare *serious allergic reactions,* including angioneurotic edema and anaphylaxis have been reported.

Some of these reactions have resulted in recurrent symptoms and required a longer period of observation and treatment.

- *Because azithromycin contains lactose*, it is contra-indicated in case of congenital galactosemia, syndrome of glucose malabsorption, galactose, and lactase deficit.

**PREGNANCY AND LACTATION**

**Pregnancy**

*-1^st^ trimester*:

It is preferable, as a precaution, not to use azithromycin during the first trimester of pregnancy. Even though animal studies on rodents do not demonstrate any malformation effect, clinical data is insufficient.
-*From 2d trimester*:

Because of the expected benefice, azithromycin can be used starting in the second trimester of pregnancy, if necessary. Even though clinical data is limited, it is reassuring regarding treatment after the first trimester.

**Lactation**
There is insufficient data regarding excretion of azithromycin in human breast.

The safety of azithromycin in lactating women is not established, therefore it should be prescribed only if expected benefits outweigh potential risks.

**INDICATIONS**: Treatment of active trachoma

During the current campaign, 250 mg tablets packaged in boxes of 30 tablets and bottles of 1,200 mg oral suspension will be used.

**POSOLOGY**

The treatment is given in one single dose with water, preferably on an empty stomach. However, food has no influence on the absorption of the drug.

A 12 months interval is recommended between two campaigns.

During a mass treatment campaign, the dosage is based on weight and, alternatively on height, as indicated below.

| ***Height (en cm)*** | ***Quantity*** |
| --- | --- |
| ***PEDIATRIC SUSPENSION (ml)*** | |
| *<­­60cm* | *None* |
| *60 – 71* | *4 ml* |
| *72 – 86* | *6 ml* |
| *87 – 98* | *8 ml* |
| *99 – 109* | *10 ml* |
| *110 – 119* | *12 ml* |
| *120 – 128* | *14 ml* |
| *129 – 139* | *16 ml* |
| *TABLET* | |
| *85 – 94* | *1 tablet* |
| *95 – 123* | *2 tablets* |
| *124 – 143* | *3 tablets* |
| *More than 144 cm* | *4 tablets* |

Note*: Tablets can be taken during or in-between meals, in one single daily dose.

**Contraindications:**

Azithromycin is contraindicated for pregnant women and children less than 6 months of age, and in case of hypersensitivity to azithromycin or any other macrolides. Even though animal studies on rodents do not demonstrate any malformation effect, clinical data is insufficient.

*Because azithromycin contains lactose*, it is contra-indicated in case of congenital galactosemia, syndrome of glucose malabsorption, galactose, and lactase deficit.

The safety of azithromycin in lactating women is not established, therefore it should be prescribed only if expected benefits outweigh potential risks.

Since azithromycin is metabolized in the liver, the drug is not recommended for patients suffering from severe liver disease or severe cholestasis.

- *Associations* with oral anticoagulants, ciclosporin, cisapride, ergot derivatives: dihydroergotamine, ergotamine.
**These medicinal products can interact with the way azithromycin affect the body or increase the risks of secondary effects:**

1. antiacids containing aluminium
2. antiacids containing magnesium
3. nelfinavir

**These medicinal products can be affected by azithromycin:**

1. astémizole
2. cisapride
3. cyclosporine
4. digoxine
5. dihydroergotamine
6. disopyramide
7. ergotamine
8. hexobarbital
9. statine (atorvastatine, lovastatine, simvastatine, for instance)
10. phénytoine
11. terfénadine
12. théophylline
13. triazolam
14. warfarine
15. zidovudine

**Other substances**

1. caffeine
2. alcohol
3. nicotine in cigarettes
4. narcotics

**ADVERSE EFFECTS:**

1. **Mucocutaneous and allergic**
   1. Rash
   2. Photosensitivity
   3. Arthralgia
   4. Urticaria
   5. Pruritus
   6. Rarely, angioneurotic edema
   7. *Anaphylaxis*
   8. Severe skin reactions
2. **Gastrointestinal**
   1. Nausea
   2. Vomiting
   3. Dyspepsia
   4. Diarrhea (watery and severe, can be bloody)
   5. Abdominal pain
   6. Pancreatitis
   7. Pseudomembranous colitis
3. **Hepato-billary**
   1. Elevation of liver enzymes
   2. Hepatic necrosis
   3. Liver failure
   4. Cholestatic hepatitis
4. **Nervous system**
   1. Headache
   2. Dizziness/vertigo
   3. Convulsions
5. **Blood**
   1. Thrombocytopenia
6. **Psychiatric**
   1. Aggressiveness
   2. Nervousness
   3. Agitation
   4. Anxiety
7. **Genital**
   1. Vaginitis
8. **Auditory**
   1. Dizziness
   2. Tinnitus
   3. Deafness
9. **General**
10. Candidasis
11. Fever

## APPENDIX I: Technical Note on ivermectin and albendazole

*Summary of Product Characteristics (SPC)*

**PRESENTATION: ALBENDAZOLE**

Albendazole is a benzimidazole carbamate. It is a broad-spectrum anthelminthic agent orally administered. It appears to cause irreversible inhibition of glucose uptake by parasites. Albendazole is available as a 400 mg tablet.

Excipients: lactose, cornstarch, povidone, sodium lauryl sulfate, sodium carboxymethyl starch, microcristalline cellulose, sodium saccharin, magnesium stearate, hypromellose, propylene glycol, alumina hydrate sunset yellow S (E 110).

Aroma: orange, vanilla, Passion fruit

**METABOLISM AND MODE OF ACTION**

Following oral administration, the low proportion of absorbed albendazole (< 5%) is metabolized to albendazole sulfoxide and sulfone. The plasma concentration in sulfoxide, which is the primary circulating active metabolite, is greatest about two and a half hour after administration. The plasma half-life of albendazole sulfoxide is 8 hours and 30 minutes. Albendazole sulfoxide and its metabolites seem to be mainly eliminated through the bile while urinary excretion would appear to be minimal.

Albendazole affects the cytoskeleton of helminthes by inhibiting tubulin polymerisation and their incorporation in the microtubules, thus blocking parasites glucose absorption and killing them. The parasite’s sealabilty to enterocytes is diminished, which leads to growth inhibition and prevent the parasite from multiplying. Albendazole is also active against larvae at the beginning of the tissular migration.

**INDICATIONS:** Albendazole is indicated for the treatment of parenchymal neurocysticercosis due to active lesions caused by larval form of pork tapeworm, *Taenia solium*. It is also indicated for the treatment of cystic hydatid disease of the liver, lung, and peritoneum, caused by the larval form of the tapeworm, *Echinococcus granulosus*.

In association with ivermectin, it affects *Wuchereria bancrofti* and *Brugia malayi* microfilariae. This association has been shown to reduce blood filarial counts and interrupt further transmission (Lancet 1997; 350: 480-484 control study and placebo).

**DOSAGE:** Annual treatment with a single dose of 400 mg of albendazole (or 15 mg/kg) associated with ivermectin for lymphatic filariasis. During mass treatment campaigns, Albendazole is distributed as a standard dose of one tablet per subject.

In young children, the tablets should be crushed or chewed and swallowed with a glass of drinking water. In this study, one 400 mg tablet is given to all subjects measuring at least 90 centimeters.

Its half-life is 8 hours.

**Dosage based on height**

| **Patient’s Height (cm)** | **Albendazole (400 mg tablet)** |
| --- | --- |
| Less than 90 | None |
| 90-119 | 1 tablet |
| 120-140 | 1 tablet |
| 141-158 | 1 tablet |
| 159+ | 1 tablet |

**CONTRA-INDICATIONS**

Pregnancy: Animal studies have demonstrated a teratogenic effect. There is insufficient data regarding excretion of Albendazole in human breast milk.

**SIDE EFFECTS**

They are extremely rare and consist of [vomi](http://www2.biam2.org/www1/SubEIIMCVOMISSEMENT.html)ting, nausea, diarrhea, headache and vertigo. During prolonged treatments, the following side effects have been reported: fever, reversible alopecia, leukopenia, pancytopenia, and increase in transaminases, rash.

Tablet: Risk of allergic reactions due to the colorant sunset yellow S.

**IVERMECTIN PRESENTATION: MECTIZAN***

Ivermectin is available in 6 mg and 3 mg tablets. Expients are: cellulose, cornstarch, magnesium stearate, citric acid, butylhydroxanisol. In this study, Ivermectin is available in 3 mg tablets.

**INDICATIONS**: It is indicated for the treatment of onchocerciasis caused by *Onchocerca volvulus* and lymphatic filariasis in association with albendazole. In this study, 3 mg tablets of Ivermectin will be distributed with albendazole.

**DOSAGE**

One single oral dose administered on an empty stomach, with water. The influence of food on absorption is unknown. The dosage can be taken in the morning or at any other time of the day but no food should be taken within two hours before or after administration. The interval between two administrations is 12 months. However, in some endemic regions, Ivermectin can be taken every 6 months. Dosage is based on weight and alternatively on height during mass treatment campaigns. To treat bancrofti microfilariae, the following concomitant treatment with ivermectin and albendazole is needed:

| Person’s Height (cm) | Person’s Weight (kg) | Ivermectin (3 mg tablet) | Albendazole  (400 mg tablet) |
| --- | --- | --- | --- |
| *Less than 90 cm* | *Less than 15 kg* | *None* | *None** |
| 90-119 cm | 15-25 kg | 1 tablet | 1 tablet |
| 120-140 cm | 26-44 kg | 2 tablets | 1 tablet |
| 141-158 cm | 45-64 kg | 3 tablets | 1 tablet |
| 159 cm and more | 65 kg and more | 4 tablets | 1 tablet |

**CONTRA-INDICATIONS:** Pregnant women and children less than 5 years of age and in case of hypersensitivity to Ivermectin.

When used with children under 5 years of age, Ivermectin safety has not been demonstrated. Animal studies have shown that a repeated use of Ivermectin could lead to several malformations.

Less than 2% of the administered dose of Ivermectin appeared in breast milk. The drug should be given to nursing mothers one week after the birth of the child.

**SIDE EFFECTS:**

Hypersensitivity due to the massive destruction of microfilaria. These side effects include Mazotti type reaction: pruritus, conjunctivitis, arthralgia, abdominal myalgia, fever, lymphadenitis, lymphadenopathies, nausea, vomiting, diarrhea, orthostatic hypotension, tachycardia, asthenia, rash and headache. These signs have rarely been severe.

Ophthalmologic side effects are rare: abnormal sensation in the eyes, eyelid edema, uveitis.

**APPENDIX J: Subject Census Register**

| Date | Census Number | Name and surname | Age | Sex | Surname and Name of Head of the family | Residence | Study Participation | | | | |
| --- | --- | --- | --- | --- | --- | --- | --- | --- | --- | --- | --- |
|  |  |  |  |  |  |  | Agreement | Disagreement | Inclusion | Exclusion | Reason for Exclusion |
|  | 0 0 1 A 0 0 0 1 |  |  |  |  |  |  |  |  |  |  |
|  | /_/_ /_/_/_ /_/_/_/ |  |  |  |  |  |  |  |  |  |  |
|  | /_/_ /_/_/_ /_/_/_/ |  |  |  |  |  |  |  |  |  |  |
|  | /_/_ /_/_/_ /_/_/_/ |  |  |  |  |  |  |  |  |  |  |
|  | /_/_ /_/_/_ /_/_/_/ |  |  |  |  |  |  |  |  |  |  |
|  | /_/_ /_/_/_ /_/_/_/ |  |  |  |  |  |  |  |  |  |  |
|  | /_/_ /_/_/_ /_/_/_/ |  |  |  |  |  |  |  |  |  |  |
|  | /_/_ /_/_/_ /_/_/_/ |  |  |  |  |  |  |  |  |  |  |
|  | /_/_ /_/_/_ /_/_/_/ |  |  |  |  |  |  |  |  |  |  |
|  | /_/_ /_/_/_ /_/_/_/ |  |  |  |  |  |  |  |  |  |  |
|  | /_/_ /_/_/_ /_/_/_/ |  |  |  |  |  |  |  |  |  |  |
|  | /_/_ /_/_/_ /_/_/_/ |  |  |  |  |  |  |  |  |  |  |
|  | /_/_ /_/_/_ /_/_/_/ |  |  |  |  |  |  |  |  |  |  |
|  | /_/_ /_/_/_ /_/_/_/ |  |  |  |  |  |  |  |  |  |  |
|  | /_/_ /_/_/_ /_/_/_/ |  |  |  |  |  |  |  |  |  |  |
|  | /_/_ /_/_/_ /_/_/_/ |  |  |  |  |  |  |  |  |  |  |
|  | /_/_ /_/_/_ /_/_/_/ |  |  |  |  |  |  |  |  |  |  |
|  | /_/_ /_/_/_ /_/_/_/ |  |  |  |  |  |  |  |  |  |  |
|  | /_/_ /_/_/_ /_/_/_/ |  |  |  |  |  |  |  |  |  |  |
|  | /_/_ /_/_/_ /_/_/_/ |  |  |  |  |  |  |  |  |  |  |
|  | /_/_ /_/_/_ /_/_/_/ |  |  |  |  |  |  |  |  |  |  |
|  | /_/_ /_/_/_ /_/_/_/ |  |  |  |  |  |  |  |  |  |  |

Subject Identification Number: Family: **001** Sector: **A** Subject: **0001**

**APPENDIX K**: **Presentation of height poles**

**Azithromycin**

| **Height (cm)** | **Quantity to be given** |
| --- | --- |
| **PEDIATRIC SUSPENSION (ml)** | |
| 60 – 71 | 4 ml |
| 72 – 86 | 6 ml |
| 87 – 98 | 8 ml |
| 99 – 109 | 10 ml |
| 110 – 119 | 12 ml |
| 120 – 128 | 14 ml |
| 129 – 139 | 16 ml |
| **TABLET** | |
| 90 – 94 | 1 tablet |
| 95 – 123 | 2 tablets |
| 124 – 143 | 3 tablets |
| More than 144 | 4 tablets |

**Ivermectin+albendazole**

| **Height of patient (cm)** | **Ivermectin 3 mg tablet** | **Albendazole 400 mg tablet** |
| --- | --- | --- |
| 159+ | 4 tablets | 1 tablet |
| 141-158 | 3 tablets | 1 tablet |
| 120-140 | 2 tablets | 1 tablet |
| 90-119 | 1 tablet | 1 tablet |
| Less than 90 | None | None |

**Double-sided height pole and height pole for children not included in the study**

**Height pole for children not included in the study**

**Height pole for children and adults included in the study**

| TRACHOMA | | TRACHOMA | | FILARIOSE | | |
| --- | --- | --- | --- | --- | --- | --- |
| NIEDIALAN DIMI | | NIEDIALAN DIMI | | SEN BA | | |
| **AZI**  **Azithromycin**  **SUSPENSION** | |  | **AZI**  **Azithromycin**  **TABLET** |  | **IVM**  **Ivermectin** | **ALB**  **Albendazole** |
|  |  | **HEIGHT** | **DOSE** | **HEIGHT** | **DOSE** | **DOSE** |
|  |  | Over 144 | **4 tablets** | Over 159 | 4 tablets | 1 tablet |
|  |  |  |  |  |  |  |
|  |  |  |  | 141-158 | 3 tablets | 1 tablet |
| **HEIGHT** | **DOSE** |  |  |  |  |  |
| 129 – 139 | 16 ml | 124-143 | **3 tablets** | 120-140 | 2 tablets | 1 tablet |
| 120 – 128 | 14 ml |  |  |  |  |  |
| 110 – 119 | 12 ml | 95-123 | **2 tablets** | 90-119 | 1 tablet | 1 tablet |
| 99 – 109 | 10 ml |  |  |  |  |  |
| 87 – 98 | 8 ml | 90-94 | **1 tablet** |  |  |  |
|  |  |  |  |  |  |  |
|  | | | | | |  |
| 72 – 86 | 6 ml | Not included in the study | | Not included in the study | | |
| 60 – 71 | 4 ml |  |  |  |  |  |
| Less than 60 | Tétra 1% |  |  |  |  |  |
