# Supplementary material for: A Cluster Randomized Study of The Safety of Integrated Treatment of Trachoma and Lymphatic Filariasis in Children and Adults in Sikasso, Mali
Source: PLoS Negl Trop Dis. 2013 May 9;7(5):e2221. doi: 10.1371/journal.pntd.0002221 (PMC3649960; doi:10.1371/journal.pntd.0002221)
Supplement: Text S2 — Ethics Approval. Approval (in French) of Version 2 of protocol issued on 5th October 2009 by the Ethics Committee of the Faculty of Medicine, Pharmacy and Dentistry of the University of Bamako, Mali. (PDF) [file pntd.0002221.s005.pdf]

Ministère de santé
Sécrétariat Général
Centre National d'Appui à
la lutte contre la Maladie (CNAM)

République du Mali
Un Peuple-Un But-Une Foi

REF.No. 109 /AZIVAL

Bamako, le 29 septembre 2009

A

Monsieur le Président
du Comité d'Ethique
de la FMPOS, Bamako, Mali.

Objet : **Soumission de modifications de protocole au Comité d'Ethique de la FMPOS du Mali.**

Monsieur le Président,

J'ai l'honneur de vous soumettre les amendements apportés au protocole d'étude ci-joint intitulé : « **Etude de pharmacovigilance sur l'innocuité du traitement intégré du trachome et de la filariose lymphatique chez les enfants et les adultes vivant dans la région de Sikasso au Mali** » Version 2.0 du 08/08/09.

Ces amendements concernent la mise à jour des données sur la cartographie du trachome et de la filariose lymphatique de juin 2008 dans la région de Sikasso et ont porté sur les points suivants:

1. Le deuxième paragraphe de la section 1.2.1 de l'introduction en page 7 du protocole.
2. Le dernier point de la page 8 incluant les tableaux 2 et 3 du protocole.

Je vous prie, Monsieur le Président, de croire à l'expression de mes sentiments respectueux.

Le Directeur Général - CVD - Mali
Le Pr. SAMBA O. SOW

Samba O. SOW, MD, MSc.

PJ : 4 copies du protocole (version 2.0 du 8 Août 2009).

CENTRE NATIONAL D'APPUI A LA LUTTE CONTRE LA MALADIE (CNAM)
MINISTERE DE LA SANTE
Ex-INSTITUT MARCHOUX, BP 251, BAMAKO, MALI
Téléphone + (223) 20 23 5131 *Fax* (223) 20 22 2845
ssow@medicine.umaryland.edu

MINISTERE DE L'ENSEIGNEMENT
SUPERIEUR ET DE LA RECHERCHE SCIENTIFIQUE

UNIVERSITE DE BAMAKO

FACULTE DE MEDECINE DE PHARMACIE ET
D'ODONTO-STOMATOLOGIE / BP 1805

☎ : (223) 20 22 52 77 🖷 : (223) 20 22 96 58

BAMAKO – MALI

N°09___67_____/FMPOS

Le Président du Comité
D'Ethique de la FMPOS

Bamako, le 05 Octobre 2009

/-)u **Professeur Samba Ousmane SOW**
MD ; MSc. CVD Mali

Cher Professeur,

J'ai le plaisir de vous informer que votre projet de recherche intitulé : **«Etude de pharmacovigilance sur l'innocuité du traitement intégré du trachome et de la filariose lymphatique chez les enfants et les adultes vivant dans la région de Sikasso au Mali»** a été examiné par le Comité d'Ethique de la Faculté de Médecine, de Pharmacie et d'Odonto-Stomatologie de l'Université de Bamako en comité restreint.
Les nouveaux amendements indiqués dans votre lettre du 29/10/09 à la version 2.0 ont été approuvés car ils ne modifient en rien la qualité des résultats.

Le Comité Institutionnel d'Ethique de la FMPOS vous souhaite plein succès.

LE PRESIDENT
DU COMITE D'ETHIQUE

Faculté de Médecine et de Pharmacie — Le President — Comité d'Ethique

Prof. Mamadou Marouf KEITA
